# Investigation of antituberculosis, antimicrobial, anti-inflammatory efficacies of newly synthesized transition metal(II) complexes of hydrazone ligands: structural elucidation and theoretical studies

**DOI:** 10.1038/s41598-023-42180-4

**Published:** 2023-09-23

**Authors:** Binesh Kumar, Jai Devi, Amit Dubey, Aisha Tufail, Bharti Taxak

**Affiliations:** 1 Department of Chemistry, Guru Jambheshwar University of Science and Technology, Hisar, Haryana 125001 India; 2 Department of Pharmacology, Saveetha Dental College and Hospital, Saveetha Institute of Medical and Technical Sciences, Chennai, Tamil Nadu 600077 India; 3 Department of Computational Chemistry and Drug Discovery Division, Quanta Calculus, Greater Noida, Uttar Pradesh India

**Keywords:** Computational biology and bioinformatics, Chemistry, Coordination chemistry, Inorganic chemistry, Medicinal chemistry

## Abstract

Tuberculosis disease is a serious threat to humans and spreading quickly worldwide, therefore, to find a potent drug, the synthesis of hydrazone ligands endowed Co(II), Ni(II), Cu(II), Zn(II) metal complexes were carried out and well characterized by numerous spectral and analytical techniques. The octahedral geometry of the complexes was confirmed by spectral analysis. Further, in vitro antituberculosis efficacy of the compounds **(1–10)** revealed that complexes **(6), (9), (10)** have highest potency to control TB malformation with 0.0028 ± 0.0013–0.0063 ± 0.0013 µmol/mL MIC value while Zn(II) complex **(10)** (0.0028 ± 0.0013 µmol/mL) has nearly four time potent to suppress TB disease in comparison of streptomycin (0.0107 ± 0.0011 µmol/mL). The antimicrobial and anti-inflammatory evaluations revealed that the complex **(10)** is more active with lowest MIC (0.0057–0.0114 µmol/mL) and IC_50_ (7.14 ± 0.05 µM) values, correspondingly which are comparable with their respective standard drugs. Furthermore, the theoretical studies such as molecular docking, DFT, MESP and ADMET were employed to authenticate the potency of **HL**^**2**^ hydrazone ligand **(2)** and its metal complexes **(7–10)** which revealed that the zinc(II) complex **(10)** might be utilized as novel drug candidate for tuberculosis dysfunctions. So, the present research gives a new insight for in vivo investigation of the compounds.

## Introduction

Tuberculosis (TB) is a leading cause of death worldwide which is a bacillus *Mycobacterium tuberculosis*-based ailment and spreads when a TB patient release bacteria in air by coughing. The global TB report 2022 of the WHO demonstrates how the COVID-19 pandemic harmed tuberculosis diagnosis and unachieved target of the TB treatments worldwide^1^. The report analysis indicates that the undiagnosed and untreated TB patients increased in 2020 and 2021 in comparison of 2019 and currently, the TB deaths are doubled in comparison of the AIDS/HIV deaths. In future, TB will be one of the leading cause of mortality and morbidity globally by a single infectious agent, replacing COVID-19^2^. TB also weakens the immunity of the patients and creates a lot of chances of other infectious ailments such as microbial and inflammation which is an awful situation for the patients^3^, so, there is significant need to develop an effective and safer drug. Hence on viewing the above situation, the synthesized transition metal complexes of hydrazone ligands were analyzed against anti-TB, antimicrobial and anti-inflammatory activities, in the hope of getting a significant therapeutic agent.

The utilization of metal complexes for treatment of health problems is an ancient practice but the investigation of structural characteristics of inorganic complexes for medicinal chemistry has advanced significantly. The ability of metal-based drugs to penetrate the microbial membrane and bind with genetic materials (RNA/DNA) of these pathogenic microbes, is a critical feature. Currently, transition metal complexes are a celebrated class of medicinal chemistry to discover novel biological agents with desired medicinal requirements. First row transition metal complexes have high nucleobase ability, more DNA binding efficacy and also have higher receptor binding capability. So, by analyzing the current scenario of biomolecules and metals, we choose the Co(II), Ni(II), Cu(II) and Zn(II) metals for the present research because these are recognized as promising anti-infectious agents^4^ due to their structural versatility, low toxicity, chelation, solubility effect, enzymatic action, interaction with proteins, high penetrating power, permeability, lipophilic character etc. Among the ligand scaffolds, researchers paid specific attention toward hydrazone ligands because of their well-known structural adaptability, chelating ability and wide variety of medicinal properties^5^. Thus, the metal complexes of hydrazone ligand are also broadly studied in medicinal chemistry with the purpose to discover a safe and effective therapeutic drug to cure numerous pathogenic diseases. Literature shows that these metal complexes have numerous pharmaceutical applications such as antimicrobial^6^, anti-TB^7,8^, anticancer^9,10^, antioxidant^11^, anti-inflammatory^12^, antimalarial^13^ etc. Further, the medicinal chemist faces a lot of problems to develop an effective drug with minimal disadvantages, therefore, in silico studies are worth mentioning to overcome these issues.

In the current research, the numerous theoretical studies like molecular docking, DFT, MESP, ADMET are used by the researchers to validate in vitro results. The molecular docking gives valuable information about the bonding ability of the drug with effective site of the protein by availing the binding score and interactions^14^. DFT study is a leading methodology for chemical compound simulation and modelling which was carried out to understand the relationship between the consistency, structural system, global surface chemistry. The primary goal of DFT analysis is to recognize the biologically active agent amid the tested compounds by utilizing quantum mechanical principles. The DFT calculations are mainly utilized for investigating and describing the essential structural information and biological characteristics by gaining an understanding of electronic properties, stability, HOMO–LUMO gap, hardness, softness, electronegativity, reacting sites etc.^15–18^. The MESP study analyzed the bond order, size, negative area, neutral electrostatic potential, positive area etc. which are significant parameters of the medicinal chemistry^19^. The ADMET prediction^20^ helps a lot by providing numerous important criteria about the drug like human intestinal absorption (HIA), water solubility, blood–brain barrier (BBB) penetration etc.

Our continuous efforts in the area of pharmaceutical chemistry and by motivating from the above facts, herein, the synthesis of two hydrazone ligands and their transition metal complexes from 3,5-bis(trifluoromethyl)benzohydrazide and benzaldehyde derivatives were carried out. Various physio-analytical methods were used to characterize the synthesized compounds. In the hope of an effective therapeutic agent, in vitro anti-tuberculosis, antimicrobial and anti-inflammatory activities were carried out. Furthermore, theoretical studies were executed to validate the biological results of the highly active **HL**^**2**^ ligand and its complexes (7–10).

## Experimental

### Materials

The AR grade chemicals and reagents including 2-methoxy-1-napthaldehyde (> 99%), 3,5-bis(trifluoromethyl)benzohydrazide (> 99%), 3-bromo-5-ethoxy-4-hydroxybenzaldehyde (> 99%), cobalt(II) acetate tetrahydrate (≥ 98%), nickel(II) acetate tetrahydrate (≥ 98%), copper(II) acetate monohydrate (≥ 98%) and zinc(II) acetate dihydrate (≥ 98%) were used as such as acquired from Sigma Aldrich firm.

### Instrumentations

The compounds were FTIR analyzed on a Perkin Elmer BX III spectrometer utilizing KBr pellets. The Avance III 400 MHz:Bruker NMR apparatus was utilized to obtain the NMR spectral data of the zinc(II) complexes and their associated hydrazone ligands. The JES-FA200 ESR spectrometer with X-band was implemented to obtained the ESR spectral data of copper(II) complexes using a standard (tetracyanoethylene) and 3000 gauss magnetic field. At 298 K, the molar conductance was measured in DMF solvent on a Systronic conductivity bridge model-306. The Perkin Elmer Diamond apparatus was utilized to assess the thermal data of the compounds in the environment of pure argon gas taking alumina as standard. The melting point was determined using the hot stage Gallenkamp apparatus in open capillaries. The mass spectral data was obtained using a SCIEX Triple TOF 5600 spectrometer and a solvent (acetonitrile). The compounds' UV–Vis data was evaluated in THF on UV–Vis-NIR Varian Carry 5000 instrument and in solidified barium sulfate on UV-3600 plus spectrophotometer. The standard gravimetric method was implemented to evaluate metal contents in complexes, utilizing cobalt pyridine thiocyanate for Co, cupreous thiocyanate for Cu, nickel dimethylglyoximate for Ni and zinc ammonium phosphate for Zn. Powder XRD was analyzed on a Rigaku Miniflex-II using Cu-Kα (1.54) radiation. The JEOL 7610F plus apparatus was used to examine the SEM and EDAX micrographs. The magnetic susceptibility was assessed on vibrating sample magnetometer using Hg[Co(SCN)4] as the calibrant.

### Synthetic protocol of hydrazone ligands (HL^1^–HL^2^) (1–2) and their complexes (3–10)

The hydrazone ligands **(HL**^**1**^**–HL**^**2**^**)** were synthesized by refluxing 30 mL methanolic solution of 3,5-bis(trifluoromethyl)benzohydrazide (5.0 mmol, 1.360 g) for 5–6 h with 2-methoxy-1-napthaldehyde (5.0 mmol, 0.931 g)/ /3-bromo-5-ethoxy-4-hydroxybenzaldehyde (5.0 mmol, 1.225 g) on addition of glacial acetic acid (0.1 mL). TLC was utilized to evaluate the progress of the reaction. To purify the compounds, the obtained white colored solids were washed with hexane and recrystallized in methanol.

**Figure 1:**
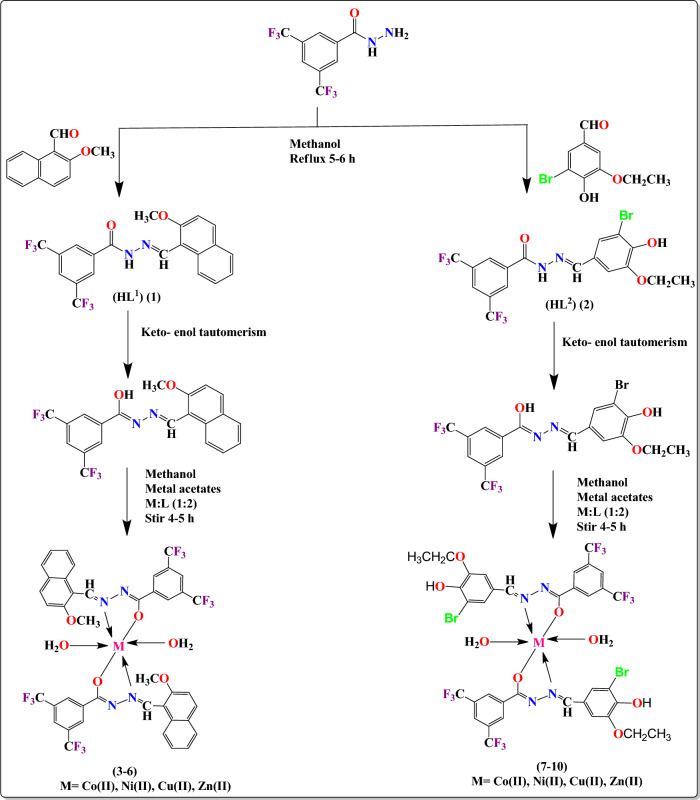
Synthesis of **(HL**^**1**^**–HL**^**2**^**)** hydrazone ligands **(1–2)** and their transition metal complexes** (3–10)**.

Further, complexation was accomplished by stirring 20 mL methanolic solution of obtained hydrazone ligands (**HL**^**1**^**–HL**^**2**^, 2.0 mmol) for 4–5 h with Co(II), Ni(II), Cu(II), Zn(II) acetates (1.0 mmol) in 2:1 molar ratio. Then, the obtained distinct colored solids were washed with methanol and recrystallized in tetrahydrofuran (Fig. 1).

### Analytical data of the compounds (1–10)

1. HL^1^ ligand (1)

Color: white; yield: 82%; M.p.: 110–113 °C. Conductivity (ohm^−1^ cm^2^ mol^−1^) in DMF: 10. Anal. found for C_21_H_14_F_6_N_2_O_2_ (%): C, 57.28; H, 3.20; N, 6.36. Calcd. (%): C, 57.29; H, 3.21; N, 6.37. IR (KBr, cm^−1^): 1598 *ʋ*(–HC=N–), 3230 *υ*(N–H), 1647 *ʋ*(C=O)_keto**nic**_, `1049 *ʋ*(N–N). MS for C_21_H_14_F_6_N_2_O_2_ *m/z*: 440.0959, [M + H]^+^: 441.1037. ^1^H NMR (400 MHz, DMSO-*d*_*6*_) δ 12.28 (s, 1H, –NH), 9.42 (s, 1H, –HC=N–), 9.20 (s, 1H, Ar–H), 8.65 (s, 2H, Ar–H), 8.41–8.36 (dd, *J* = 7.1, 1.6 Hz, 1H, Ar–H), 8.11–8.08 (d, *J* = 7.1 Hz, 1H, Ar–H), 7.95–7.93 (d, *J* = 7.9 Hz, 1H, Ar–H), 7.63–7.54 (m, 2H, Ar–H), 7.48–7.44 (dd, *J* = 7.4, 1.4 Hz, 1H, Ar–H), 4.04 (s, 3H, –OCH_3_). ^13^C NMR (100 MHz, DMSO-*d*_*6*_) δ 160.56 (–C=O–), 158.65 (–HC=N–), 147.20, 136.19, 133.71, 131.18, 130.85, 129.22, 129.02, 128.70, 127.88, 127.66, 126.29, 125.70, 124.95, 124.62, 122.23, 114.27, 113.88, 57.17 (–OCH_3_).

2. HL^2^ ligand (2)

Color: white; Yield: 84%; M.p.: 108–110 °C. Conductivity (ohm^−1^ cm^2^ mol^−1^) in DMF: 11. Anal. found for C_18_H_13_BrF_6_N_2_O_3_ (%): C, 43.31; H, 2.62; N, 5.61. Calcd. (%): C, 43.32; H, 2.63; N, 5.62. IR (KBr, cm^−1^): 1597 *ʋ*(–HC=N–), 3237 *υ*(N–H), 1656 *ʋ*(C=O)_keto**nic**_, 1042 *ʋ*(N–N). MS for C_18_H_13_BrF_6_N_2_O_3_ *m/z*: 497.9997, [M + H]^+^: 499.0075. ^1^H NMR (400 MHz, DMSO-*d*_*6*_) δ 12.17 (s, 1H, NH), 9.86 (s, 1H, OH), 8.55 (s, 1H, –HC=N–), 8.47 (s, 1H, Ar–H), 8.38 (s, 1H, Ar–H), 8.32 (s,1H, Ar–H), 7.47 (s, 1H, Ar–H), 7.35 (s, 1H, Ar–H), 4.19–4.14 (q, 2H, –OCH_2_–), 1.42–1.39 (t, 3H, –CH_3_). ^13^C NMR (100 MHz, DMSO-*d*_*6*_) δ 160.58 (–C=O–), 159.28 (–HC=N–), 148.63, 148.28, 136.30, 135.18, 134.89, 131.17, 130.84, 128.92, 126.75, 125.12, 125.09, 109.96, 109.92, 65.22 (–OCH_2_), 14.98 (–CH_3_).

3. [Co(L^1^)_2_(H_2_O)_2_] complex (3)

Color: green; Yield: 77%; M.p.: 221–223 °C. Conductivity (ohm^−1^ cm^2^ mol^−1^) in DMF: 14. Anal. found for C_42_H_30_F_12_N_4_O_6_Co (%): C, 51.81; H, 3.11; N, 5.75; M, 6.05. Calcd. (%): C, 51.82; H, 3.13; N, 5.78; M, 6.06. IR (KBr, cm^−1^): 1595 *ʋ*(–HC=N–), 1277 *ʋ*(C–O)_enolic_, 1035 *ʋ*(N–N), 534 *ʋ*(M–O), 471 *ʋ*(M–N), 3455 *ʋ*(–OH _Water_). MS for C_42_H_30_F_12_N_4_O_6_Co *m/z*: 973.5809, [M + H]^+^: 974.5887.

4. [Ni(L^1^)_2_(H_2_O)_2_] complex (4)

Color: brown; Yield: 75%; M.p.: 223–225 °C. Conductivity (ohm^−1^ cm^2^ mol^−1^) in DMF: 18. Anal. found for C_42_H_30_F_12_N_4_O_6_Ni (%): C, 51.82; H, 3.11; N, 5.76; M, 6.03. Calcd. (%): C, 51.83; H, 3.12; N, 5.77; M, 6.05. IR (KBr, cm^−1^): 1593 *ʋ*(–HC=N–), 1295 *ʋ*(C–O)_enolic_, 1032 *ʋ*(N–N), 537 *ʋ*(M–O), 473 *ʋ*(M–N), 3450 *ʋ*(–OH _Water_). MS for C_42_H_30_F_12_N_4_O_6_Ni *m/z*: 972.4327, [M + H]^+^: 973.4405.

5. [Cu(L^1^)_2_(H_2_O)_2_] complex (5)

Color: dark green; Yield: 79%; M.p.: 228–230 °C. Conductivity (ohm^−1^ cm^2^ mol^−1^) in DMF: 20. Anal. found for C_42_H_30_F_12_N_4_O_6_Cu (%): C, 51.57; H, 3.09; N, 5.73; M, 6.50. Calcd. (%): C, 51.60; H, 3.11; N, 5.75; M, 6.52. IR (KBr, cm^−1^): 1597 *ʋ*(–HC = N–), 1281 *ʋ*(C–O)_enolic_, 1037 *ʋ*(N–N), 539 *ʋ*(M–O), 469 *ʋ*(M–N), 3459 *ʋ*(–OH _Water_). MS for C_42_H_30_F_12_N_4_O_6_Cu *m/z*: 977.1270, [M + H]^+^: 978.1348.

6. [Zn(L^1^)_2_(H_2_O)_2_] complex (6)

Color: yellow; Yield: 80%; M.p.: 210–212 °C. Conductivity (ohm^−1^ cm^2^ mol^−1^) in DMF: 17. Anal. found for C_42_H_30_F_12_N_4_O_6_Zn (%): C, 51.47; H, 3.09; N, 5.72; M, 6.67. Calcd. (%): C, 51.49; H, 3.12; N, 5.73; M, 6.69. IR (KBr, cm^−1^): 1592 *ʋ*(–HC=N–), 1279 *ʋ*(C–O)_enolic_ 1042 *ʋ*(N–N), 536 *ʋ*(M–O), 466 *ʋ*(M–N), 3452 *ʋ*(–OH _Water_). MS for C_42_H_30_F_12_N_4_O_6_Zn *m/z*: 977.9936, [M + H]^+^: 979.0014. ^1^H NMR (400 MHz, CDCl_3_) δ 9.02 (s, 1H, –HC=N–), 8.82 (s, 2H, Ar–H), 8.01 (s, 1H, Ar–H), 7.55–7.53 (d, *J* = 7.1 Hz, 1H, Ar–H), 7.36–7.34 (d, *J* = 6.5 Hz, 2H, Ar–H), 7.23–7.19 (dd, *J* = 6.8, 1.6 Hz, 1H, Ar–H), 7.16–7.13 (dd, *J* = 5.2, 1.3 Hz, 1H, Ar–H), 7.06–7.04 (d, *J* = 6.2 Hz, 1H, Ar–H), 3.77 (s, 3H, –OCH_3_). ^13^C NMR (100 MHz, CDCl_3_) δ 169.23 (–C–O–), 164.92 (–HC=N–), 155.34, 151.53, 137.65, 133.52, 131.70, 131.59, 131.26, 128.61, 128.41, 127.70, 124.42, 123.97, 122.10, 121.74, 113.09, 112.62, 57.12 (–OCH_3_).

7. [Co(L^2^)_2_(H_2_O)_2_] complex (7)

Color: brown; Yield: 79%; M.p.: 215–217 °C. Conductivity (ohm^−1^ cm^2^ mol^−1^) in DMF: 17. Anal. found for C_36_H_28_Br_2_F_12_N_4_O_8_Co (%): C, 39.62; H, 2.59; N, 5.13; M, 5.40. Calcd. (%): C, 39.63; H, 2.60; N, 5.15; M, 5.42. IR (KBr, cm^−1^): 1592 *ʋ*(–HC=N–), 1278 *ʋ*(C–O)_enolic_, 1025 *ʋ*(N–N), 532 *ʋ*(M–O), 471 *ʋ*(M–N), 3452 *ʋ*(–OH _Water_). MS for C_36_H_28_Br_2_F_12_N_4_O_8_Co *m/z*: 1090.9394, [M + H]^+^: 1091.9472.

8. [Ni(L^2^)_2_(H_2_O)_2_] complex (8)

Color: red; Yield: 78%; M.p.: 214–216 °C. Conductivity (ohm^−1^ cm^2^ mol^−1^) in DMF: 18. Anal. found for C_36_H_28_Br_2_F_12_N_4_O_8_Ni (%): C, 39.63; H, 2.59; N, 5.13; M, 5.38. Calcd. (%): C, 39.65; H, 2.61; N, 5.15; M, 5.41. IR (KBr, cm^−1^): 1593 *ʋ*(–HC=N–), 1285 *ʋ*(C–O)_enolic_, 1032 *ʋ*(N–N), 530 *ʋ*(M–O), 464 *ʋ*(M–N), 3445 *ʋ*(–OH _Water_). MS for C_36_H_28_Br_2_F_12_N_4_O_8_Ni *m/z*: 1089.9415, [M + H]^+^: 1090.9493.

9. [Cu(L^2^)_2_(H_2_O)_2_] complex (9)

Color: reddish brown; Yield: 73%; M.p.: 218–220°C. Conductivity (ohm^−1^ cm^2^ mol^−1^) in DMF: 16. Anal. found for C_36_H_28_Br_2_F_12_N_4_O_8_Cu (%): C, 39.45; H, 2.58; N, 5.11; M, 5.80. Calcd. (%): C, 39.46; H, 2.60; N, 5.14; M, 5.82. IR (KBr, cm^−1^): 1595 *ʋ*(–HC=N–), 1287 *ʋ*(C–O)_enolic_, 1039 *ʋ*(N–N), 535 *ʋ*(M–O), 468 *ʋ*(M–N), 3442 *ʋ*(–OH _Water_). MS for C_36_H_28_Br_2_F_12_N_4_O_8_Cu *m/z*: 1094.9358, [M + H]^+^: 1095.9436.

10. [Zn(L^2^)_2_(H_2_O)_2_] complex (10)

Color: yellow; Yield: 72%; M.p.: 219–221 °C. Conductivity (ohm^−1^ cm^2^ mol^−1^) in DMF: 19. Anal. found for C_36_H_28_Br_2_F_12_N_4_O_8_Zn (%): C, 39.39; H, 2.57; N, 5.10; M, 5.96. Calcd. (%): C, 39.40; H, 2.60; N, 5.12; M, 5.98. IR (KBr, cm^−1^): 1591 *ʋ*(–HC=N–), 1292 *ʋ*(C–O)_enolic_, 1035 *ʋ*(N–N), 534 *ʋ*(M–O), 473 *ʋ*(M–N), 3447 *ʋ*(–OH _Water_). MS for C_36_H_28_Br_2_F_12_N_4_O_8_Zn *m/z*: 1095.9353, [M + H]^+^: 1096.9431. ^1^H NMR (400 MHz, DMSO-*d*_*6*_) δ 8.90 (s, 1H, OH), 7.86 (s, 1H, CH=N–), 7.72 (s, 1H, Ar–H) 7.65 (s, 2H, Ar–H), 7.31 (s, 1H, Ar–H), 7.21 (s, 1H, Ar–H), 3.71–3.66 (q, 2H, –OCH_2_), 1.24–1.21 (t, 3H, –CH_3_). ^13^C NMR (100 MHz, DMSO–*d*_*6*_) δ 176.03 (–C–O–), 163.18 (–HC=N–), 160.77, 148.06, 146.50, 143.04, 141.42, 130.00, 126.64, 124.75, 122.26, 122.23, 113.27, 113.02, 111.68, 110.14, 65.35 (–OCH_2_), 14.99 (–CH_3_).

### Biological experiments

In vitro the micro plate alamar blue, serial dilution and BSA assays were carried out against the compounds **(1–10)** in an effort to identify a significant agent for infectious ailments.

### Anti-tuberculosis (TB) activity

The compounds **(1–10)** were examined for anti-tuberculosis evaluation against *Mycobacterium tuberculosis* H_37_R_v_ strain (ATCC No- 27294) by micro plate alamar blue protocol in triplicates by taking streptomycin as standard. The test solution of 0.8–100 μg/mL concentrations were prepared from a stock solution of 1000 μg/mL. The Middlebrook 7HP broth (100 μL) and de-ionized water (200 μL) were taken in sterile 96 well plate which already have test solutions of different concentrations. Then sealed the plate with parafilm and kept in incubation for five days at 37 °C. After that sterile 96 well plate was again incubated for one day on insertion of 25 μL of Tween 10% and 80% and almar blue (1:1 ratio)^21,22^. Further, MIC values were reported as indication of pink color in well plate which exhibited growth of *Mycobacterium* and blue color in well plate showed no bacterial growth.

### Antimicrobial activity

The serial dilution method was utilized to analyze the antimicrobial potency of the compounds **(1–10)** in triplicates against *B. subtilis* (NCIM 2063), *C. albicans* (MTCC 227), *R. oryzae* (MTCC 262), *P. aeruginosa* (MTCC 424), *E. coli* (MTCC 732) and *S. aureus* (MTCC 2901) microbes using standard drugs (fluconazole and ciprofloxacin as positive control) and DMSO (negative control). The 100 μg/mL stock solution was attained by mixing 1 mL of 1000 μg/mL concentration (1 mg in 1 mL DMSO) solution in 9 mL DMSO. After that, the 1 mL of broth (nutrient broth and potato dextrose broths) was mixed with 1 mL stock solutions in test tubes and serially diluted upto 3.12 μg/mL. Then inserted the microbial strains in above prepared test tubes and left them in incubator for a specific time^23,24^ for growth of pathogens. The MIC values (μmol/mL) were determined by checking the progress of strains visually and compared with standard drugs.

### Anti-inflammatory activity

The anti-inflammation behavior of the compounds **(1–10)** was examined by the bovine serum albumin (BSA) assay using standard drug (diclofenac sodium) in triplicate manner for the different concentrations (12.5, 25, 50, 100, 200 µg/mL) of the compounds in DMSO with diluted phosphate buffer (0.2 M, pH 7.4). Then mixed 4 mL of tested solutions with 1 mL of BSA (1 mM) in phosphate buffer. The obtained solutions were put in incubation for 20 min at 37 °C followed by BSA denaturation for 15 min in water bath at 70 °C temperature. On cooling, turbidity appeared in the samples at ambient temperature^25^. Using the percentage denaturation as control without any drug, molar absorbance was assessed at 660 nm by UV-spectrophotometer which is utilized to calculate the % BSA inhibition by the Eq. (1):

1 $${\text{Percentage}}\;{\text{BSA}}\;{\text{inhibition}} = \left( {{1} - {\text{X}}/{\text{Y}}} \right) \times {1}00$$

X and Y shows the molar absorbance of the control and compounds, respectively. The obtained % BSA inhibition data was used to calculate IC_50_ values of the compounds.

### Computational techniques

The computational approaches are currently being employed to address the myriad problems of the pharmaceutical sector, so, below mentioned studies were carried out against highly potent **HL**^**2**^ ligand **(2)** and its complexes **(7–10**). In the present quantum calculations for geometrical optimizations, the B3LYP-D3BJ/def2-TZVP level for the ligand, at the B3LYP/6-31G++ (*d*, *p*) level, and the B3LYP-D3BJ/def2-TZVP (for all atoms except Ni and Cu)+ LANL2DZ (for Ni and Cu) for the metal complexes were used. The values used as criteria for SCF are SCF (CONVER = 8) which translates to 1.00D-08 in convergence on the RMS density matrix and 1.00D-06 in convergence in energy change.

### Molecular docking

In investigations of the molecular recognition process, molecular docking is a valuable computing technique^26^. It emulates the ways of connection, structure, position and estimation of the interaction energies using three-dimensional settings^27^. This was done using two components: potential energy functioning which is typically linked to a force field; and an analysis procedure, which evaluate the minimum global energy for the compound from ligand conformational space sampling^28^. The efficacy of therapeutic interventions depends on the active configurations of metallic complexes exhibiting geometrical and biochemical similarity at the target receptor binding site, which was also examined^29^. The possible interactions among the compounds **(2, 7–10)** and the target protein 5V3Y (Mtb Pks13 thioesterase domain from *Mycobacterium* TB)^30^ were estimated using the MOLEGRO Virtual Docker programmes (Molexus IVS, Odder, Denmark). The MOLEGRO analysis were performed according to the protocol for obtaining the protein receptor from the PDB library^31^, preparing the ligand-receptor, detecting cavities, setting up the search space and docking simulation with other default parameters^32–35^. The binding modalities and position of the docked compounds in the active region of a protein receptor have been evaluated using the docking energies and hydrogen bonds formed using the residues of amino acids from the group of interactions.

### DFT analysis

The DFT approach was utilized to determine the geometric optimization of compounds. To determine the quantum reactivity and for electronic characterization, descriptors need to comprehend the overall response of the molecule's behavior as an electrophile or nucleophile using the FMO (Frontier Molecular Orbitals) approach^36^. The Fukui functions may also be used to find out localized reactivity because they provide an understanding of which atoms participate substantially to the reactivity of the molecule^37^. Since, these methods make it possible to identify the parts of the molecule that are most reactive for interacting with the target protein, they may be used to connect the molecular biological activity. One of the best ways to study the reactivity and stability of chemical species is through this method. The DFT calculations was performed on Gaussian 09 program^38^ while Gauss View 5 program^39^ was utilized to design the input molecules for the DFT quantum calculations to determine reactive characteristics and ground state electrical of **(2, 7–10)** compounds. The streptomycin was made more efficient so that it could be used as standard drug for determining the reactive and electrical characteristics of the compounds. Because of the various orbital energies that these molecules contributed, the optimized geometrical structure was created using the restricted Kohn–Sham functional, Becke's three-parameter functional (B3) and Lee–Yang–Parr gradient corrected correlation functional (LYP) for the exchange part^40–43^. A B3LYP/6-31G++ (*d*, *p*)^22^ polarized base was utilized to create a safe basis set. Additionally, the optimization was carried out by utilizing a number of implicit solvents that were included in the Gaussian 09 software package.

### MESP analysis

The molecular electrostatic properties of the compounds **(2, 7–10)** were evaluated through dipole moment, HOMO, LUMO energy etc. The molecule's van der Waals (vdW) contact surface area was used to determine the electrostatic potentials. The color-coded surface values indicates the positive electrostatic potentials and total molecular size^25^.

### ADMET analysis

ADMET characteristics of five compounds **(2, 7–10)** and streptomycin were computed and reckoned utilizing Discovery Studio 2020. The properties listed by ADMET analysis are deadly intestinal immersion (HIA), tube protein, aqueous solubility, hepatotoxicity etc. The toxicological parameters such as rat oral LD_50_, rodent carcinogenicity, mutagenicity and aerobic biodegradability were examined utilizing the “TOPKAT” module in Discovery Studio^25,44^.

## Results and discussion

### Chemistry

In the current research, eight transition metal complexes bearing hydrazone ligands of 2-methoxy-1-napthaldehyde, 3,5-bis(trifluoromethyl)benzohydrazide and 3-bromo-5-ethoxy-4-hydroxybenzaldehyde were synthesized. The compounds **(1–10)** are soluble in acetonitrile, DMSO, THF, CDCl_3_ etc. but insoluble in water. The molar conductivity measurement states that non-electrolytic behavior of the compounds while amorphous nature and stability of the complexes upto 175 °C were affirmed by powder XRD and TGA, respectively. The octahedral geometry and binding site via N- and O-atoms of the hydrazone ligands with metal(II) ions in bidentate manner was affirmed through numerous spectral analysis as represented in Fig. 1.

### Spectral and Physical techniques

For ascertaining the binding nature of the ligands, structure of the complexes, purity, surface morphology, decomposition of the complexes etc., the various analytical studies were performed as mentioned below.

### Elemental analysis

The elemental composition and purity of the compounds **(1–10)** was evaluated by elemental analysis (C, H, N, M)^3^ which is mentioned in supplementary Table S1 and analytical data of the compounds.

### Molar conductance

The 1 × 10^−3^ M dimethylformamide (DMF) solution of the compounds was used to evaluate the molar conductance. The obtained molar conductivity values (10–20 Ω^−1^ cm^2^ mol^−1^) confirmed the non-electrolytic behavior of the compounds **(1–10)**^45^ (supplementary Table S1).

### Mass spectra

The mass spectra was used to authenticate the formation of compounds by corelating the obtained molecular ion peaks (m/z) with the molecular mass of the compounds. The **HL**^**1**^ and **HL**^**2**^ hydrazone ligands exhibits molecular ion peaks [M + H]^+^ at m/z 441.1037 and 499.0075 respectively which exactly mimics with the molecular mass and confirm their formation whereas **(3), (4), (5), (6)** metal complexes of hydrazone ligand (**HL**^**1**^) have m/z 974.5887, 973.4405, 978.1348, 979.0014 peaks as a results of [M + H]^+^ ion, respectively that are consistent with the formula mass (supplementary Table S1 and Fig. S1–S6)^46^ and confirmed the complexation of ligand.

### NMR spectra

The NMR (^1^H and ^13^C) spectral analysis of the zinc(II) complexes and their associated ligands (supplementary Figs. S7–S14 and Table S2) were examined in DMSO-*d*_*6*_ and CDCl_3_.

^1^H NMR of the hydrazone ligands **(1–2)** exhibited a signal at 8.55–9.42 ppm as result of -HC = N- group which was shifted at 7.86–9.02 ppm on chelation with Zn(II) metal ion and confirm the bonding of N-atom with metal ion. The hydrazone ligands also have a singlet at 12.17–12.28 ppm as a consequence of –NH group and desertion of this peak on complexation confirmed the keto-enol tautomerism and bonding of enolic oxygen with metal ion^47^. The –OH signal (9.86 ppm) of **HL**^**2**^ hydrazone ligand **(2)** was not disappeared on complexation that means it does take participate in the bonding with metal ion. The slightly shifting in aromatic (9.20–7.35 ppm) and aliphatic (1.39–4.19 ppm) signals of hydrazone ligands also affirmed the chelation of ligands metal ion.

The azomethine carbon of hydrazone ligands **(1–2)** exhibited a signal at 158.65–159.28 ppm in ^13^C NMR spectra and shifting of this signal on complexation at 163.18–164.92 ppm confirmed the complex formation via N-atom of –HC=N– group. The shifting of –C=O– group’s signal of hydrazone ligands from 160.56 to 160.58 ppm authenticate keto-enol tautomerism and complexation through enolic oxygen^48^. The spectral graph of the hydrazone ligands also has some aromatic (109.92–148.63 ppm) and aliphatic signals (14.98–65.22 ppm) which slightly shifted on chelation with metal ion and ensure the complex formation.

Therefore, the obtained data indicates that the hydrazone ligands are bonded with Zn(II) metal in bidentate fashion via O-atom of enolic and N-atom of –HC=N– groups as displayed in Fig. 1.

### IR spectra

The bonding sites in the complexes were ascertained by infrared spectroscopy on correlating the IR bands of ligands **(1–2)** with their associated complexes (supplementary Table S3). The –HC=N– group of the hydrazone ligands have a band at 1598–1597 cm^−1^ and shifting at 1597–1591 cm^−1^ frequency range on chelation with metal ion, confirmed the complexation via N- atom of azomethine. The disappearance of (C=O)_ketonic_ (1656–1647 cm^−1^), –NH (3237–3230 cm^−1^) bands and appearance of (C–O)_enolic_ absorption band (1295–1277 cm^−1^) on complex formation, revealed the keto enol tautomerism and bonding via O-atom of (C–O)_enolic_ group^49^. The hydrazone ligands also exhibited a band at 1049–1042 cm^−1^ because of (N–N) group and shifting of this frequency at 1042–1032 cm^−1^ confirmed the bonding of N-atom of azomethine group with metal atom. The emergence of new bands like M–O, M–N, M–OH_2_ at 539–530, 473–464, 3459–3442 cm^−1^, respectively in complexes confirm that the hydrazone ligands are bonded with central metal atoms through O-atom of enolic group, N-atom of –HC=N– group and O-atom of water molecules^50^. Thus, the obtained IR data is well consistent with NMR results and validate the complex formation.

### Powder XRD (X-ray diffraction) analysis

The powder XRD analysis depicts the average crystallite size (D), nature (amorphous or crystalline) and dislocation density (δ) of the compounds. The obtained pattern states that the complex **(4)** has amorphous nature while its ligand **(1)** shows crystalline nature (supplementary Fig. S15)^51^. The average crystallite size and dislocation density^52^ of the ligand **(1)** were calculated by using the below Eqs. (2–3) and found to be 30.32 nm and 0.00108 nm^−2^, correspondingly:

2 $${\text{Debye}}\;{\text{Scherrer}}\;{\text{equation}},\;{\text{D}}_{{{\text{XRD}}}} = \frac{{{\text{k}}\uplambda }}{{\left(\upbeta \right){\text{cos}\uptheta }}}$$

3 $$\delta = {1}/{\text{D}}^{{2}}_{{{\text{XRD}}}}$$

where K—shape factor (0.95), β—full width at half maximum of the reference diffraction peak, $\uptheta$—diffraction angle (20–80°), λ—wavelength (1.5406 Å).

### SEM (scanning electron microscopy) and EDAX (energy dispersive X-ray spectroscopy) analysis

**Figure 2:**
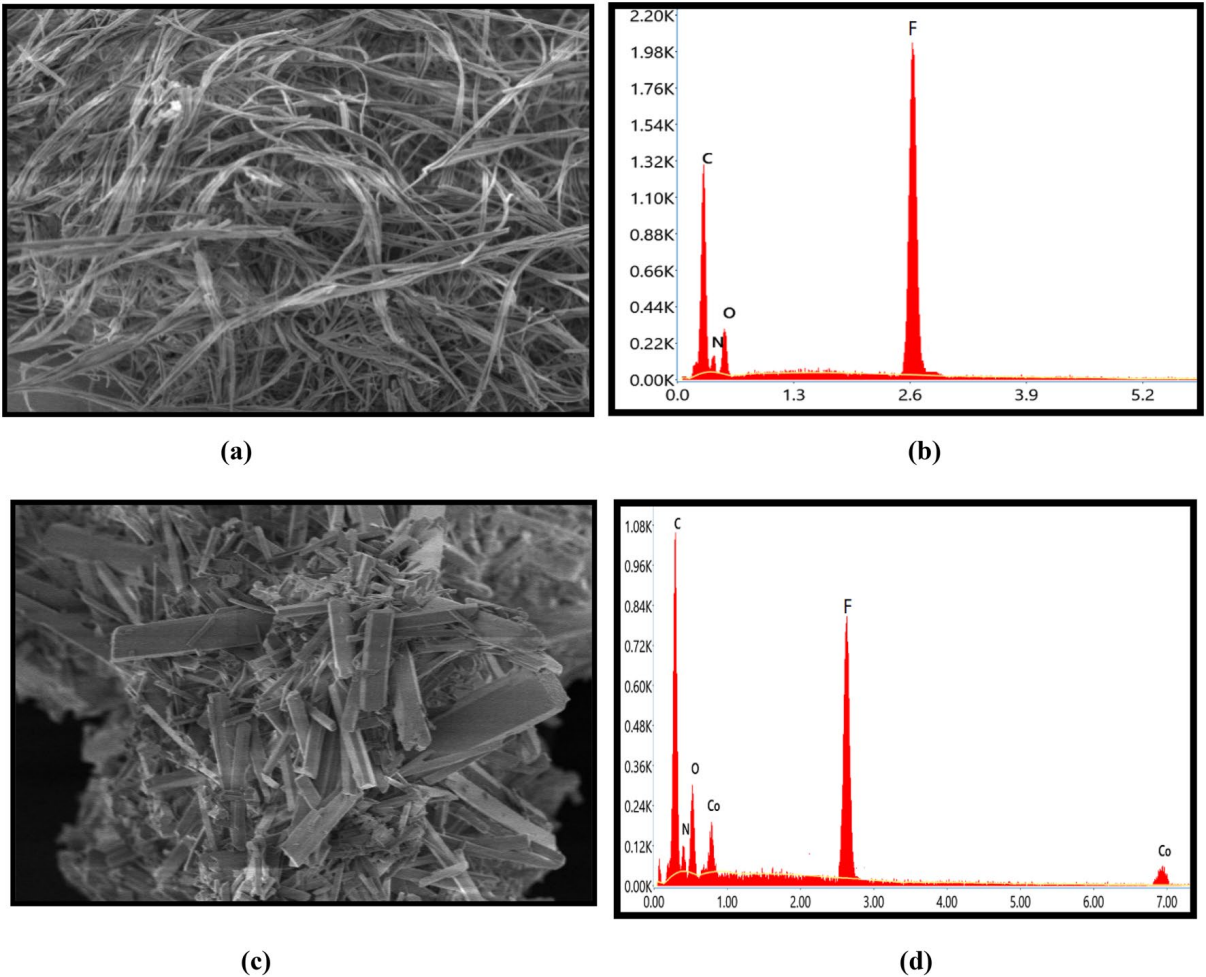
SEM and EDAX images of **HL**^**1**^ ligand **(1)** (**a**, **b**) and its [Co(L^1^)_2_(H_2_O)_2_] complex **(3)** (**c**, **d**).

The surface morphology of the compounds was demonstrated using the SEM technique, which revealed that the hydrazone ligands and complexes have distinct surface morphology. The micrographs of ligand **(1)** and its [Co(L^1^)_2_(H_2_O)_2_] complex **(3)** are represented in Fig. 2 and have thread like morphology for ligand **(1)** while its complex **(3)** has rectangular bar like morphology. The reason behind difference in surface appearance of the ligands and complexes may be as a result of crystal aggregation and chelation of ligand with metal ion. Thus, SEM analysis supported the complex formation because the surface morphology of ligand was changed on complexation^53^.

The EDAX analysis evaluate the elemental compositions of the compounds as represented in Fig. 2. The numerous significant constitutes such as C, N, O, F were reported in micrograph of ligand **(1)** although the complex formation of ligand was clearly endorsed by emergence of Co(II) metal in micrograph of complex **(3)**^54^. So, EDAX data also authenticate the complexation process of ligands.

### UV–Vis spectra and magnetic susceptibility

The stereochemistry of the complexes is also authenticated by UV–Vis spectra and magnetic susceptibility. The ligands **(1–2)** exhibits two absorption bands at 26,020–26,570 and 38,890–38,982 cm^−1^ as a result of n → π* transition of azomethine and π → π* transition of benzene ring, respectively, which disappeared on complex formation and confirm chelation of ligands with metal atoms^55^.

The cobalt(II) complexes have three absorption bands at 10,509–10,538, 17,338–17,786 and 22,795–22,824 cm^−1^ due to the ^4^T_1g_(F) → ^4^T_2g_(F) (υ_1_), ^4^T_1g_(F) → ^4^A_2g_(F) (υ_2_) and ^4^T_1g_(F) → ^4^T_1g_(P) (υ_3_) transitions, respectively (supplementary Table S4 and Fig. S16) which affirmed the octahedral environment in Co(II) complexes. Further, the magnetic moment, Dq, Co(υ_3_/υ_1)_, β%, B and β values at 4.38–4.56 BM, 1184.65–1187.75, 2.165–2.169, 6.4, 908.23–908.36 and 0.936, respectively also confirmed the hexadentate nature of Co(II) complexes. The Ni(II) complexes also presented three bands as a consequences of ^3^A_2g_(F) → ^3^T_2g_(F) (ʋ_1_), ^3^A_2g_(F) → ^3^T_1g_(F) (ʋ_2_) and ^3^A_2g_(F) → ^3^T_1g_(P) (ʋ_3_) transitions at 10,285–10,318, 18,735–18,915 and 22,238–23,985 cm^−1^, respectively, revealing octahedral stereochemistry around nickel(II) metal ions. The octahedral geometry of nickel(II) complexes is also validated by magnetic moment, Dq, Ni(υ_2_/υ_1)_, β%, B and β values at 3.23–3.42 BM, 1028.5–1031.8, 1.821–1.833, 27.6–29.7, 724.33–746.60 and 0.703–0.724, respectively. The octahedral environment around copper(II) metal was confirmed by two absorption bands at 15,556–15,578 and 23,324–23,430 cm^−1^ because of ^2^B_1g_ → ^2^A_1g_ (υ_1_) and ^2^B_1g_ → ^2^E_2g_ (υ_2_) transitions, respectively and magnetic moment value 1.75–1.80 BM. The zinc(II) complexes have diamagnetic nature and d^10^ configurations which indicates one absorption band at 23,584–23,652 cm^−1^ because of LMCT (ligand to metal charge transfer) and demonstrate the octahedral stereochemistry around Zn(II) ion^56^. The magnetic moment, Dq, Co(υ_3_/υ_1)_ or Ni(υ_2_/υ_1)_, β%, B and β values (supplementary Table S4) were calculated by using the equations as mentioned in previously published work^48^. Thus, the obtained UV–Vis data affirmed octahedral geometry of the complexes.

#### TGA

The thermal stability and inside or outside position of the water molecules in the coordination sphere were detected by TGA analysis which was examined in 30–900 °C temperature region. The thermogram of complex **(8)** is represented in supplementary Fig. S17 and have three successive weight loss steps instead of sharp weight loss which is in good consistency with proposed formulae^57^. The first step indicates the weight loss of 3.38% (calcd. 3.30%) as a result of decomposition of water molecules at 175 to 280 °C temperature. Further, the second and third decomposition steps have 47.48% (Calcd. 47.25) and 39.17% (Calcd. 39.02%) weight loss in the 280–535 °C and 535–730 °C temperature region, respectively as a consequences of decomposition of both the ligand moiety one by one, leaving nickel(II) oxide as residue^58^.

Thus, the synthesized complexes followed the three decomposition steps as mentioned below (4–6) and leaves metal oxide as residue, therefore, the obtained results showed stability of complexes upto 175 °C, presence of H_2_O molecules inside of the coordination sphere and complexes non-volatile ability.

4 $$\left[ {{\text{M}}\left( {{\text{L}}^{{1}} - {\text{L}}^{{2}} } \right)_{{2}} \left( {{\text{H}}_{{2}} {\text{O}}} \right)_{{2}} } \right] \to \left[ {{\text{M}}\left( {{\text{L}}^{{1}} - {\text{L}}^{{2}} } \right)_{{2}} } \right]\quad \left[ {{\text{Degradation}}\;{\text{of}}\;{\text{two}}\;{\text{water}}\;{\text{molecules}}\;{\text{at}}\;175 - 280\,{^\circ }{\text{C}}} \right]$$

5 $$\left[ {{\text{M}}\left( {{\text{L}}^{{1}} - {\text{L}}^{{2}} } \right)_{{2}} } \right] \to \left[ {{\text{M}}\left( {{\text{L}}^{{1}} - {\text{L}}^{{2}} } \right)} \right]\quad \left[ {{\text{Degradation}}\;{\text{of}}\;{\text{one}}\;{\text{ligand}}\;{\text{moiety}}\;{\text{nearby}}\;280 - 535\,{^\circ }{\text{C}}} \right]$$

6 $$\begin{aligned} \left[ {{\text{M}}\left( {{\text{L}}^{{1}} - {\text{L}}^{{2}} } \right)} \right] & \to {\text{Metal}}\;{\text{oxide}} + {\text{L}}^{{1}} - {\text{L}}^{{2}} \quad \left[ {{\text{Degradation}}\;{\text{of}}\;{\text{second}}\;{\text{ligand}}\;{\text{moiety}}\;{\text{which}}} \right. \\ & \quad \left. {{\text{leaves}}\;{\text{metal}}\;{\text{oxide}}\;{\text{as}}\;{\text{residue}}\;{\text{above}}\;730\,{^\circ }{\text{C}}} \right] \\ \end{aligned}$$

$${\text{M}} = {\text{cobalt}}\left( {{\text{II}}} \right),{\text{nickel}}\left( {{\text{II}}} \right),{\text{copper}}\left( {{\text{II}}} \right),{\text{zinc}}\left( {{\text{II}}} \right)$$

### ESR spectra

The ESR spectra is very valuable spectroscopy to demonstrate the geometry of copper(II) complexes. The ESR spectra of Cu(II) complex **(5)** is represented in supplementary Fig. S18. The g_∥_ and g_⊥_ values of the copper(II) complex **(5)** were reported at 2.21 and 2.06, respectively which follows g_∥_ > g_⊥_ > 2.0023 trend and shows the existence of unpaired electron in d_x2_-_y2_ orbital, confirmed octahedral geometry of the complex^59^ (Table 1). The g_∥_ value (2.21) of complex **(5)** was found to be less than 2.3 which showed covalent nature of the complex. Further, the g_av_ (2.11) value is determined by the given formula (7):

7 $${\text{g}}_{{{\text{av}}}} = {1}/{3}\left( {{\text{g}}_{\parallel } + {\text{2g}}_{ \bot } } \right)$$

**Table 1:** ESR spectra of copper(II) complexes.

C. no	copper(II) complexes	g_∥_	g_⊥_	g_av_	G
(5)	[Cu(L^1^)_2_(H_2_O)_2_]	2.21	2.06	2.11	3.59
(9)	[Cu(L^2^)_2_(H_2_O)_2_]	2.23	2.07	2.12	3.36

Hathway and billing states that,

If G > 4—no interaction between copper ions, G < 4—some exchange interaction in copper ions^60^.

Therefore, the complex **(5)** has some exchange interaction in copper ions because G (anisotropic geometric parameter = 3.59) is less than 4 which calculated by the given formula (8):

8 $${\text{G}} = \left( {{\text{g}}_{\parallel } - {2}.00{23}} \right)/\left( {{\text{g}}_{ \bot } - {2}.00{23}} \right)$$

### Biological studies

WHO’s Global Tuberculosis Report 2022 states that the TB is a very serious threat for human being in comparison of COVID-19 and HIV/AIDS because it is expanding very rapidly worldwide. The WHO recognized that the Africa and South-East Asia have reported 82% TB deaths while 32% deaths were recorded only in India. So, this alarming data inspired us for the antituberculosis investigations of the compounds **(1–10)** to discover a combating agent for pharmaceutical industries. TB is an infectious disease, therefore, sometime the TB patient faces many other problems caused by microbes and inflammation; therefore, the microbial and inflammation inhibition properties of the compounds **(1–10)** are centre of investigation. The antituberculosis and antimicrobial are evaluated at different concentration and results are represented as MIC values. MIC (minimum inhibitory concentration) is the lowest concentration of a biological activity that hinder the growth of strain. So, MIC value is related to the dilution test because we have recorded the lowest possible concentration in dilution test which hinder the growth of strain and help in calculating the MIC value.

### Antituberculosis activity

**Figure 3:**
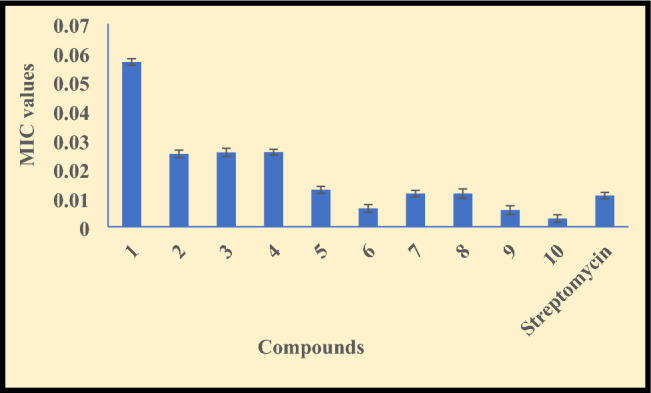
Anti-TB data of the compounds **(1–10)** and standard drug (streptomycin).

The tuberculosis (TB) is a *Mycobacterium tuberculosis* based serious ailment that can affect the lungs, brain, skin, lymph nodes, kidney, spine etc. TB also effects the immune system of the body and the major cause of death if the patient does not acquire the proper treatment. Therefore, in the anticipation of an anti-TB agent, the compounds **(1–10)** were investigated by micro plate alamar blue assay *against M. tuberculosis* H_37_R_v_ strain by taking streptomycin as standard drug. The obtained MIC values are mentioned in Table S5 of supplementary and represented in Fig. 3.

The activity results are as follows:

The compounds **(1–10)** exhibit moderate to good tuberculosis inhibition ability and among the hydrazone ligands, **HL**^**2**^** (2)** is more active for TB strain with 0.0251 ± 0.0013 µmol/mL MIC value as a consequence of attached electron withdrawing groups^61^. Generally, the anti-TB effect of the ligands got enhanced on complexation while some complexes have comparable anti-TB ability with ligands and shows the mentioned activity trend- Zn(II) > Cu(II) > Ni(II) ≃ Co(II) because of decreased polarity on the central metal ion by partially sharing of positive charge with donor groups and pi-electron delocalization within the chelating ring system which is formed during complexation. Therefore, the lipophilic nature of the metal atom was increased with increase in hydrophobic ability and favor the efficient permeation of complexes via lipid layers of the cell wall as mentioned by Tweedy’s chelation theory^62^. The chelation, metal drug synergism and drug transporter nature of the complexes have also supported the increased bioavailability of the complexes^63^.

The complexes **(3–10)** showed the MIC values in the 0.0028 ± 0.0013–0.0257 ± 0.0010 µmol/mL range. The cobalt(II) and nickel(II) complexes **(3, 4)** of ligand **(1)** are least active with 0.0256 ± 0.0014–0.0257 ± 0.0010 µmol/mL MIC value and copper(II) complex **(5)** exhibit moderate anti-TB activity (0.0127 ± 0.0012 µmol/mL); while the copper(II) complex **(9)** and zinc(II) complexes **(6, 10)** are more potent to control the TB deformities with 0.0028 ± 0.0013–0.0063 ± 0.0013 µmol/mL whereas cobalt(II) and nickel(II) complexes **(7, 8)** have equal potency to inhibit the growth of TB (0.0114 ± 0.0011–0.0114 ± 0.0016 µmol/mL). Overall, zinc(II) complex **(10)** is nearly four time potent with MIC value (0.0028 ± 0.0013 µmol/mL) to inhibit TB dysfunction than the streptomycin (0.0107 ± 0.001) because of metallic effect, attached group in the ligand and stabilized aromatic ring that influence the pharmaceutical ability of the compound. So, this research gives a new insight for in vivo investigation of anti-TB drug.

Further, the literature survey exhibit that the synthesized compounds have more anti-tuberculosis effect in comparison of previously reported compounds^62,64^.

### Antimicrobial activity

**Figure 4:**
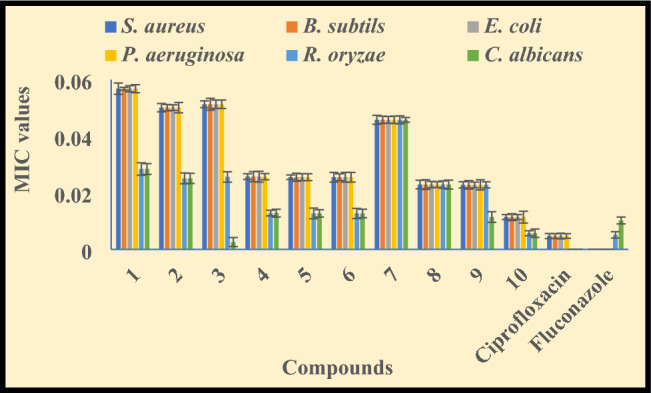
Antimicrobial data of the compounds **(1–10)** and standard drugs (ciprofloxacin and fluconazole).

Tuberculosis is also a microbial disease but microbes mainly effect the lungs while the TB bacteria attack on the other parts of the body like brain, kidney, liver etc. Therefore, the healthcare peoples recommend the antimicrobial medicine to cure the microbial infection during tuberculosis. So, in the view of this, we have examined the antimicrobial potency of the synthesized compounds **(1–10)** via serial dilution assay against fungal (*R. oryzae, C. albicans*) and bacterial (*P. aeruginosa, B. subtilis, S. aureus, E. coli*) strains. The microbial inhibition ability was presented as MIC values (Fig. 4 and supplementary Table S6) and compared with fluconazole and ciprofloxacin (standard drugs).

The activity led to the following outcomes:

We have observed that the synthesized compounds have moderate to good inhibition ability for the microbial deformities while the complexes have more activity in comparison of hydrazone ligands as explained by Overtone’s concept and Tweedy’s chelation theory^21,44^. The **HL**^**2**^ hydrazone ligand **(2)** is most active (MIC value 0.0501 and 0.0251 µmol/mL for bacterial and fungal strains, respectively) among the ligands and the complexes have Zn(II) ≥ Cu(II) > Ni(II) > Co(II) sequence^65,66^.

Against the bacterial strains, the bacterial inhibition of the complexes was enhanced in the MIC value range of 0.0114–0.0513 µmol/mL. The copper(II) **(5)** and zinc(II) **(6)** complexes of **HL**^**1**^ have equal microbial inhibition ability (MIC value 0.0255 µmol/mL) while the nickel(II) and copper(II) complexes exhibit comparable MIC values. Although, the cobalt(II) complexes are least active and zinc(II) complex **(10)** of **HL**^**2**^ ligand has highest potency against all the tested bacterial strains with lowest MIC value 0.0114 µmol/mL^67,68^.

The antifungal activity exhibit that the MIC value of the ligands was enhanced on complexation in 0.0057–0.0256 µmol/mL range. The cobalt(II) complexes have least fungal inhibition ability but the copper(II) **(5)** and zinc(II) **(6)** complexes of **HL**^**1**^ ligand (MIC value 0.0127 µmol/mL) have equal potency to control the fungal deformities. While, the nickel(II) **(8)** and copper(II) **(9)** complexes of **HL**^**2**^ ligand shows equal MIC values for antifungal activity. Although, amid the synthesized complexes, zinc(II) complex **(10) HL**^**2**^ ligand has highest biological response against fungal disease with least MIC value (0.0057 µmol/mL).

Overall, the zinc(II) complex **(10)** exhibit more potency to inhibit the growth of microbial diseases with significant MIC value (0.0057–0.0114 µmol/mL). Thus, it may be used as promising antimicrobial agent to inhibit the microbial causing malformation.

The comprehensive review of the synthesized compounds and previously reported compounds^69–72^ indicates that the compounds **(1–10)** are significantly potent for disease caused by microbes.

### Anti-inflammatory activity

**Figure 5:**
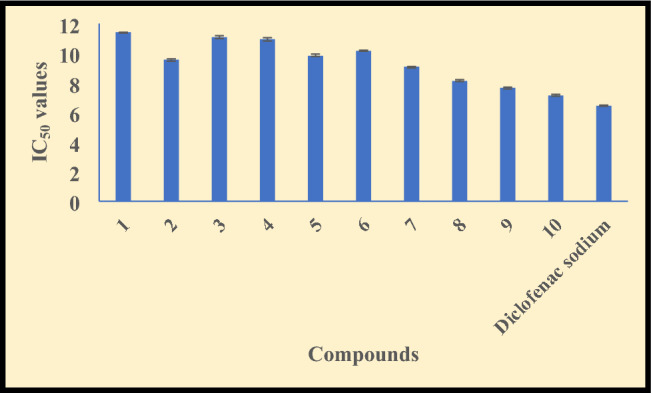
Anti-inflammatory data of the compounds **(1–10)** and standard drug (diclofenac sodium).

The inflammation is a leading reason of many serious ailments such as lungs problem, kidney failure, gastrointestinal disorder, heart failure etc. and also damage the heathy cells or organs during tuberculosis. The injuries, environmental problem, pathogen, radiations, medicinal allergy etc. are the main reasons behind the inflammation. Therefore, to cure the patient from these problems, we require an enriched anti-inflammatory drug which control the inflammation without any side effects. So, we carried out the BSA assay to examine the inflammation inhibition ability of the compounds. The obtained IC_50_ values are mentioned in Table S7 of supplementary and graphically represented in Fig. 5.

The activity indicates the below mentioned results:

The compounds **(1–10)** are significantly active for inflammation and the hydrazone ligands follows the **HL**^**2**^ (9.56 ± 0.10 µM) > **HL**^**1**^ (11.41 ± 0.05 µM) activity trend. The anti-inflammation activity of the hydrazone ligands was enhanced on complex formation in the 7.14 ± 0.05–11.08 ± 0.10 µM range and have the Zn(II) > Cu(II) > Ni(II) > Co(II) activity order because on complexation they inhibit protein denaturation that block the retention of water and preparation of adema that causes adema to be blocked and the inflammation to be reduced^73,74^.

The complexes **(8), (9), (10)** have comparable IC_50_ value (7.14 ± 0.05–08.12 ± 0.04 µM) with the standard drug diclofenac sodium while complex **(10)** has lowest IC_50_ value (7.14 ± 0.05 µM) which is very near with standard drug. Therefore, it may be used in curing the inflammation diseases during tuberculosis.

The synthesized compounds also have more anti-inflammation property than the previously reported compounds^75,76^ as revealed by literature study.

### Structure activity relationship

**Figure 6:**
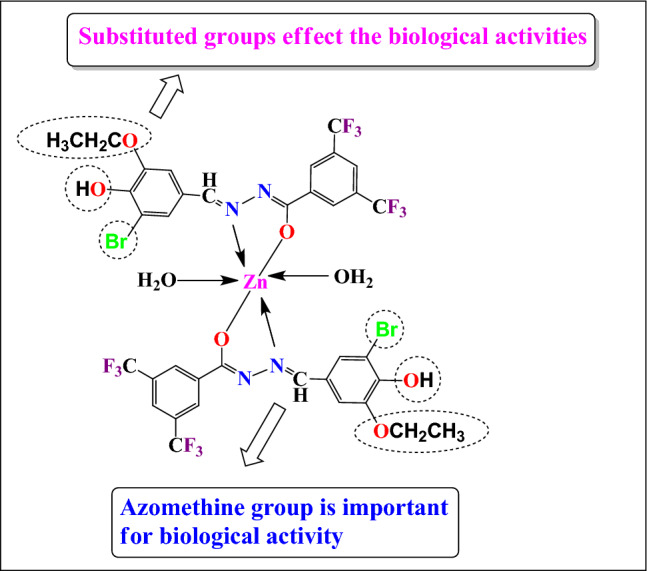
Structure activity relationship of the compounds.

To analyze the consequences of ligand’s bonded groups and metal on the pharmacological activity (Fig. 6), structure activity relationship was assessed. Literature study^23,25,36,42^ revealed that the electron withdrawing groups substantially effect the anti-TB, antimicrobial and anti-inflammatory activities, so, analyzing this, the synthesis of electron donating and electron withdrawing groups-based ligands **(HL**^**1**^**–HL**^**2**^**)** were carried out for comparing their biological activities. The in vitro biological investigation revealed that the **HL**^**2**^ ligand **(2)** is more effective to control the infectious diseases in comparison of the **HL**^**1**^ ligand **(1)** because of electron withdrawing groups effects of the moiety. Further, the biological activities results demonstrated that the metal complexes have more pharmacological efficacy than the ligands as a consequence of chelation, lipophilicity, distribution of charge binding ability, DNA cleavage property etc. Although, zinc(II) complex **(10)** was reported as most potent among the complexes which may be due to metallic effect, chelating property, stability of the complex etc.

Lipophilicity is a significant physicochemical characteristic of biologically active compounds that influences their activity by playing a key role in the transport of compounds across biological membranes and in the creation of the ligand-receptor complex. The lipophilic nature of an active compound, defined as the ability of a compound to pass through hydrophobic barriers to reach the site of action from the delivery point. The tweedy chelation theory shows that the complexation increases the lipophilicity (supplementary Table S8) as well as hydrophobicity of the compounds which increased their biological activity.

### Computational studies

The discovery of new effective therapeutic drug with minimal disadvantages is a very difficult task in medicinal chemistry, so, to overcome the various barriers in dug design and to support the experimental results, researchers utilize the computational studies which provides significant parameters such as binding energy, binding interactions, energy gap etc. In the current research, the **HL**^**2**^ ligand **(2)** and its complexes **(7–10)** are highly active for the performed biological activities, therefore, the computational investigations of these compounds are centre of research to resolve the numerous challenges of pharmaceutical industries.

### Molecular docking

**Figure 7:**
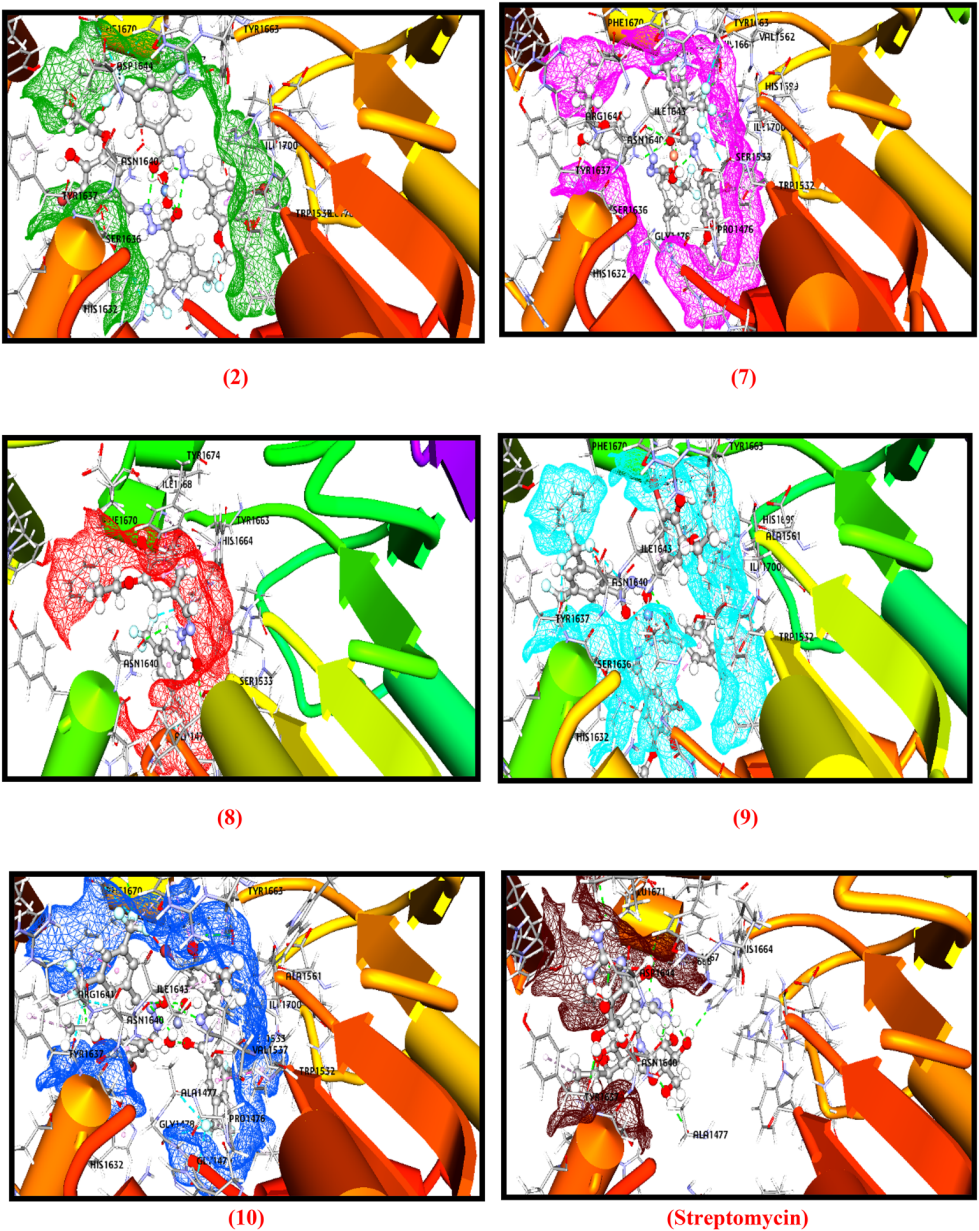
Molecular docking interactions of the compounds **(2, 7–10)** and streptomycin against 5V3Y protein.

The molecular docking analysis is a very valuable technique in the field of medicinal chemistry because it provides the binding energy and binding interaction which tell us how the compound interacts with the proteins. The compounds' strong affinity for the binding pocket was shown by the negative values of free energy in the grid box produced by the docking study of the compounds **(2, 7–10)** against 5V3Y (Mtb Pks13 Thioesterase Domain) protein receptor that based on *M. tuberculosis*. Numerous interactions were present in each of the compound's binding conformations against the active binding pocket. The tested compounds were shows comparable binding ability against the active site of the protein (Fig. 7 and supplementary Table S9).

The compounds (**2), (7), (8), (9), (10)** shows the docking score − 137.228, − 123.195, − 132.8095, − 159.956, − 208.926 kcal/mol, respectively while the docking score of standard drug (streptomycin) was found to be − 160.616 kcal/mol. The compound **(9)** has comparable docking score with standard drug while zinc(II) complex **(10)** showed a better docking score of − 208.926 kcal/mol in the active site of the 5V3Y (Mtb Pks13 thioesterase domain) protein in comparison of other tested compounds. The tested compounds indicate various kind of interactions with active site of protein like conventional hydrogen, carbon hydrogen, pi-pi, alkyl, pi-alkyl, halogen bonds etc. Amid the tested compounds, complex **(10)** has created a variety of interactions with various amino acids, including Gln1633, Asn1640, Tyr1663, Tyr1637, Trp1532, His1632, Tyr1663, Phe1670, Tyr1674, Val1483, Arg1641, Ile1643, Typ1532, His1632, Tyr1637, Phe1670, Tyr1674, Ile1700, Gly1479, Tyr1637, Asn1640, Phe1670 (supplementary Table S9) which highly stabilize the complex and support the biological potency of the complex^24,29^.

So, the obtained results clearly state that the complex **(10)** has more potency against tuberculosis and behave as best 5V3Y inhibitors because it shows lowest binding score and good binding interactions, hence, it may be utilized in the place of standard drug for TB and its associated deformities.

### DFT analysis

**Figure 8:**
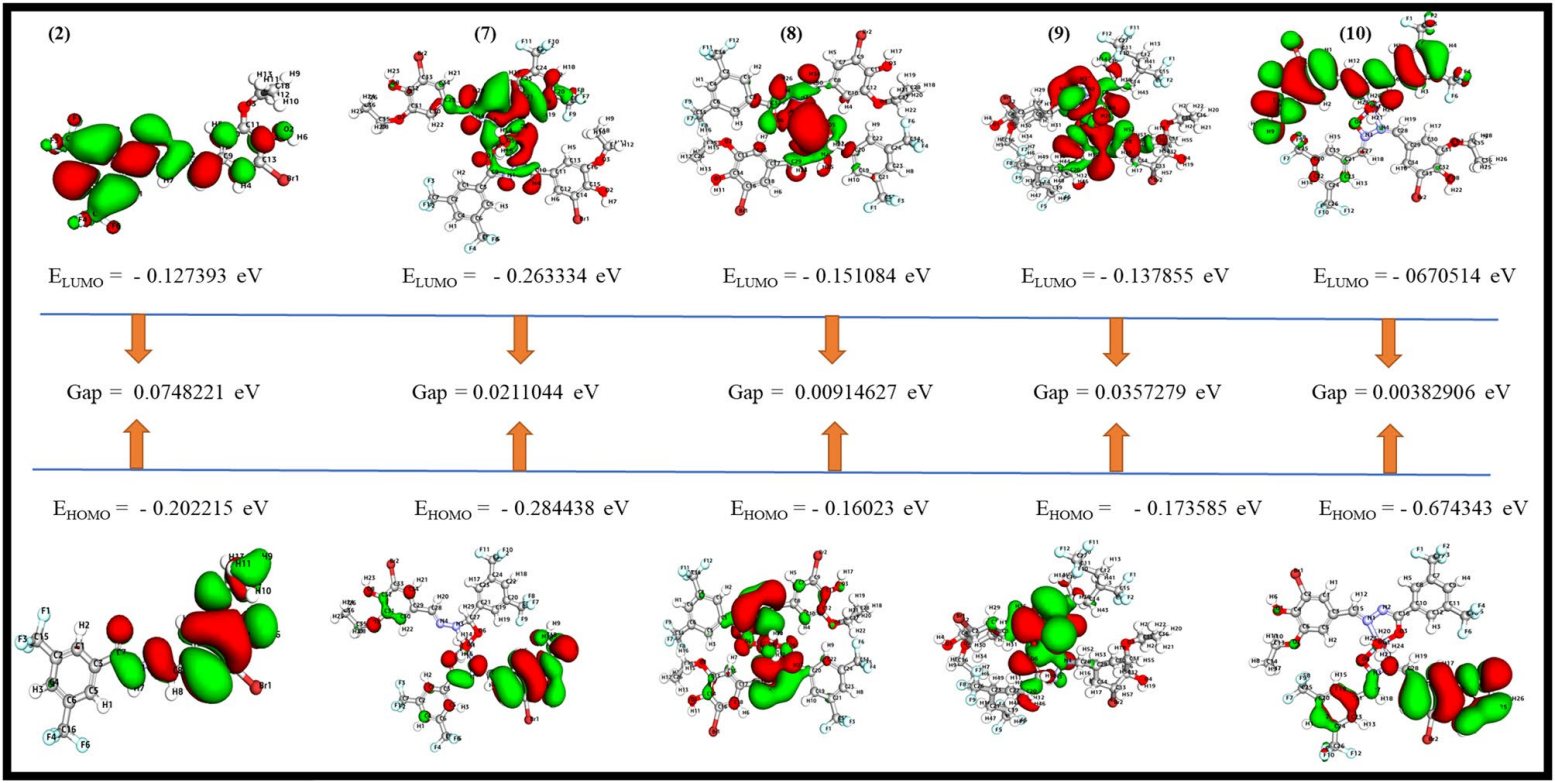
HOMO and LUMO plots of the compounds **(2, 7–10)**.

The primary goal of DFT analysis is to find out a biological active drug among the tested compounds utilizing quantum mechanical principles and their resemblance. The stability of the complexes may be determined by using bond length, where the more stable compound is associated with a shorter bond length. Complex **(8)** molecular structure have shorter bond lengths than ligand **(2)** (highest bond length) and complexes **(7, 9, 10)** (supplementary Table S10 and Fig. 8) which justified the stability of complex **(8)**. The molecular orbitals of all the compounds are researched and the acquired data such as HOMO, LUMO and energy gaps are gathered. All investigated compounds HOMO energy molecular orbitals indicates that the complex **(10)** has the most permanent HOMO molecular orbitals while complex **(8)** has the most reactive HOMO molecular orbitals. Moreover, complex **(10)** has the most stable LUMO molecular orbitals while ligand **(2)** has the most reactive LUMO molecular orbitals. The HOMO and LUMO energy difference is used to compute the energy gap (E.g.) (E_HOMO_-E_LUMO_) (37). The energetic stability and chemically reactive properties of a compound are revealed by its molecular orbitals. The energy gap of complex **(10)** was found to be 0.00382906 eV, demonstrating the complex chemical reactivity, biological characteristics and polarizability. HOMO, LUMO, MESP Isovalues and Color Scale of the compounds **(2, 7–10)** are shown in Table 2**.**

**Table 2:** HOMO, LUMO, MESP isovalues and color scale of the compounds **(2, 7–10).**

Compounds	Isovalues	Spectrum (Color range)		Histogram scale
		Min	Max	
HL^2^	3.000000e−02	0.0000	367.7	
[Co(L^2^)_2_(H_2_O)_2_]	3.000000e−02	0.0000	632.1	
[Ni(L^2^)_2_(H_2_O)_2_]	3.000000e−02	0.0000	1104.490	
[Cu(L^2^)_2_(H_2_O)_2_]	3.000000e−02	0.0000	619.9620	
[Zn(L^2^)_2_(H_2_O)_2_]	3.000000e−02	0.0000	1375.35	

Certain important quantum chemical properties including dipole moment, electronegativity, chemical potential etc. were calculated using the values of HOMO and LUMO as shown in supplementary Table S10. Complex **(7)** has the highest dipole moment value in comparison of other compounds, indicating that it has a significant asymmetry in the distribution of electric charges. As a result, it may be particularly vulnerable to changes in molecular structure and electronic properties when exposed to an unfamiliar electric field. On the other hand, ligand **(2)** structure has highest hardness value than the other compounds (Table S10 of supplementary), suggesting that electrons cannot be easily withdrawn from this compound while other compounds are excellent options for transferring electrons to another acceptor molecule. According to Pearson's HSAB hypothesis, if both compounds are soft or hard then it exhibits good interaction among them^38^. Ligand **(2)** is regarded as hard due to its high global hardness (0.03741105 eV) or low global softness (26.73007039364 eV) values^39^. Therefore, we can conclude that the complexes **(7–10)** have a lot of significant factors which favor their more biological activity and have greater electron donating ability of HOMO electron in electron exchange reactions. Overall, complex **(10)** is a valuable compound to act as chelating agent in biological system as a result of its most permanent HOMO molecular orbitals, lowest energy gap, stable LUMO orbital etc.

### MESP calculations

**Figure 9:**
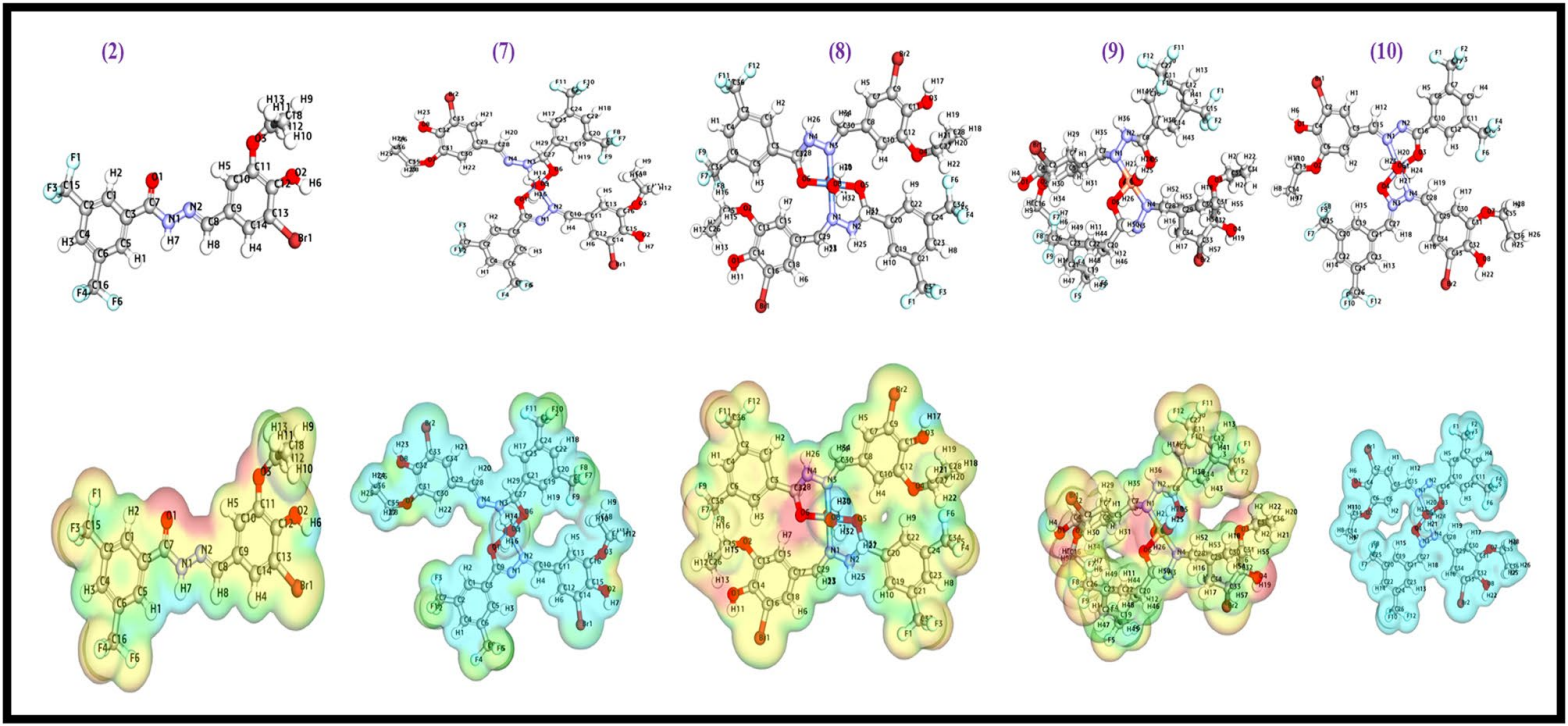
Optimized structures and MESP map of the compounds **(2, 7–10)** with blue color-coding region (signifying electron deficient region) and red color-coding region (signifying electron rich region).

The numerous meaningful statistics like nucleophilic reactions, hydrogen bonding interactions, molecule's structural characteristics and electrophilic attack sites are described through electrostatic potential^48^. The significance of MESP graph exhibits size, negative area, color coding scheme's form, neutral electrostatic potential, positive area and neutral electrostatic potential areas^77^. Figure 9 and supplementary Table S11–S12 shows the MESP results of the compounds **(2, 7–10)**. HOMO, LUMO, MESP isovalues and color scale of the compounds **(2, 7–10)** are shown in Table 2.

In case of ligand **(2)**, H1, H2, H3, H4, H6 and H7 atoms were covered by most positive electrostatic potential whereas only O1 and O3 atoms were covered by most electronegative potential. Interestingly, for complex **(8),** H7, H13 and N1 atoms were located under positive electrostatic potential while F6, F9, H2, H6, H8, H10, H17, H23, H25, H27, H30, H32, N1 and N2 atoms were located under most electronegative potential. Complex **(9)** shows most positive electrostatic potential at C7, C16, H9, H19, H31, O3, O4 and N1 atoms while most electronegative potential at F5, H4, and H25 atoms. The complexes **(7)** and **(10)** shows most positive electrostatic potential throughout all the atoms^78^. So, the MESP calculations well corroborate the obtained in vitro results by demonstrating that the biological efficiency of the ligand **(2)** was enhanced on complex formation.

### ADMET analysis

Water solubility, plasma protein binding, cytochrome P450 2D6 inhibition, cytochrome P450 2D6 inhibition etc. are some of the valuable parameters which ascertained by performing the ADMET analysis^25^. None of the tested compounds **(2, 7–10)** were projected to be able to inhibit cytochrome P450 2D6 like the majority of chemotherapeutic medications. Ligand **(2)** was predicted to have a high plasma protein binding rate which could help with its transportation and release in the blood, a property that the standard medication does not exhibit. Additionally, only complexes **(7)** and **(9)** show hepatotoxicity, and even then, it is only of very low significance (supplementary Table S13–S14). Additionally, the “TOPKAT” module from Discovery Studio was used to forecast the toxicity of the compounds **(2, 7–10)**. Positive responses of complex **(7)** and **(9)** against skin irritancy, ames mutagenicity, carcinogenicity, ocular irritancy and skin sensitization were observed^44^. Supplementary Table S13 provides a summary of expected toxicity parameters, including the developmental toxicity potential, oral LD_50,_ rat inhalational LD_50_, fathead minnow LC_50_, aerobic biodegradability and rat maximum tolerated dose. The compounds have also been examined for carcinogenicity in rats (male and female) and mice (male and female). Complex **(10)** was not exhibiting carcinogenicity in any of the four parameters, although ligand **(1)** exhibited carcinogenic properties. The ames test determines the mutagenic property of compounds, whether they are able to cause mutations or not. All the tested compounds showed mutagenic properties in comparison of standard drug. The compounds **(2, 7–10)** did not show skin irritation while the other compounds along with standard showed skin irritation. The chronic LOAEL test shows the lowest doses that can cause toxic effects in the body which showed values in range with all tested compounds. In comparison of other compounds and streptomycin, the compounds **(2, 7–10)** had the greatest positive outcomes. Hence, from the ADMET analysis, we can reveal that the compounds have drug like properties and does not have any disadvantages on human health when these used as medicine in health care.

## Conclusion

Herein, the synthesis of two hydrazone ligands and their eight transition metal complexes were carried out. The synthesized compounds **(1–10)** were well characterized by numerous spectral and physical investigations. Powder XRD indicate that the complexes are amorphous in nature whereas SEM analysis demonstrated that the compounds have different surface morphologies although the thermal stability of the complexes upto 175 °C was affirmed by thermogravimetric analysis. The spectral studies reveled that the complexes have octahedral geometry. Further, the micro plate alamar blue, serial dilution and BSA assays were conducted to detect the anti-TB, antimicrobial and anti-inflammatory efficacies of the compounds, respectively. The biological screening suggested that the complexes have higher biological efficacy in comparison of hydrazone ligands and follow the Zn(II) ≥ Cu(II) > Ni(II) ≥ Co(II) activity trend. The zinc(II) complex **(10)** was reported as most active for the anti-TB, antimicrobial and anti-inflammatory activities among the compounds with significant 0.0028 ± 0.0013, 0.0057–0.0114 µmol/mL and 7.14 ± 0.05 µM MIC and IC_50_ values, respectively. Molecular docking study against 5V3Y protein exhibit that the zinc(II) complex **(10)** has lowest binding energy (− 208.926 kcal/mol) and various stabilized interaction in comparison of standard drug and other tested compounds, so, complex **(10)** has higher potency to control TB deformities. DFT study shows that the complexes **(7–10)** have more biological efficacy in comparison of ligand and the bioactivity of the complex **(10)** was justified by lowest energy gap, stable LUMO orbital etc. MESP calculations demonstrated that the biological efficiency of the ligand **(2)** was enhanced on complex formation. ADMET analysis revealed that the compounds have significant positive outcomes in comparison of standard drug, therefore, these compounds do not have any disadvantages on medication. Hence, this research demonstrated the experimental and theoretical importance of the hydrazone ligands and their transition metal complexes by providing a significant biological drug that gives new insight in the field of medicinal industries for in vivo investigation and it may be utilized as significant therapeutic agent in place of standard drug.

### Supplementary Information

Supplementary Information.

## Data Availability

The data used to support the findings of this study are included in the article and supplementary material. The datasets generated and/or analysed during the current study are available in the Worldwide Protein Data Bank (wwPDB) repository, [https://doi.org/10.2210/pdb5V3Y/pdb]. In addition, the other information can be made available by the corresponding author upon reasonable request as long as the request does not compromise intellectual property interests.
