# Supplementary material for: Investigation of antituberculosis, antimicrobial, anti-inflammatory efficacies of newly synthesized transition metal(II) complexes of hydrazone ligands: structural elucidation and theoretical studies
Source: Sci Rep. 2023 Sep 23;13:15906. doi: 10.1038/s41598-023-42180-4 (PMC10517985; doi:10.1038/s41598-023-42180-4)
Supplement: Supplementary file 1 — Supplementary Information. [file 41598_2023_42180_MOESM1_ESM.docx]

**Investigation of antituberculosis, antimicrobial, anti-inflammatory efficacies of newly synthesized transition metal(II) complexes of hydrazone ligands: structural elucidation and theoretical studies**

Binesh Kumar^1^, Jai Devi^*1^, Amit Dubey^2,3^, Aisha Tufail^2^, Bharti Taxak^1^

^1^Department of Chemistry, Guru Jambheshwar University of Science and Technology, Hisar-125001, Haryana, India

^2^Computational Chemistry and Drug Discovery Division, Quanta Calculus, Greater Noida-201310, Uttar Pradesh, India

^3^Department of Pharmacology, Saveetha Dental College and Hospital, Saveetha Institute of Medical and Technical Sciences, Chennai-600077, Tamil Nadu, India

**Email id** - [bineshchemistry131@gmail.com](mailto:bineshchemistry131@gmail.com), [jaya.gju@gmail.com](mailto:jaya.gju@gmail.com), [ameetbioinfo@gmail.com](mailto:ameetbioinfo@gmail.com), [aishatufailansari@gmail.com](mailto:aishatufailansari@gmail.com), [bhartitaxak007@gmail.com](mailto:bhartitaxak007@gmail.com)

^*^Corresponding author - Jai Devi, ^1^Department of Chemistry, Guru Jambheshwar University of Science and Technology, Hisar-125001, Haryana, India

**Email id**- [jaya.gju@gmail.com](mailto:jaya.gju@gmail.com)

**Institutional Email id**- [jaidevi2005@gjust.org](mailto:jaidevi2005@gjust.org)

Tel.: +911662263152, Fax: +911662276240, Phone No.: +91-9416789379 (M)


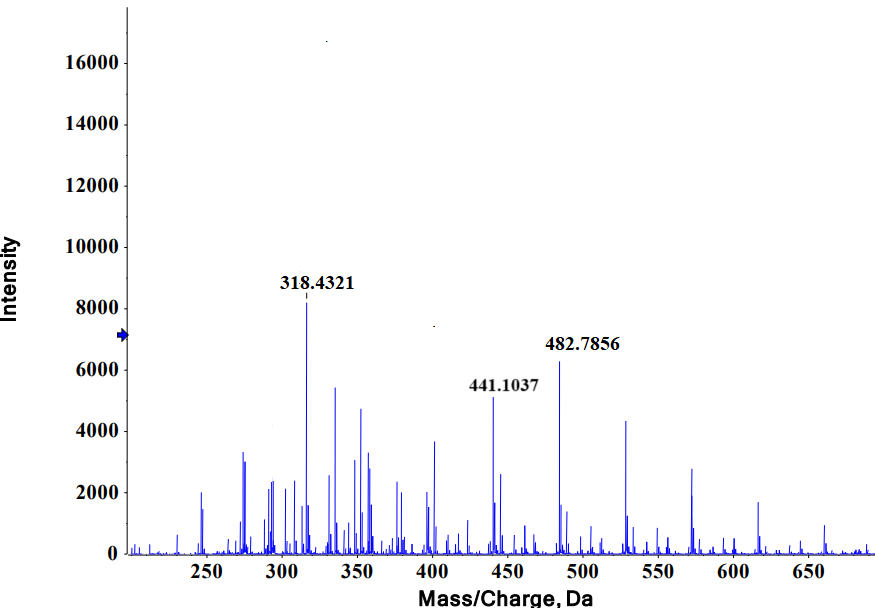


**Figure S1.** Mass spectrum of **HL^1^** ligand **(1).**


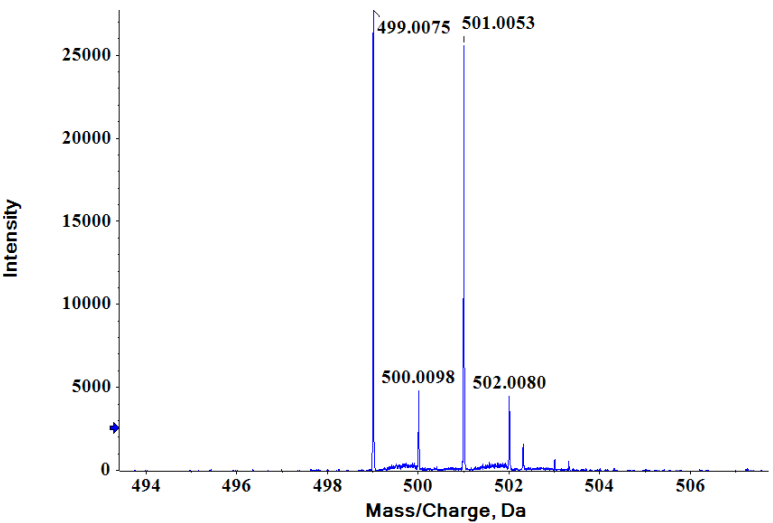


**Figure S2.** Mass spectrum of **HL^2^** ligand **(2).**


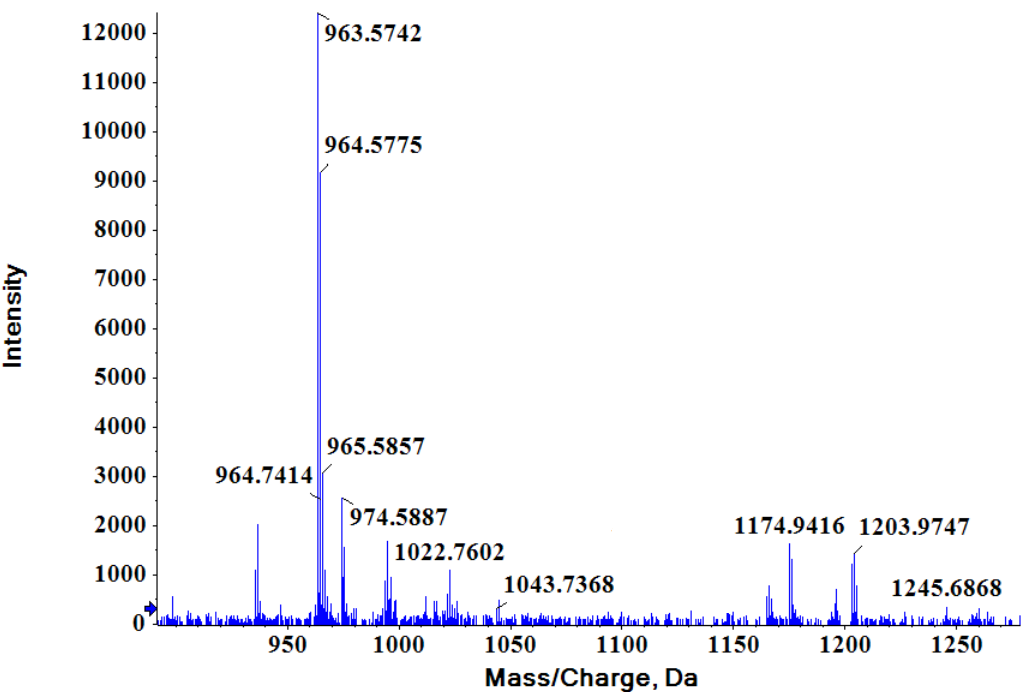


**Figure S3.** Mass spectrum of [Co(L^1^)_2_(H_2_O)_2_] complex **(3).**


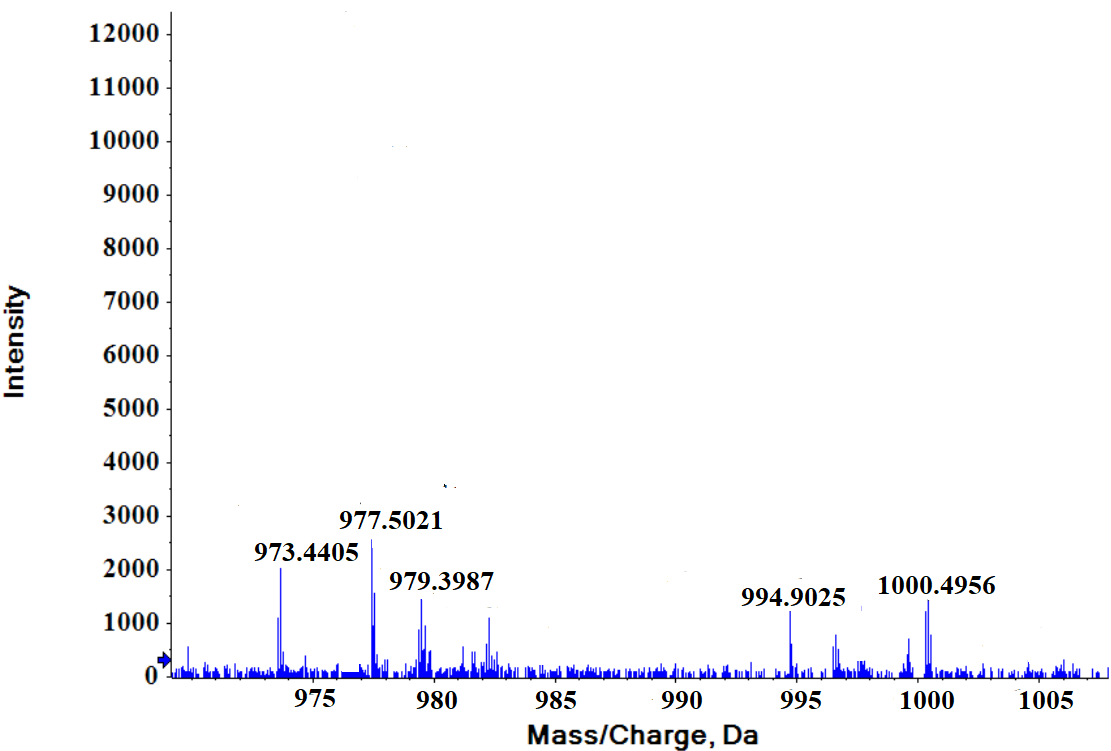


**Figure S4.** Mass spectrum of [Ni(L^1^)_2_(H_2_O)_2_] complex **(4).**


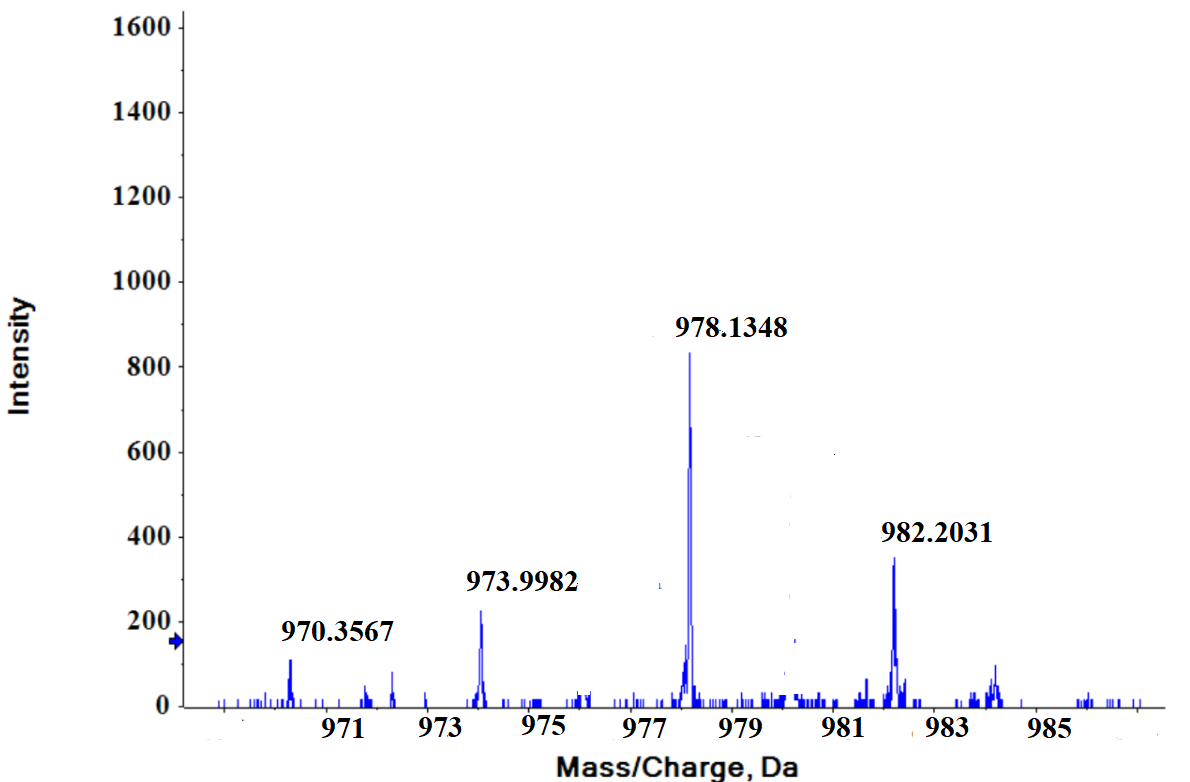


**Figure S5.** Mass spectrum of [Cu(L^1^)_2_(H_2_O)_2_] complex **(5).**

**
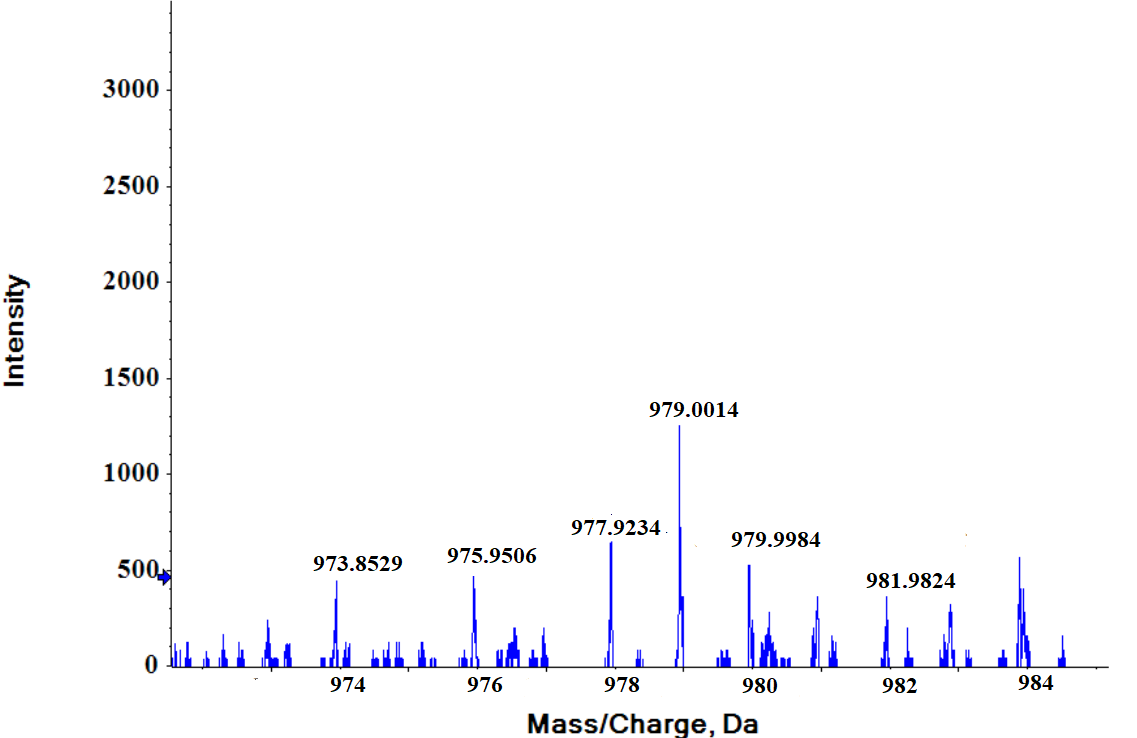
**

**Figure S6.** Mass spectrum of [Zn(L^1^)_2_(H_2_O)_2_] complex **(6).**


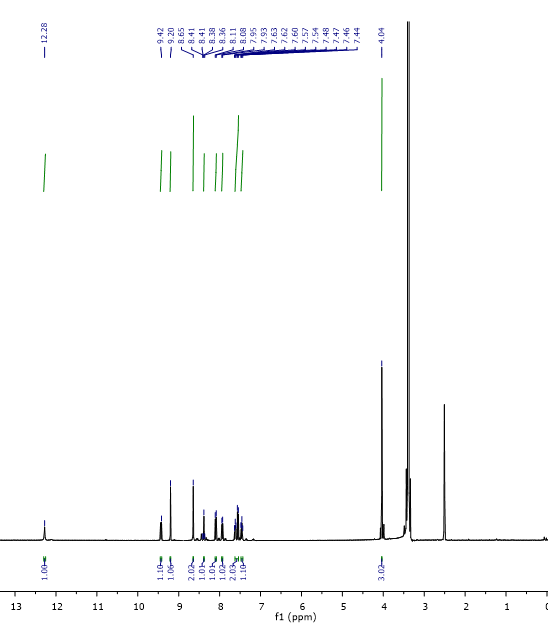


**Figure S7.** ^1^H NMR spectrum of **HL^1^** ligand **(1).**


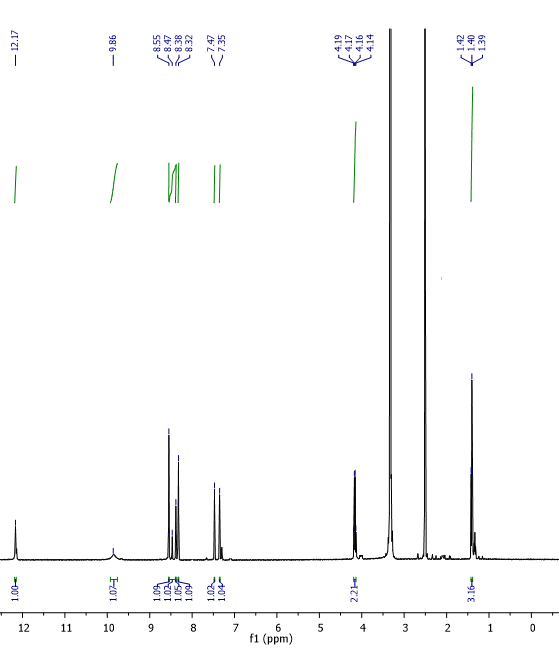


**Figure S8.** ^1^H NMR spectrum of **HL^2^** ligand **(2).**

**
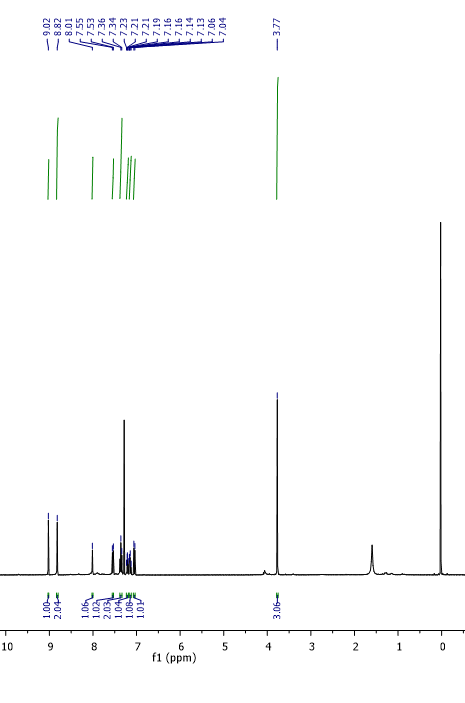
**

**Figure S9.** ^1^H NMR spectrum of [Zn(L^1^)_2_(H_2_O)_2_] complex **(6).**


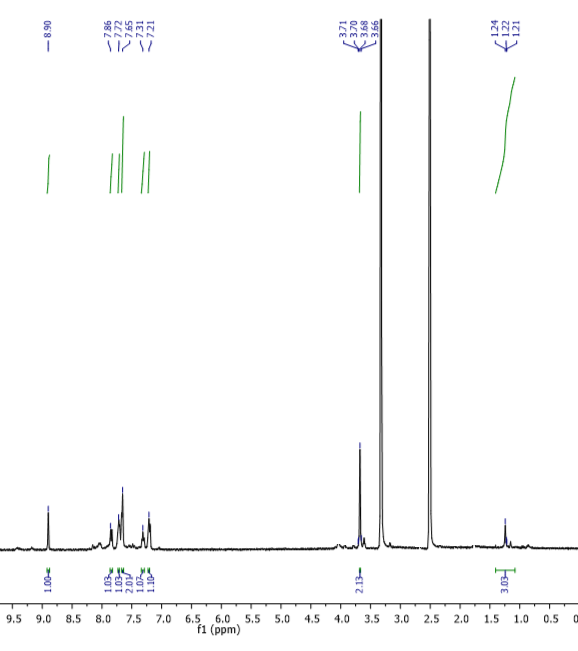


**Figure S10.** ^1^H NMR spectrum of [Zn(L^2^)_2_(H_2_O)_2_] complex **(10).**


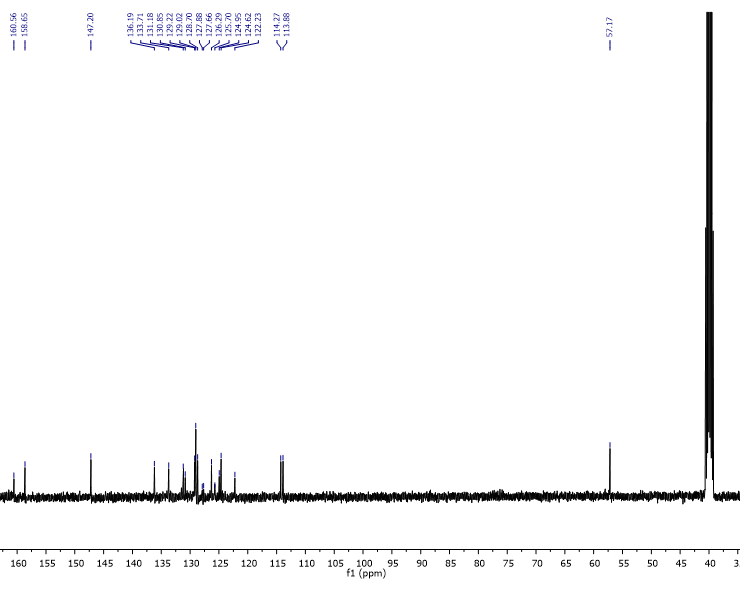


**Figure S11.** ^13^C NMR spectrum of **HL^1^** ligand **(1).**


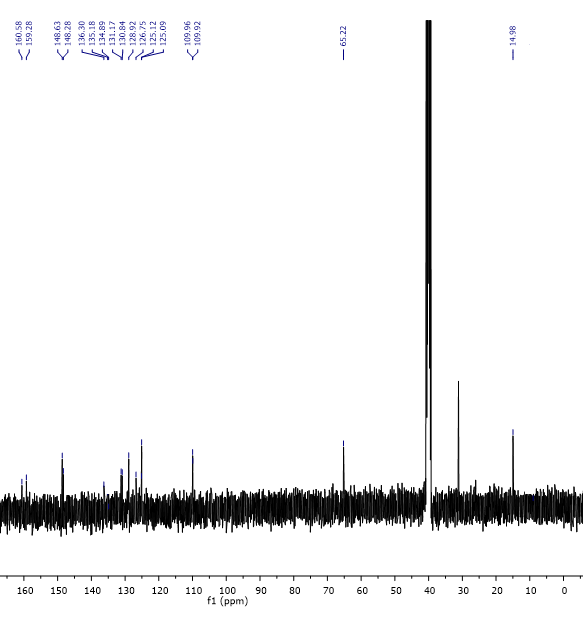


**Figure S12.** ^13^C NMR spectrum of **HL^2^** ligand **(2).**


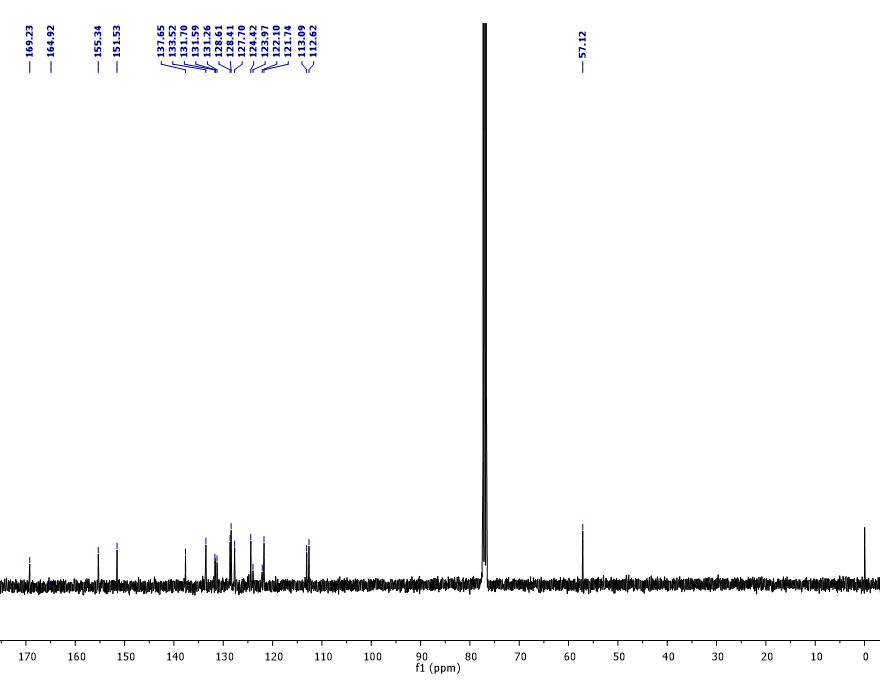


**Figure S13.** ^13^C NMR spectrum of [Zn(L^1^)_2_(H_2_O)_2_] complex **(6).**


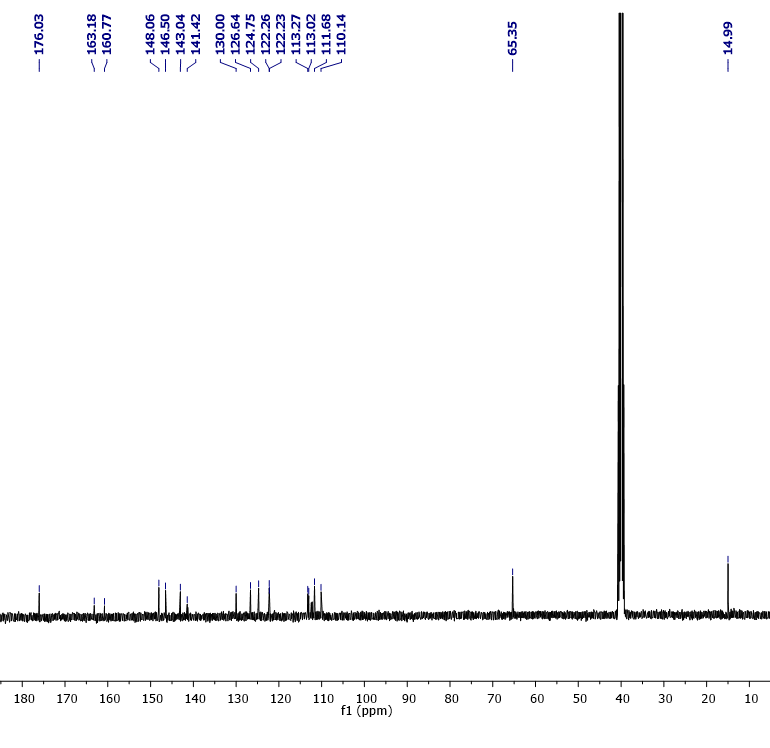


**Figure S14.** ^13^C NMR spectrum of [Zn(L^2^)_2_(H_2_O)_2_] complex **(10).**


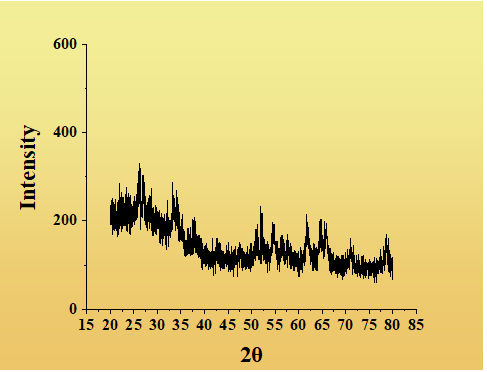

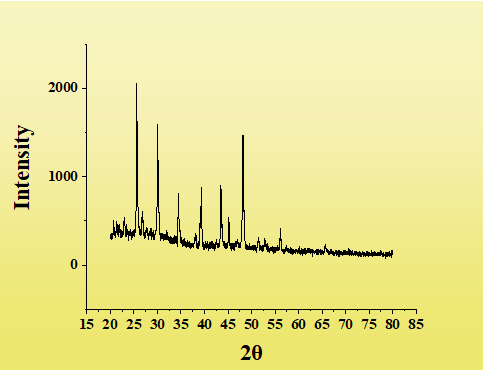


1. (b)

**Figure S15.** Powder XRD of (a) **HL^1^** ligand **(1)** (b) [Ni(L^1^)_2_(H_2_O)_2_] complex **(4).**


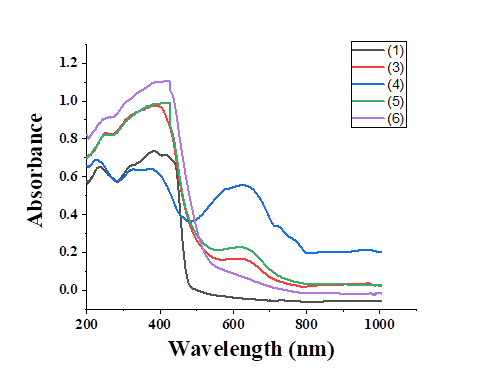

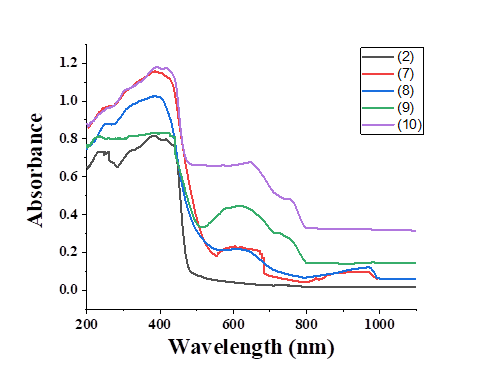


(a) (b)

**Figure S16.** Solid-state UV-Vis spectra of (a) **HL^1^** ligand **(1)** and its complexes (b) **HL^2^** ligand **(2)** and its complexes.

**
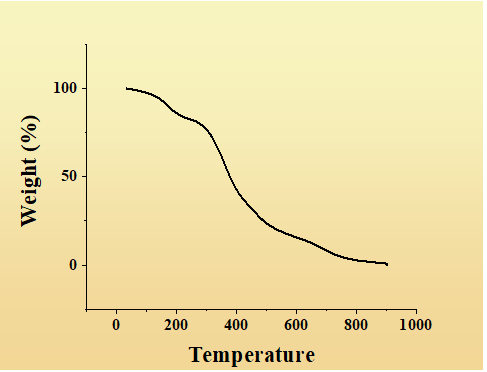
**

**Figure S17.** Thermogravimetric analysis of [Ni(L^2^)_2_(H_2_O)_2_] complex **(8)**.


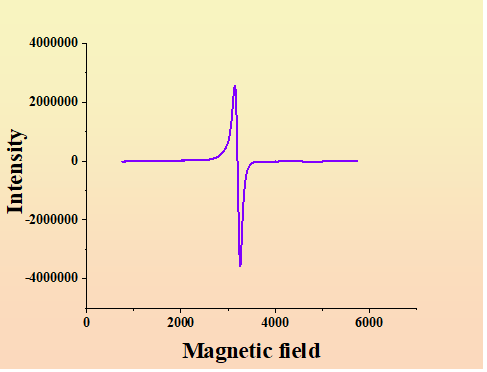


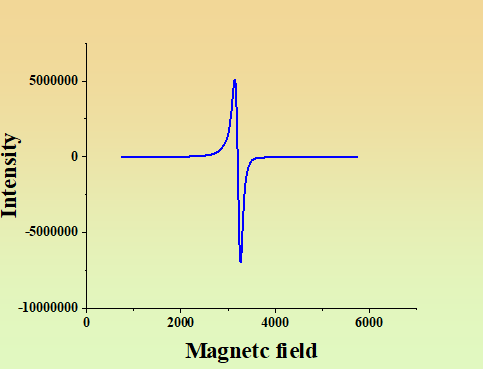


1. (b)

**Figure** **S18.** ESR spectra of [Cu(L^1^)_2_(H_2_O)_2_] complex **(5)** at (a) room temperature (b) nitrogen temperature

**Table S1.** Physical data of the compounds **(1-10).**

| Sl. No. | Compounds | Mol. Formula | Yield (%) | Color | M.P. (˚C) | Conductivity (ohm^-1^ cm^2^ mol^-1^) | Analysis, % found (Calcd.) | | | | *m/z*  (M+H)^+^ |
| --- | --- | --- | --- | --- | --- | --- | --- | --- | --- | --- | --- |
|  |  |  |  |  |  |  | C | H | N | M |  |
| 1 | HL^1^ | C_21_H_14_F_6_N_2_O_2_ | 82 | White | 110-113 | 10 | 57.28  (57.29) | 3.20  (3.21) | 6.36  (6.37) | - | 440.0959  (441.1037) |
| 2 | HL^2^ | C_18_H_13_BrF_6_N_2_O_3_ | 84 | White | 108-110 | 11 | 43.31  (43.32) | 2.62  (2.63) | 5.61  (5.62) | - | 497.9997  (499.0075) |
| 3 | [Co(L^1^)_2_(H_2_O)_2_] | C_42_H_30_F_12_N_4_O_6_Co | 77 | Green | 221-223 | 14 | 51.81  (51.82) | 3.11  (3.13) | 5.75  (5.78) | 6.05  (6.06) | 973.5809  (974.5887) |
| 4 | [Ni(L^1^)_2_(H_2_O)_2_] | C_42_H_30_F_12_N_4_O_6_Ni | 75 | Brown | 223-225 | 18 | 51.82  (51.83) | 3.11  (3.12) | 5.76  (5.77) | 6.03  (6.05) | 972.4327  (973.4405) |
| 5 | [Cu(L^1^)_2_(H_2_O)_2_] | C_42_H_30_F_12_N_4_O_6_Cu | 79 | Dark green | 228-230 | 20 | 51.57  (51.60) | 3.09  (3.11) | 5.73  (5.75) | 6.50  (6.52) | 977.1270  (978.1348) |
| 6 | [Zn(L^1^)_2_(H_2_O)_2_] | C_42_H_30_F_12_N_4_O_6_Zn | 80 | Yellow | 210-212 | 17 | 51.47  (51.49) | 3.09  (3.12) | 5.72  (5.73) | 6.67  (6.69) | 977.9936  (979.0014) |
| 7 | [Co(L^2^)_2_(H_2_O)_2_] | C_36_H_28_Br_2_F_12_N_4_O_8_Co | 79 | Brown | 215-217 | 17 | 39.62  (39.63) | 2.59  (2.60) | 5.13  (5.15) | 5.40  (5.42) | 1090.9394  (1091.9472) |
| 8 | [Ni(L^2^)2(H_2_O)_2_] | C_36_H_28_Br_2_F_12_N_4_O_8_Ni | 78 | Red | 214-216 | 18 | 39.63  (39.65) | 2.59  (2.61) | 5.13  (5.15) | 5.38  (5.41) | 1089.9415  (1090.9493) |
| 9 | [Cu(L^2^)_2_(H_2_O)_2_] | C_36_H_28_Br_2_F_12_N_4_O_8_Cu | 73 | Reddish  brown | 218-220 | 16 | 39.45  (39.46) | 2.58  (2.60) | 5.11  (5.14) | 5.80  (5.82) | 1094.9358  (1095.9436) |
| 10 | [Zn(L^2^)_2_(H_2_O)_2_] | C_36_H_28_Br_2_F_12_N_4_O_8_Zn | 72 | Yellow | 219-221 | 19 | 39.39  (39.40) | 2.57  (2.60) | 5.10  (5.12) | 5.96  (5.98) | 1095.9353  (1096.9431) |

**Table S2.** NMR data of the ligands (1-2) and their Zn(II) metal complexes.

| C. No. | Compounds | ^1^H NMR (δ in ppm) | ^13^C NMR (δ in ppm) |
| --- | --- | --- | --- |
| 1 | HL^1^ | 12.28 (s, 1H, -NH), 9.42 (s, 1H, -HC=N-), 9.20 (s, 1H, Ar-H), 8.65 (s, 2H, Ar-H), 8.41-8.36 (dd, *J* = 7.1, 1.6 Hz, 1H, Ar-H), 8.11-8.08 (d, *J* = 7.1 Hz, 1H, Ar-H), 7.95-7.93 (d, *J* = 7.9 Hz, 1H, Ar-H), 7.63-7.54 (m, 2H, Ar-H), 7.48-7.44 (dd, *J* = 7.4, 1.4 Hz, 1H, Ar-H), 4.04 (s, 3H, -OCH_3_). | 160.56 (-C=O-), 158.65 (-HC=N-), 147.20, 136.19, 133.71, 131.18, 130.85, 129.22, 129.02, 128.70, 127.88, 127.66, 126.29, 125.70, 124.95, 124.62, 122.23, 114.27, 113.88, 57.17 (-OCH_3_). |
| 2 | HL^2^ | 12.17 (s, 1H, NH), 9.86 (s, 1H, OH), 8.55 (s, 1H, -HC=N-), 8.47 (s, 1H, Ar-H), 8.38 (s, 1H, Ar-H), 8.32 (s, 1H, Ar-H), 7.47 (s, 1H, Ar-H), 7.35 (s, 1H, Ar-H), 4.19-4.14 (q, 2H, -OCH_2_-), 1.42-1.39 (t, 3H, -CH_3_). | 160.58 (-C=O-), 159.28 (-HC=N-), 148.63, 148.28, 136.30, 135.18, 134.89, 131.17, 130.84, 128.92, 126.75, 125.12, 125.09, 109.96, 109.92, 65.22 (-OCH_2_), 14.98 (-CH_3_). |
| 6 | [Zn(L^1^)_2_(H_2_O)_2_] | 9.02 (s, 1H, -HC=N-), 8.82 (s, 2H, Ar-H), 8.01 (s, 1H, Ar-H), 7.55 – 7.53 (d, *J* = 7.1 Hz, 1H, Ar-H), 7.36 – 7.34 (d, *J* = 6.5 Hz, 2H, Ar-H), 7.23 – 7.19 (dd, *J* = 6.8, 1.6 Hz, 1H, Ar-H), 7.16 – 7.13 (dd, *J* = 5.2, 1.3 Hz, 1H, Ar-H), 7.06 – 7.04 (d, *J* = 6.2 Hz, 1H, Ar-H), 3.77 (s, 3H, -OCH_3_). | 169.23 (-C-O-), 164.92 (-HC=N-), 155.34, 151.53, 137.65, 133.52, 131.70, 131.59, 131.26, 128.61, 128.41, 127.70, 124.42, 123.97, 122.10, 121.74, 113.09, 112.62, 57.12 (-OCH_3_). |
| 10 | [Zn(L^2^)_2_(H_2_O)_2_] | 8.90 (s, 1H, OH), 7.86 (s, 1H, CH=N-), 7.72 (s, 1H, Ar-H) 7.65 (s, 2H, Ar-H), 7.31 (s, 1H, Ar-H), 7.21 (s, 1H, Ar-H), 3.71-3.66 (q, 2H, -OCH_2_), 1.24-1.21 (t, 3H, -CH_3_). | 176.03 (-C-O-), 163.18 (-HC=N-), 160.77, 148.06, 146.50, 143.04, 141.42, 130.00, 126.64, 124.75, 122.26, 122.23, 113.27, 113.02, 111.68, 110.14, 65.35 (-OCH_2_), 14.99 (-CH_3_). |

**Table S3.** IR bands (cm ^−1^) of the ligands (1-2) and their respective transition metal(II) complexes.

| Sl. No. | Compound | *ʋ*(-HC=N-) | *υ*(N-H) | *ʋ*(C=O)_ketonic_ | *ʋ*(C-O)_enolic_ | *ʋ*(N-N) | *ʋ*(M-O) | *ʋ*(M-N) | *ʋ*(-OH _Water_) |
| --- | --- | --- | --- | --- | --- | --- | --- | --- | --- |
| 1 | HL^1^ | 1598 | 3230 | 1647 | - | 1049 | - | - | - |
| 2 | HL^2^ | 1597 | 3237 | 1656 | - | 1042 | - | - | - |
| 3 | [Co(L^1^)_2_(H_2_O)_2_] | 1595 | - | - | 1277 | 1035 | 534 | 471 | 3455 |
| 4 | [Ni(L^1^)_2_(H_2_O)_2_] | 1593 | - | - | 1295 | 1032 | 537 | 473 | 3450 |
| 5 | [Cu(L^1^)_2_(H_2_O)_2_] | 1597 | - | - | 1281 | 1037 | 539 | 469 | 3459 |
| 6 | [Zn(L^1^)_2_(H_2_O)_2_] | 1592 | - | - | 1279 | 1042 | 536 | 466 | 3452 |
| 7 | [Co(L^2^)_2_(H_2_O)_2_] | 1592 | - | - | 1278 | 1025 | 532 | 471 | 3452 |
| 8 | [Ni(L^2^)2(H_2_O)_2_] | 1593 | - | - | 1285 | 1032 | 530 | 464 | 3445 |
| 9 | [Cu(L^2^)_2_(H_2_O)_2_] | 1595 | - | - | 1287 | 1039 | 535 | 468 | 3442 |
| 10 | [Zn(L^2^)_2_(H_2_O)_2_] | 1591 | - | - | 1292 | 1035 | 534 | 473 | 3447 |

**Table S4.** UV-Vis spectral data, ligand field parameters and magnetic moment of the compounds **(1-10).**

| Compounds | Absorption bands  (cm^-1^) | Transitions | Dq  (cm^-1^) | B  (cm^-1^) | β  (cm^-1^) | β% | Co(υ_3_/υ_1) or_ Ni(υ_2_/υ_1)_ | Geometry | μ (BM) |
| --- | --- | --- | --- | --- | --- | --- | --- | --- | --- |
| HL^1^ | 26,020  38,982 | n→π*  π→π* | - | - | - | - | - | - | - |
| HL^1^ | 26,570  38,890 | n→π*  π→π* | - | - | - | - | - | - | - |
| [Co(L^1^)_2_(H_2_O)_2_] | 10,538  17,338  22,824 | ^4^T_1g_(F)→^4^T_2g_(F) (υ_1_)  ^4^T_1g_(F)→^4^A_2g_(F) (υ_2_)  ^4^T_1g_(F)→ ^4^T_1g_(P) (υ_3_) | 1187.75 | 908.36 | 0.936 | 6.4 | 2.165 | Octahedral | 4.38 |
| [Ni(L^1^)_2_(H_2_O)_2_] | 10,285  18,735  22,985 | ^3^A_2g_(F)→^3^T_2g_(F) (υ_1_)  ^3^A_2g_(F)→3T_1g_(F) (υ_2_)  ^3^A_2g_(F)→^3^T_1g_ (P) (υ_3_) | 1028.5 | 724.33 | 0.703 | 29.7 | 1.821 | Octahedral | 3.23 |
| [Cu(L^1^)_2_(H_2_O)_2_] | 15,556  23,430 | ^2^B_1g_→^2^A_1g_ (υ_1_)  ^2^B_1g_→^2^E_2g_ (υ_2_) | - | - | - | - | - | Octahedral | 1.75 |
| [Zn(L^1^)_2_(H_2_O)_2_] | 23,652 | LMCT | - | - | - | - | - | Octahedral | - |
| [Co(L^2^)_2_(H_2_O)_2_] | 10,509  17,786  22,795 | ^4^T_1g_(F)→^4^T_2g_(F) (υ_1_)  ^4^T_1g_(F)→^4^A_2g_(F) (υ_2_)  ^4^T_1g_(F)→ ^4^T_1g_(P) (υ_3_) | 1184.65 | 908.23 | 0.936 | 6.4 | 2.169 | Octahedral | 4.56 |
| [Ni(L^2^)_2_(H_2_O)_2_] | 10,318  18,915  23,238 | ^3^A_2g_(F)→^3^T_2g_(F) (υ_1_)  ^3^A_2g_(F)→3T_1g_(F) (υ_2_)  ^3^A_2g_(F)→^3^T_1g_ (P) (υ_3_) | 1031.8 | 746.60 | 0.724 | 27.6 | 1.833 | Octahedral | 3.42 |
| [Cu(L^2^)_2_(H_2_O)_2_] | 15,578  23,324 | ^2^B_1g_→^2^A_1g_ (υ_1_)  ^2^B_1g_→^2^E_2g_ (υ_2_) | - | - | - | - | - | Octahedral | 1.80 |
| [Zn(L^2^)_2_(H_2_O)_2_] | 23,584 | LMCT | - | - | - | - | - | Octahedral | - |

**Table S5.** Anti-TB activity results of the compounds **(1-10)** and standard drug as MIC values.

| C. No. | Compounds | MIC value (µmol/mL) |
| --- | --- | --- |
| 1 | HL^1^ | 0.0568 ± 0.0011 |
| 2 | HL^2^ | 0.0251 ± 0.0013 |
| 3 | [Co(L^1^)_2_(H_2_O)_2_] | 0.0256 ± 0.0014 |
| 4 | [Ni(L^1^)_2_(H_2_O)_2_] | 0.0257 ± 0.0010 |
| 5 | [Cu(L^1^)_2_(H_2_O)_2_] | 0.0127 ± 0.0012 |
| 6 | [Zn(L^1^)_2_(H_2_O)_2_] | 0.0063 ± 0.0013 |
| 7 | [Co(L^2^)_2_(H_2_O)_2_] | 0.0114 ± 0.0011 |
| 8 | [Ni(L^2^)_2_(H_2_O)_2_] | 0.0114 ± 0.0016 |
| 9 | [Cu(L^2^)_2_(H_2_O)_2_] | 0.0057 ± 0.0015 |
| 10 | [Zn(L^4^)_2_(H_2_O)_2_] | 0.0028 ± 0.0013 |
| 11 | Streptomycin | 0.0107 ± 0.0011 |

**Table S6.** Antimicrobial activity data (MIC values) of the compounds **(1-10)** and standard drug.

| C. No. | Compounds | Gram +ve bacteria | | Gram -ve bacteria | | Fungi | |
| --- | --- | --- | --- | --- | --- | --- | --- |
|  |  | *S. aureus* | *B. subtilis* | *E. coli* | *P P. aeruginosa* | *R. oryzae* | *C. albicans* |
| 1 | HL^1^ | 0.0568 ± 0.0020 | 0.0568 ± 0.0005 | 0.0568 ± 0.0011 | 0.0568 ± 0.0014 | 0.0284 ± 0.0022 | 0.0284 ± 0.0019 |
| 2 | HL^2^ | 0.0501 ± 0.0015 | 0.0501 ± 0.0011 | 0.0501 ± 0.0010 | 0.0501 ± 0.0018 | 0.0251 ± 0.0019 | 0.0251 ± 0.0018 |
| 3 | [Co(L^1^)_2_(H_2_O)_2_] | 0.0513 ± 0.0012 | 0.0513 ± 0.0021 | 0.0513 ± 0.0015 | 0.0513 ± 0.0015 | 0.0256 ± 0.0018 | 0.0256 ± 0.0016 |
| 4 | [Ni(L^1^)_2_(H_2_O)_2_] | 0.0257 ± 0.0010 | 0.0257 ± 0.0018 | 0.0257 ± 0.0019 | 0.0257 ± 0.0011 | 0.0128 ± 0.0020 | 0.0128 ± 0.0014 |
| 5 | [Cu(L^1^)_2_(H_2_O)_2_] | 0.0255 ± 0.0008 | 0.0255 ± 0.0014 | 0.0255 ± 0.0012 | 0.0255 ± 0.0012 | 0.0127 ± 0.0019 | 0.0127 ± 0.0013 |
| 6 | [Zn(L^1^)_2_(H_2_O)_2_] | 0.0255 ± 0.0017 | 0.0255 ± 0.0012 | 0.0255 ± 0.0017 | 0.0255 ± 0.0017 | 0.0127 ± 0.0018 | 0.0127 ± 0.0015 |
| 7 | [Co(L^2^)_2_(H_2_O)_2_] | 0.0458 ± 0.0016 | 0.0458 ± 0.0013 | 0.0458 ± 0.0012 | 0.0458 ± 0.0013 | 0.0229 ± 0.0014 | 0.0229 ± 0.0008 |
| 8 | [Ni(L^2^)_2_(H_2_O)_2_] | 0.0229 ± 0.0015 | 0.0229 ± 0.0017 | 0.0229 ± 0.0011 | 0.0229 ± 0.0010 | 0.0114 ± 0.0013 | 0.0114 ± 0.0016 |
| 9 | [Cu(L^2^)_2_(H_2_O)_2_] | 0.0228 ± 0.0009 | 0.0228 ± 0.0015 | 0.0228 ± 0.0010 | 0.0228 ± 0.0018 | 0.0114 ± 0.0011 | 0.0114 ± 0.0019 |
| 10 | [Zn(L^4^)_2_(H_2_O)_2_] | 0.0114 ± 0.0011 | 0.0114 ± 0.0012 | 0.0114 ± 0.009 | 0.0114 ± 0.0021 | 0.0057 ± 0.009 | 0.0057 ± 0.0015 |
| 11 | Ciprofloxacin | 0.0047 ± 0.0009 | 0.0047 ± 0.0008 | 0.0047 ± 0.0010 | 0.0047 ± 0.0008 | - | - |
| 12 | Fluconazole | - | - | - | - | 0.0051 ± 0.0011 | 0.0102 ± 0.0012 |

**Table S7.** Anti-inflammatory evaluation results (IC_50_ values) of the compounds **(1-10)** and standard drug.

| C. No. | Compounds | IC_50_ (µM) |
| --- | --- | --- |
| 1 | HL^1^ | 11.41 ± 0.05 |
| 2 | HL^2^ | 09.56 ± 0.10 |
| 3 | [Co(L^1^)_2_(H_2_O)_2_] | 11.08 ± 0.10 |
| 4 | [Ni(L^1^)_2_(H_2_O)_2_] | 10.94 ± 0.12 |
| 5 | [Cu(L^1^)_2_(H_2_O)_2_] | 10.15 ± 0.05 |
| 6 | [Zn(L^1^)_2_(H_2_O)_2_] | 09.82 ± 0.02 |
| 7 | [Co(L^2^)_2_(H_2_O)_2_] | 09.08 ± 0.09 |
| 8 | [Ni(L^2^)_2_(H_2_O)_2_] | 08.12 ± 0.04 |
| 9 | [Cu(L^2^)_2_(H_2_O)_2_] | 07.66 ± 0.08 |
| 10 | [Zn(L^4^)_2_(H_2_O)_2_] | 07.14 ± 0.05 |
| 11 | Diclofenac sodium | 06.44 ± 0.02 |

**Table S8.** Lipophilicity (logP) values of the compounds (1-10).

| C. No. | Compounds | logP |
| --- | --- | --- |
| 1 | HL^1^ | 3.35 |
| 2 | HL^2^ | 3.31 |
| 3 | [Co(L^1^)_2_(H_2_O)_2_] | 16.77 |
| 4 | [Ni(L^1^)_2_(H_2_O)_2_] | 16.77 |
| 5 | [Cu(L^1^)_2_(H_2_O)_2_] | 16.76 |
| 6 | [Zn(L^1^)_2_(H_2_O)_2_] | 16.76 |
| 7 | [Co(L^2^)_2_(H_2_O)_2_] | 16.19 |
| 8 | [Ni(L^2^)_2_(H_2_O)_2_] | 16.19 |
| 9 | [Cu(L^2^)_2_(H_2_O)_2_] | 16.18 |
| 10 | [Zn(L^4^)_2_(H_2_O)_2_] | 16.18 |

#### Table S9. Molecular docking energies and interactions between receptor and compounds (2, 7-10, streptomycin).

| Protein-Ligand  Complex | Binding Energy (Kcal/Mol) (Delta G) | Conventional hydrogen bond | Carbon hydrogen bond | Pi-Pi interaction | Alkyl interaction | Pi- alkyl Interaction | Halogen (Fluorine) | Unfavorable bump |
| --- | --- | --- | --- | --- | --- | --- | --- | --- |
| PDB ID: 5V3Y (Mtb Pks13 Thioesterase Domain) protein receptor from Mycobacterium tuberculosis | | | | | | | | |
| HL^2^ | -137.228 | Ser1533, Asn1640, Ala1667. | -- | Tyr1663, His1664 | Ile1668 | Tyr1663, His1664, Ala1667, Phe1670 | Ala1477(2), His1664 | Ala1667 |
| [Co(L^2^)_2_(H_2_O)_2_] | -123.195 | Gln1633, Ser1636, | Tyr1637 | -- | Val1483, Ile1643, Ile1700 | Trp1532, His1632, Ile1643, Tyr1637, Tyr1663, Ala1667, Phe1670, Tyr1674, His1699, Ile1700, | Gln1633, Tyr1637 | Trp1532, His1632 |
| [Ni(L^2^)_2_(H_2_O)_2_] | -132.8095 | -- | Ser1636, Asn1640 | Ile1700 | Val1483, Ala1667, Ile1700, Ile1703 | His1632, Tyr1637, Tyr1663, Tyr1674 | His1632, Asp1664, Ala1667, Phe1670 | Ile1700, |
| [Cu(L^2^)_2_(H_2_O)_2_] | -159.956 | Asn1640, Tyr1663 | Tyr1637, His1699 | -- | Pro1476, Ala1477, Gly1478, Ala1667, Ile1700 | His1632, Tyr1663, Tyr1637, Phe1670 | Gly1478, Val1562, His1632 | -- |
| [Zn(L^2^)_2_(H_2_O)_2_] | -208.926 | Gln1633, Asn1640, Tyr1663 | Tyr1637 | Trp1532, His1632, Tyr1663, Phe1670, Tyr1674 | Val1483, Arg1641, Ile1643 | Typ1532, His1632, Tyr1637, Phe1670, Tyr1674, Ile1700 | Gly1479, Tyr1637, Asn1640, Phe1670 | -- |
| Streptomycin | -160.616 | Ala1477, Tyr1637, Asn1640, His1664, Asp1666, Glu1671 | Asn1640 | -- | -- | Tyr1637 | -- | -- |

Table S10. DFT descriptors of the compounds (2, 7-10).

| Compound | Total energy | Binding energy | HOMO energy | LUMO energy | Band gap energy | Dipole moment | Hardness | Softness | Electronegativity | Electrophilicity |
| --- | --- | --- | --- | --- | --- | --- | --- | --- | --- | --- |
| HL^2^ | -4186.27 | -8.57466 | -0.202215 | -0.127393 | 0.0748221 | 2.8304 | 0.03741105 | 26.73007039364 | -0.2659115 | 0.00132264757987 |
| [Co(L^2^)_2_(H_2_O)_2_] | -9903.54 | -17.4959 | -0.284438 | -0.263334 | 0.0211044 | 22.3639 | 0.0105522 | 94.76696802562 | -0.152771 | 0.00012313878415 |
| [Ni(L^2^)_2_(H_2_O)_2_] | -10031.8 | -17.9461 | -0.16023 | -0.151084 | 0.00914627 | 1.54976 | 0.004573135 | 218.6683751955 | -0.084688 | 0.00001639939323 |
| [Cu(L^2^)_2_(H_2_O)_2_] | -10178.4 | -20.9083 | -0.173585 | -0.137855 | 0.0357297 | 1.31764 | 0.01786485 | 55.97584082709 | -0.1046575 | 0.00009783856878 |
| [Zn(L^2^)_2_(H_2_O)_2_] | -10298.1 | -15.6094 | -0.674343 | -0.670514 | 0.00382906 | 8.58866 | 0.00191453 | 522.3214052535 | -0.339086 | 0.00011006567435 |

Table S11. Properties of atoms of the compounds (2, 7-10).

| Atom | | Hybridization | Occupancy | | Oxidation state | ESP charge DMol3 | Milliken charge DMol3 | Hirshfeld charge DMol3 |
| --- | --- | --- | --- | --- | --- | --- | --- | --- |
| HL^2^ (2) | | | | | | | | |
| C1  C2  C3  C4  C5  C6  H1  H2  H3  C7  N1  N2  C8  C9  C10  C11  C12  C13  C14  H4  H5  C15  C16  F1  F2  F3  F4  F5  F6  O1  O2  H6  O3  H7  H8  C17  C18  H9  H10  H11  H12  H13  Br1 | | Sp2  Sp2  Sp2  Sp2  Sp2  Sp2  None  None  None  Sp2  Sp2  Sp2  Sp2  Sp2  Sp2  Sp2  Sp2  Sp2  Sp2  None  None  Sp3  Sp3  Sp3  Sp3  Sp3  Sp3  Sp3  Sp3  Sp2  Sp3  None  Sp3  None  None  Sp3  Sp3  None  None  None  None  None  Sp3 | 1  1  1  1  1  1  1  1  1  1  1  1  1  1  1  1  1  1  1  1  1  1  1  1  1  1  1  1  1  1  1  1  1  1  1  1  1  1  1  1  1  1  1 | | 0  0  0  0  0  0  0  0  0  0  0  0  0  0  0  0  0  0  0  0  0  0  0  0  0  0  0  0  0  0  0  0  0  0  0  0  0  0  0  0  0  0  0 | 0.01  -0.079  -0.075  -0.211  -0.24  0.027  0.14  0.125  0.187  0.635  -0.295  -0.241  0.253  -0.213  -0.118  0.284  0.398  -0.312  -0.053  0.138  0.12  0.757  0.801  -0.246  -0.251  -0.251  -0.268  -0.267  -0.262  -0.541  -0.563  0.417  -0.46  0.283  0.055  0.262  -0.507  0.133  0.137  0.163  0.027  0.067  0.037 | -0.174  0.101  0.106  -0.137  -0.204  0.11  0.201  0.25  0.216  0.349  -0.507  -0.031  -0.106  0.087  -0.179  0.268  0.253  -0.301  -0.188  0.161  0.224  0.406  0.401  -0.213  -0.197  -0.196  -0.197  -0.197  -0.221  -0.346  -0.596  0.381  -0.458  0.306  0.13  -0.126  -0.487  0.16  0.183  0.178  0.191  0.188  0.21 | -0.011  -0.008  0.014  -0.023  -0.032  -0.014  0.045  0.055  0.056  0.149  -0.036  -0.094  0.021  -0.002  -0.045  0.077  0.056  -0.032  -0.072  0.043  0.06  0.271  0.27  -0.095  -0.109  -0.109  -0.108  -0.108  -0.102  -0.264  -0.174  0.173  -0.122  0.118  0.039  0.024  -0.109  0.043  0.024  0.038  0.029  0.05  0.024 |
| [Co(L^2^)_2_(H_2_O)_2_] (7) | | | | | | | | |
| C1  C2  C3  C4  C5  C6  H1  H2  H3  C7  C8  F1  F2  F3  F4  F5  F6  C9  O1  N1  N2  C10  C11  H4  C12  C13  C14  C15  C16  H5  H6  Br1  O2  H7  O3  C17  C18  H8  H9  H10  H11  H12  Co1  O4  H13  O5  H14  H15  H16  C19  C20  C21  C22  C23  C24  C25  C26  F7  F8  F9  F10  F11  F12  H17  H18  H19  C27  O6  N3  N4  C28  C29  H20  C30  C31  C32  C33  C34  H21  H22  O7  O8  H23  Br2  C35  C36  H24  H25  H26  H27  H28  H29 | | Sp2  Sp2  Sp2  Sp2  Sp2  Sp2  None  None  None  Sp3  Sp3  Sp3  Sp3  Sp3  Sp3  Sp3  Sp3  Sp2  Sp2  Sp2  Sp2  Sp2  Sp2  None  Sp2  Sp2  Sp2  Sp2  Sp2  None  None  Sp3  Sp3  None  Sp3  Sp3  Sp3  None  None  None  None  None  Octahedral  Sp3  None  Sp3  None  None  None  Sp2  Sp2  Sp2  Sp2  Sp2  Sp2  Sp3  Sp3  Sp3  Sp3  Sp3  Sp3  Sp3  Sp3  None  None  None  Sp3  Sp3  Sp2  Sp2  Sp2  Sp2  None  Sp2  Sp2  Sp2  Sp2  Sp2  None  None  Sp3  Sp3  None  Sp3  Sp3  Sp3  None  None  None  None  None  None | 1  1  1  1  1  1  1  1  1  1  1  1  1  1  1  1  1  1  1  1  1  1  1  1  1  1  1  1  1  1  1  1  1  1  1  1  1  1  1  1  1  1  1  1  1  1  1  1  1  1  1  1  1  1  1  1  1  1  1  1  1  1  1  1  1  1  1  1  1  1  1  1  1  1  1  1  1  1  1  1  1  1  1  1  1  1  1  1  1  1  1  1 | | 0  0  0  0  0  0  0  0  0  0  0  0  0  0  0  0  0  0  0  0  0  0  0  0  0  0  0  0  0  0  0  0  0  0  0  0  0  0  0  0  0  0  0  0  0  0  0  0  0  0  0  0  0  0  0  0  0  0  0  0  0  0  0  0  0  0  0  0  0  0  0  0  0  0  0  0  0  0  0  0  0  0  0  0  0  0  0  0  0  0  0  0 | -0.097  -0.05  -0.064  -0.196  -0.137  -0.013  0.18  0.154  0.134  0.778  0.772  -0.247  -0.242  -0.28  -0.242  -0.255  -0.252  0.677  -0.695  -0.653  0.407  -0.173  0.173  0.119  -0.165  -0.554  -0.259  0.416  0.511  0.265  0.167  0.081  -0.695  0.509  -0.454  0.133  -0.325  0.169  0.119  0.12  0.081  0.093  0.411  -0.851  0.532  -0.864  0.526  0.549  0.563  -0.063  0.071  -0.022  -0.23  -0.171  -0.03  0.629  0.749  -0.24  -0.208  -0.278  -0.236  -0.242  -0.242  0.162  0.203  0.129  0.214  -0.358  0.118  -0.411  0.136  0.106  0.099  -0.449  0.461  0.324  -0.22  -0.205  0.152  0.237  -0.519  -0.525  0.414  0.075  0.242  -0.262  0.095  0.083  0.094  0.059  0.06  0.126 | -0.184  0.097  0.082  -0.149  -0.151  0.096  0.257  0.238  0.231  0.386  0.4  -0.188  -0.19  -0.215  -0.209  -0.192  -0.189  0.174  -0.48  -0.204  -0.326  0.056  0.091  0.207  -0.173  -0.217  -0.316  0.272  0.258  0.196  0.199  0.283  -0.514  0.393  -0.462  -0.118  -0.577  0.214  0.208  0.19  0.212  0.209  0.507  -0.588  0.438  -0.585  0.435  0.436  0.435  -0.148  0.119  -0.006  -0.138  -0.15  0.094  0.39  0.378  -0.184  -0.189  -0.214  -0.185  -0.205  -0.184  0.203  0.267  0.283  0.188  -0.313  -0.444  -0.145  -0.025  0.081  0.175  -0.197  0.3  0.274  -0.313  -0.182  0.182  0.192  -0.523  -0.571  0.397  0.26  -0.104  -0.49  0.186  0.188  0.188  0.207  0.206  0.181 | -0.028  -0.013  0.012  -0.024  -0.016  -0.008  0.057  0.051  0.05  0.267  0.269  -0.102  -0.103  -0.097  -0.092  -0.107  -0.106  0.11  -0.249  -0.162  -0.023  0.055  0.016  0.029  -0.056  -0.057  -0.021  0.074  0.089  0.048  0.047  0.067  -0.172  0.181  -0.102  0.037  -0.121  0.023  0.048  0.036  0.056  0.054  0.169  -0.12  0.201  -0.119  0.198  0.202  0.2  -0.02  -0.011  0.018  -0.024  -0.016  -0.004  0.277  0.268  -0.086  -0.094  -0.054  -0.1  -0.096  -0.1  0.054  0.06  0.036  0.103  -0.145  -0.045  -0.124  0.039  0.009  0.04  -0.044  0.087  0.064  -0.025  -0.068  0.041  0.058  -0.135  -0.154  0.184  0.054  0.044  -0.103  0.035  0.051  0.034  0.052  0.052  0.05 |
| [Ni(L^2^)_2_(H_2_O)_2_] (8) | | | | | | | | |
| C1  C2  C3  C4  C5  C6  H1  H2  H3  C7  C8  C9  C10  C11  C12  H4  H5  C13  C14  C15  C16  C17  C18  H6  H7  C19  C20  C21  C22  C23  C24  H8  H9  H10  Br1  O1  H11  O2  C25  C26  H12  H13  H14  H15  H16  Br2  O3  H17  O4  C27  C28  H18  H19  H20  H21  H22  C29  C30  N1  N2  H23  N3  H24  N4  C31  H25  C32  H26  Ni1  O5  H27  O6  H28  O7  H29  O8  H30  H31  H32  C33  C34  C35  C36  F1  F2  F3  F4  F5  F6  F7  F8  F9  F10  F11  F12  H33  H34 | Sp2  Sp2  Sp2  Sp2  Sp2  Sp2  None  None  None  Sp2  Sp2  Sp2  Sp2  Sp2  Sp2  None  None  Sp2  Sp2  Sp2  Sp2  Sp2  Sp2  None  None  Sp2  Sp2  Sp2  Sp2  Sp2  Sp2  None  None  None  Sp3  Sp3  None  Sp3  Sp3  Sp3  None  None  None  None  None  Sp3  Sp3  None  Sp3  Sp3  Sp3  None  None  None  None  None  Sp3  Sp3  Sp3  Sp3  None  Sp3  None  Sp3  Sp3  None  Sp3  None  Octahedral  Sp3  None  Sp3  None  Sp3  None  Sp3  None  None  None  Sp3  Sp3  Sp3  Sp3  Sp3  Sp3  Sp3  Sp3  Sp3  Sp3  Sp3  Sp3  Sp3  Sp3  Sp3  Sp3  None  None | | 1  1  1  1  1  1  1  1  1  1  1  1  1  1  1  1  1  1  1  1  1  1  1  1  1  1  1  1  1  1  1  1  1  1  1  1  1  1  1  1  1  1  1  1  1  1  1  1  1  1  1  1  1  1  1  1  1  1  1  1  1  1  1  1  1  1  1  1  1  1  1  1  1  1  1  1  1  1  1  1  1  1  1  1  1  1  1  1  1  1  1  1  1  1  1  1  1 | 0  0  0  0  0  0  0  0  0  0  0  0  0  0  0  0  0  0  0  0  0  0  0  0  0  0  0  0  0  0  0  0  0  0  0  0  0  0  0  0  0  0  0  0  0  0  0  0  0  0  0  0  0  0  0  0  0  0  0  0  0  0  0  0  0  0  0  0  0  0  0  0  0  0  0  0  0  0  0  0  0  0  0  0  0  0  0  0  0  0  0  0  0  0  0  0  0 | | -0.282  -0.049  0.137  -0.196  -0.205  -0.076  0.159  0.169  0.168  -0.071  0.157  -0.369  -0.469  0.4  0.266  0.278  0.144  0.085  0.534  -0.253  -0.373  -0.004  -0.043  0.129  0.217  -0.298  0.183  -0.007  -0.311  -0.284  0.054  0.188  0.21  0.176  0.048  -0.698  0.414  -0.375  0.508  -0.749  0.138  0.324  0.168  -0.017  -0.096  0.062  -0.568  0.391  -0.413  0.194  -0.418  0.098  0.166  0.074  -0.006  0.045  -0.18  -0.461  0.139  -0.834  0.074  0.25  0.123  -0.753  0.419  0.41  0.343  0.399  0.854  -0.66  -0.03  -0.518  0.035  -0.913  0.519  -0.913  0.502  0.52  0.503  0.79  0.697  0.812  0.804  -0.259  -0.248  -0.254  -0.237  -0.251  -0.103  -0.207  -0.256  -0.25  -0.257  -0.258  -0.264  0.107  0.149 | -0.171  0.094  0.075  -0.198  -0.12  0.081  0.263  0.18  0.276  -0.187  0.102  -0.335  -0.254  0.269  0.241  0.263  0.172  0.254  0.301  -0.224  -0.335  0.118  -0.193  0.166  0.268  -0.169  0.082  0.093  -0.142  -0.184  0.07  0.256  0.249  0.189  0.245  -0.595  0.351  -0.521  -0.211  -0.537  0.147  0.256  0.173  0.167  0.208  0.246  -0.596  0.354  -0.49  -0.257  -0.513  0.148  0.266  0.161  0.143  0.266  -0.311  -0.336  -0.391  -0.331  0.183  -0.383  0.189  -0.311  -0.016  0.279  0.025  0.275  0.813  -0.554  0.156  -0.51  0.195  -0.598  0.403  -0.555  0.399  0.427  0.377  0.351  0.379  0.257  0.345  -0.203  -0.183  -0.181  -0.185  -0.177  -0.171  -0.127  -0.147  -0.194  -0.183  -0.184  -0.21  0.207  0.216 | -0.039  -0.03  0.019  -0.066  -0.023  -0.024  0.037  0.043  0.022  -0.078  0.012  -0.026  -0.072  0.046  0.068  0.011  0.043  0.069  0.045  -0.059  -0.025  0.023  -0.076  0.042  0.017  -0.036  0.019  -0.025  -0.019  -0.06  -0.023  0.043  0.026  0.046  0.042  -0.103  0.143  -0.092  -0.046  -0.189  0.018  0.009  0.003  0.022  -0.005  0.043  -0.126  0.143  -0.121  -0.054  -0.16  0.028  0.008  0.014  0.005  0.029  -0.016  -0.023  -0.095  -0.116  0.018  -0.089  0.018  -0.105  0.073  0.099  0.078  0.1  0.245  -0.261  0.014  -0.221  0.019  -0.123  0.189  -0.13  0.183  0.187  0.155  0.259  0.263  0.274  0.257  -0.099  -0.1  -0.098  -0.094  -0.096  0.057  -0.019  -0.028  -0.08  -0.1  -0.104  -0.102  0.026  0.029 |
| [Cu(L^2^)_2_(H_2_O)_2_] (9) | | | | | | | | |
| C1  C2  C3  C4  C5  C6  C7  N1  N2  C8  C9  C10  C11  C12  C13  C14  C15  C16  C17  C18  C19  C20  C21  C22  C23  C24  C25  C26  C27  N3  N4  C28  C29  C30  C31  C32  C33  C34  C35  C36  H1  H2  H3  F1  F2  F3  Br1  O1  H4  O2  H5  H6  H7  H8  H9  H10  H11  H12  H13  H14  H15  F4  F5  F6  F7  F8  F9  F10  F11  F12  H16  H17  H18  O3  O4  H19  Br2  H20  H21  H22  H23  H24  Cu1  O5  O6  O7  H25  O8  H26  H27  H28  H29  H30  H31  H32  H33  H34  H35  H36  H37  H38  H39  H40  H41  H42  H43  H44  H45  H46  H47  H48  H49  H50  H51  H52  H53  H54  H55  H56  H57  H58 | Sp3  Sp3  Sp3  Sp3  Sp3  Sp3  Sp3  Sp3  Sp3  Sp3  Sp3  Sp3  Sp3  Sp3  Sp3  Sp3  Sp3  Sp3  Sp3  Sp3  Sp3  Sp3  Sp3  Sp3  Sp3  Sp3  Sp3  Sp3  Sp3  Sp3  Sp3  Sp3  Sp3  Sp3  Sp3  Sp3  Sp3  Sp3  Sp3  Sp3  None  None  None  Sp3  Sp3  Sp3  Sp3  Sp3  None  Sp3  None  None  None  None  None  None  None  None  None  None  None  Sp3  Sp3  Sp3  Sp3  Sp3  Sp3  Sp3  Sp3  Sp3  None  None  None  Sp3  Sp3  None  Sp3  None  None  None  None  None  Octahedral  Sp3  Sp3  Sp3  None  Sp3  None  None  None  None  None  None  None  None  None  None  None  None  None  None  None  None  None  None  None  None  None  None  None  None  None  None  None  None  None  None  None  None  None | | 1  1  1  1  1  1  1  1  1  1  1  1  1  1  1  1  1  1  1  1  1  1  1  1  1  1  1  1  1  1  1  1  1  1  1  1  1  1  1  1  1  1  1  1  1  1  1  1  1  1  1  1  1  1  1  1  1  1  1  1  1  1  1  1  1  1  1  1  1  1  1  1  1  1  1  1  1  1  1  1  1  1  1  1  1  1  1  1  1  1  1  1  1  1  1  1  1  1  1  1  1  1  1  1  1  1  1  1  1  1  1  1  1  1  1  1  1  1  1  1  1 | 0  0  0  0  0  0  0  0  0  0  0  0  0  0  0  0  0  0  0  0  0  0  0  0  0  0  0  0  0  0  0  0  0  0  0  0  0  0  0  0  0  0  0  0  0  0  0  0  0  0  0  0  0  0  0  0  0  0  0  0  0  0  0  0  0  0  0  0  0  0  0  0  0  0  0  0  0  0  0  0  0  0  0  0  0  0  0  0  0  0  0  0  0  0  0  0  0  0  0  0  0  0  0  0  0  0  0  0  0  0  0  0  0  0  0  0  0  0  0  0  0 | | -0.058  -0.621  0.432  0.026  -0.463  0.516  -0.082  -0.235  -0.562  -0.014  0.251  -0.4  -0.24  -0.256  -0.197  -0.218  0.707  0.251  -0.498  -0.104  -0.266  0.071  -0.206  -0.425  0.045  0.042  0.873  0.768  0.746  -0.594  -0.193  -0.203  0.393  -0.766  0.761  -0.011  -0.437  -0.09  0.023  -0.513  0.089  0.131  0.049  -0.246  -0.211  -0.253  -0.014  -0.627  0.432  -0.577  0.138  0.124  0.156  0.094  0.013  0.071  0.127  0.056  0.126  0.105  0.094  -0.28  -0.271  -0.284  -0.29  -0.259  -0.266  -0.236  -0.243  -0.24  0.134  0.08  0.206  -0.558  -0.715  0.46  -0.032  0.165  0.144  0.155  0.128  0.086  0.258  -0.393  -0.26  -0.632  0.366  -0.863  0.526  0.549  0.438  0.057  0.362  -0.145  0.16  0.114  -0.02  0.06  0.402  0.096  0.092  0.168  0.183  0.137  0.166  0.113  0.035  0.144  0.086  0.102  0.146  0.187  0.197  0.368  0.126  0  0.169  0.013  0.144  0.254  0.079 | -0.294  -0.536  -0.207  0.013  -0.33  -0.11  -0.316  -0.201  -0.347  -0.036  -0.148  -0.408  -0.198  -0.38  -0.213  -0.343  0.389  -0.19  -0.561  -0.45  -0.196  -0.156  -0.344  -0.343  -0.324  0.021  0.365  0.402  0.397  -0.362  -0.226  -0.218  -0.178  -0.351  -0.046  -0.032  -0.491  -0.286  -0.179  -0.538  0.208  0.193  0.182  -0.18  -0.175  -0.177  0.123  -0.532  0.341  -0.376  0.193  0.192  0.188  0.187  0.225  0.241  0.245  0.241  0.237  0.247  0.196  -0.176  -0.174  -0.177  -0.19  -0.165  -0.176  -0.179  -0.176  -0.181  0.162  0.188  0.206  -0.387  -0.531  0.34  0.133  0.175  0.198  0.193  0.205  0.178  0.294  -0.379  -0.428  -0.588  0.336  -0.698  0.437  0.449  0.377  0.162  0.312  0.287  0.178  0.18  0.217  0.18  0.296  0.141  0.222  0.191  0.231  0.232  0.221  0.269  0.225  0.231  0.204  0.222  0.199  0.32  0.202  0.31  0.205  0.236  0.172  0.218  0.191  0.239  0.192 | -0.075  -0.013  -0.014  0.028  -0.068  0.049  -0.03  -0.113  -0.104  0.059  -0.019  -0.067  -0.031  -0.065  -0.033  -0.064  0.254  0.014  -0.108  -0.066  -0.032  -0.011  -0.064  -0.062  -0.033  0.055  0.249  0.255  0.251  -0.089  -0.118  -0.035  -0.01  -0.061  0.05  0.029  -0.005  -0.075  0.013  -0.107  0.032  0.032  0.017  -0.097  -0.073  -0.08  -0.02  -0.238  0.133  -0.161  0.041  0.019  0.037  0.048  0.027  0.028  0.034  0.034  0.038  0.024  0.035  -0.082  -0.094  -0.092  -0.043  -0.073  -0.093  -0.084  -0.094  -0.091  0.018  0.025  0.042  -0.154  -0.24  0.133  -0.014  0.045  0.038  0.034  0.029  0.024  0.479  -0.263  -0.258  -0.211  0.173  -0.242  0.169  0.164  0.123  0.016  0.012  0.015  0.024  0.031  0.018  0.032  0.106  0.009  0.021  0.039  0.048  0.029  0.04  0.035  0.025  0.043  0.035  0.038  0.034  0.034  0.027  0.096  0.017  0.027  0.032  0.031  0.023  0.035  0.025 |
| [Zn(L^2^)_2_(H_2_O)_2_] (10) | | | | | | | | |
| C1  C2  C3  C4  C5  C6  H1  H2  C7  C8  C9  C10  C11  C12  H3  H4  H5  Br1  O1  H6  O2  C13  C14  H7  H8  H9  H10  H11  C15  C16  N1  N2  H12  C17  C18  F1  F2  F3  F4  F5  F6  O3  C19  C20  C21  C22  C23  C24  H13  H14  H15  C25  C26  C27  N3  N4  C28  C29  C30  C31  C32  C33  C34  H16  H17  H18  H19  Zn1  O4  O5  H20  O6  H21  O7  O8  H22  H23  H24  C35  C36  H25  H26  H27  H28  H29  Br2  F7  F8  F9  F10  F11  F12 | Sp2  Sp2  Sp2  Sp2  Sp2  Sp2  None  None  Sp2  Sp2  Sp2  Sp2  Sp2  Sp2  None  None  None  Sp3  Sp3  None  Sp3  Sp3  Sp3  None  None  None  None  None  Sp2  Sp2  Sp2  Sp2  None  Sp3  Sp3  Sp3  Sp3  Sp3  Sp3  Sp3  Sp3  Sp2  Sp2  Sp2  Sp2  Sp2  Sp2  Sp2  None  None  None  Sp3  Sp3  Sp2  Sp2  Sp2  Sp2  Sp2  Sp2  Sp2  Sp2  Sp2  Sp2  None  None  None  None  Octahedral  Sp2  Sp3  None  Sp3  None  Sp3  Sp3  None  None  None  Sp3  Sp3  None  None  None  None  None  Sp3  Sp3  Sp3  Sp3  Sp3  Sp3  Sp3 | | 1  1  1  1  1  1  1  1  1  1  1  1  1  1  1  1  1  1  1  1  1  1  1  1  1  1  1  1  1  1  1  1  1  1  1  1  1  1  1  1  1  1  1  1  1  1  1  1  1  1  1  1  1  1  1  1  1  1  1  1  1  1  1  1  1  1  1  1  1  1  1  1  1  1  1  1  1  1  1  1  1  1  1  1  1  1  1  1  1  1  1  1 | 0  0  0  0  0  0  0  0  0  0  0  0  0  0  0  0  0  0  0  0  0  0  0  0  0  0  0  0  0  0  0  0  0  0  0  0  0  0  0  0  0  0  0  0  0  0  0  0  0  0  0  0  0  0  0  0  0  0  0  0  0  0  0  0  0  0  0  0  0  0  0  0  0  0  0  0  0  0  0  0  0  0  0  0  0  0  0  0  0  0  0  0 | | 0.209  -0.584  0.131  0.819  -0.219  0.064  0.134  0.193  0.029  -0.012  -0.151  0.037  0.092  -0.15  0.172  0.234  0.12  0.456  -0.637  0.518  -0.054  0.057  -0.63  0.247  0.26  0.244  0.198  0.196  -0.307  0.619  0.489  -0.551  0.22  0.645  0.663  -0.187  -0.154  -0.162  -0.155  -0.194  -0.286  -0.609  -0.452  0.293  0.555  -0.164  -0.246  -0.002  0.17  0.222  0.216  0.5  0.734  -0.603  0.759  -0.229  0.107  0.027  -0.358  0.581  0.461  -0.418  0.108  0.076  0.209  0.329  0.149  0.668  -0.551  -0.838  0.494  -0.706  0.453  -0.345  -0.477  0.471  0.431  0.499  0.069  -0.4  0.178  0.2  0.185  0.215  0.206  0.401  -0.163  -0.217  -0.22  -0.183  -0.18  -0.222 | -0.098  -0.347  0.084  0.316  -0.167  0.283  0.272  0.266  0.099  -0.121  -0.06  0.117  0.116  -0.173  0.268  0.311  0.279  0.615  -0.472  0.452  -0.306  -0.253  -0.534  0.265  0.28  0.257  0.3  0.3  0.067  0.311  -0.322  -0.142  0.263  0.406  0.404  -0.181  -0.129  -0.135  -0.138  -0.148  -0.23  -0.483  -0.172  0.112  0.096  -0.076  -0.151  0.102  0.25  0.316  0.284  0.356  0.411  0.027  -0.022  -0.33  0.005  0.098  -0.172  0.314  0.331  -0.344  -0.133  0.238  0.295  0.262  0.296  0.65  -0.302  -0.63  0.443  -0.622  0.436  -0.36  -0.475  0.447  0.436  0.438  -0.2  -0.526  0.24  0.285  0.25  0.319  0.318  0.566  -0.167  -0.181  -0.18  -0.14  -0.143  -0.188 | 0.019  0.017  0.048  0.14  0.008  0.139  0.09  0.074  0.048  0.029  0.054  0.038  0.031  0.004  0.047  0.1  0.076  0.343  -0.055  0.228  -0.004  0.057  -0.075  0.062  0.101  0.054  0.093  0.093  0.084  0.157  -0.023  -0.09  0.077  0.279  0.276  -0.07  -0.054  -0.057  -0.061  -0.069  -0.1  -0.2  -0.003  0.02  0.036  0.048  0.016  0.038  0.072  0.095  0.041  0.28  0.278  0.073  0.154  -0.043  0.084  0.028  0.014  0.136  0.137  0.017  -0.008  0.062  0.079  0.081  0.061  0.383  -0.177  -0.169  0.199  -0.17  0.196  0.002  -0.056  0.226  0.197  0.198  0.085  -0.079  0.065  0.101  0.067  0.124  0.126  0.299  -0.043  -0.077  -0.083  -0.063  -0.063  -0.077 |

Table S12. Properties of bonds of the compounds (2, 7-10).

| Bond | Bond order | Bond type | Bond length |
| --- | --- | --- | --- |
| HL^2^ (2) | | | |
| C1 - C2  C1 - C3  C1 - H2  C2 - C4  C2 - C15  C3 - C5  C3 - C7  C4 - C6  C4 - H3  C5 - C6  C5 - H1  C6 - C16  C7 - N1  C7 - O1  N1 - N2  N1 - H7  N2 - C8  C8 - C9  C8 - H8  C9 - C10  C9 - C14  C10 - C11  C10 - H5  C11 - C12  C11 - O3  C12 - C13  C12 - O2  C13 - C14  C13 - Br1  C14 - H4  C15 - F1  C15 - F2  C15 - F3  C16 - F4  C16 - F5  C16 - F6  O2 - H6  O3 - C17  C17 - C18  C17 - H12  C17 - H13  C18 - H9  C18 - H10  C18 - H11 | 2  1  1  1  1  2  1  2  1  1  1  1  1  2  1  1  2  1  1  2  1  1  1  2  1  1  1  2  1  1  1  1  1  1  1  1  1  1  1  1  1  1  1  1 | Double  Single  Single  Single  Single  Double  Single  Double  Single  Single  Single  Single  Single  Double  Single  Single  Double  Single  Single  Double  Single  Single  Single  Double  Single  Single  Single  Double  Single  Single  Single  Single  Single  Single  Single  Single  Single  Single  Single  Single  Single  Single  Single  Single | 1.40572  1.41049  1.08149  1.4001  1.50898  1.41294  1.50283  1.40109  1.08401  1.40633  1.07599  1.51005  1.36192  1.2249  1.37793  1.02749  1.28761  1.48053  1.08779  1.40341  1.39811  1.40109  1.08371  1.41994  1.3638  1.41173  1.35052  1.39743  1.91874  1.08321  1.38453  1.38325  1.38289  1.38267  1.38279  1.38468  0.965783  1.38296  1.52856  1.10982  1.10969  1.11  1.1076  1.1103 |
| [Co(L^2^)_2_(H_2_O)_2_] (7) |  |  |  |
| C1 - C2  C1 - C3  C1 - H2  C2 - C4  C2 - C7  C3 - C5  C3 - C9  C4 - C6  C4 - H1  C5 - C6  C5 - H3  C6 - C8  C7 - F1  C7 - F2  C7 - F3  C8 - F4  C8 - F5  C8 - F6  C9 - O1  C9 - N1  O1 - Co1  N1 - N2  N2 - C10  N2 - Co1  C10 - C11  C10 - H4  C11 - C12  C11 - C13  C12 - C14  C12 - H6  C13 - C16  C13 - H5  C14 - C15  C14 - Br1  C15 - C16  C15 - O2  C16 - O3  O2 - H7  O3 - C17  C17 - C18  C17 - H11  C17 - H12  C18 - H8  C18 - H9  C18 - H10  O4 - Co1  O4 - H13  O4 - H15  O5 - Co1  O5 - H14  O5 - H16  C19 - C20  C19 - C21  C19 - H19  C20 - C22  C20 - C25  C21 - C23  C21 - C27  C22 - C24  C22 - H18  C23 - C24  C23 - H17  C24 - C26  C25 - F7  C25 - F8  C25 - F9  C26 - F10  C26 - F11  C26 - F12  C27 - O6  C27 - N3  C27 - H29  O6 - Co1  N3 - Co1  N3 - N4  N4 - C28  C28 - C29  C28 - H20  C29 - C30  C29 - C34  C30 - C31  C30 - H22  C31 - C32  C31 - O7  C32 - C33  C32 - O8  C33 - C34  C33 - Br2  C34 - H21  O7 - C35  O8 - H23  C35 - C36  C35 - H27  C35 - H28  C36 - H24  C36 - H25  C36 - H26 | 2  1  1  1  1  2  1  2  1  1  1  1  1  1  1  1  1  1  1  2  1  1  2  1  1  1  2  1  1  1  2  1  2  1  1  1  1  1  1  1  1  1  1  1  1  1  1  1  1  1  1  2  1  1  1  1  2  1  2  1  1  1  1  1  1  1  1  1  1  1  1  1  1  1  1  2  1  1  2  1  1  1  2  1  1  1  2  1  1  1  1  1  1  1  1  1  1 | Double  Single  Single  Single  Single  Double  Single  Double  Single  Single  Single  Single  Single  Single  Single  Single  Single  Single  Single  Double  Single  Single  Double  Single  Single  Single  Double  Single  Single  Single  Double  Single  Double  Single  Single  Single  Single  Single  Single  Single  Single  Single  Single  Single  Single  Single  Single  Single  Single  Single  Single  Double  Single  Single  Single  Single  Double  Single  Double  Single  Single  Single  Single  Single  Single  Single  Single  Single  Single  Single  Single  Single  Single  Single  Single  Double  Single  Single  Double  Single  Single  Single  Double  Single  Single  Single  Double  Single  Single  Single  Single  Single  Single  Single  Single  Single  Single | 1.4097  1.39717  1.07928  1.39895  1.50247  1.43355  1.50871  1.39719  1.08453  1.41577  1.08593  1.52425  1.38266  1.38369  1.3823  1.38798  1.3823  1.38211  1.36118  1.2677  1.85163  1.38576  1.36882  2.05165  1.60918  1.07876  1.44932  1.46161  1.38211  1.08096  1.47886  1.0778  1.37088  1.9258  1.39621  1.36084  1.37208  0.967973  1.3749  1.47647  1.11491  1.11128  1.09634  1.11023  1.10985  1.85715  0.990495  0.985574  1.86015  0.991107  0.985614  1.39993  1.4549  1.07232  1.35596  1.50794  1.45402  1.61921  1.39155  1.0924  1.46809  1.07702  1.52084  1.38538  1.3857  1.36903  1.38983  1.37347  1.39169  1.39249  1.37829  1.08997  1.71944  1.98511  1.47076  1.35117  1.66622  1.08206  1.49026  1.4272  1.49644  1.07979  1.39905  1.40556  1.40453  1.35614  1.41991  1.92431  1.07745  1.44572  0.968856  1.57044  1.11143  1.11206  1.11121  1.12584  1.11103 |
| [Ni(L^2^)_2_(H_2_O)_2_] (8) |  |  |  |
| C1 - C2  C1 - C3  C1 - H2  C2 - C4  C2 - C36  C3 - C5  C3 - C32  C4 - C6  C4 - H1  C5 - C6  C5 - H3  C6 - C35  C7 - C8  C7 - C9  C7 - H5  C8 - C10  C8 - C30  C9 - C11  C9 - Br2  C10 - C12  C10 - H4  C11 - C12  C11 - O3  C12 - O4  C13 - C14  C13 - C15  C13 - O2  C14 - C16  C14 - O1  C15 - C17  C15 - H7  C16 - C18  C16 - Br1  C17 - C18  C17 - C29  C18 - H6  C19 - C20  C19 - C21  C19 - H10  C20 - C22  C20 - C31  C21 - C23  C21 - C33  C22 - C24  C22 - H9  C23 - C24  C23 - H8  C24 - C34  O1 - H11  O2 - C25  C25 - C26  C25 - H15  C25 - H16  C26 - H12  C26 - H13  C26 - H14  O3 - H17  O4 - C27  C27 - C28  C27 - H21  C27 - H22  C28 - H18  C28 - H19  C28 - H20  C29 - N1  C29 - H23  C29 - H33  C30 - N3  C30 - H24  C30 - H34  N1 - N2  N1 - Ni1  N2 - C31  N2 - H25  N3 - N4  N3 - Ni1  N4 - C32  N4 - H26  C31 - O5  C31 - H27  C32 - O6  C32 - H28  O5 - Ni1  O6 - Ni1  O7 - Ni1  O7 - H29  O7 - H31  O8 - Ni1  O8 - H30  O8 - H32  C33 - F1  C33 - F2  C33 - F3  C34 - F4  C34 - F5  C34 - F6  C35 - F7  C35 - F8  C35 - F9  C36 - F10  C36 - F11  C36 - F12 | 2  1  1  1  1  2  1  2  1  1  1  1  2  1  1  1  1  2  1  2  1  1  1  1  2  1  1  1  1  2  1  2  1  1  1  1  2  1  1  1  1  2  1  2  1  1  1  1  1  1  1  1  1  1  1  1  1  1  1  1  1  1  1  1  1  1  1  1  1  1  1  1  1  1  1  1  1  1  1  1  1  1  1  1  1  1  1  1  1  1  1  1  1  1  1  1  1  1  1  1  1  1 | Double  Single  Single  Single  Single  Double  Single  Double  Single  Single  Single  Single  Double  Single  Single  Single  Single  Double  Single  Double  Single  Single  Single  Single  Double  Single  Single  Single  Single  Double  Single  Double  Single  Single  Single  Single  Double  Single  Single  Single  Single  Double  Single  Double  Single  Single  Single  Single  Single  Single  Single  Single  Single  Single  Single  Single  Single  Single  Single  Single  Single  Single  Single  Single  Single  Single  Single  Single  Single  Single  Single  Single  Single  Single  Single  Single  Single  Single  Single  Single  Single  Single  Single  Single  Single  Single  Single  Single  Single  Single  Single  Single  Single  Single  Single  Single  Single  Single  Single  Single  Single  Single | 1.58193  1.57827  1.06952  1.34675  1.55979  1.56662  1.64957  1.35235  1.07499  1.58493  1.06655  1.55146  1.40915  1.39776  1.07175  1.41878  1.58315  1.39878  1.91285  1.44464  1.06714  1.41781  1.43391  1.45496  1.37703  1.44358  1.45595  1.35851  1.43272  1.41057  1.06708  1.39791  1.91377  1.40571  1.57775  1.0712  1.58115  1.57886  1.06903  1.56716  1.65591  1.34589  1.56031  1.58991  1.06662  1.3546  1.07562  1.55237  0.96576  1.45684  1.55006  1.06921  1.07114  1.07518  1.06568  1.07193  0.965033  1.45771  1.55113  1.06948  1.0694  1.07517  1.06593  1.0724  1.3885  1.09  1.09  1.39143  1.09001  1.08997  1.40816  1.9221  1.44669  1.00558  1.42057  1.92273  1.45351  0.99793  1.49508  1.06812  1.47936  1.07073  1.92347  1.91369  1.80877  0.957213  0.961825  1.81155  0.958347  0.96126  1.34578  1.3546  1.35484  1.35468  1.35446  1.34378  1.35065  1.34508  1.35717  1.35531  1.35495  1.34576 |
| [Cu(L^2^)_2_(H_2_O)_2_] (9) |  |  |  |
| C1 - C2  C1 - C3  C1 - H1  C1 - H29  C2 - C4  C2 - Br1  C2 - H30  C3 - C5  C3 - C7  C3 - H31  C4 - C6  C4 - O1  C4 - H32  C5 - C6  C5 - H2  C5 - H33  C6 - O2  C6 - H34  C7 - N1  C7 - H3  C7 - H35  N1 - N2  N1 - Cu1  N2 - C8  N2 - H36  C8 - C9  C8 - O5  C8 - H37  C9 - C10  C9 - C14  C9 - H38  C10 - C11  C10 - H14  C10 - H39  C11 - C12  C11 - C27  C11 - H40  C12 - C13  C12 - H13  C12 - H41  C13 - C14  C13 - C15  C13 - H42  C14 - H15  C14 - H43  C15 - F1  C15 - F2  C15 - F3  C16 - C17  C16 - O2  C16 - H8  C16 - H9  C17 - H5  C17 - H6  C17 - H7  C18 - C19  C18 - C20  C18 - H12  C18 - H44  C19 - C21  C19 - C25  C19 - H45  C20 - C22  C20 - C24  C20 - H46  C21 - C23  C21 - H10  C21 - H47  C22 - C23  C22 - H11  C22 - H48  C23 - C26  C23 - H49  C24 - N3  C24 - O6  C24 - H50  C25 - F4  C25 - F5  C25 - F6  C26 - F7  C26 - F8  C26 - F9  C27 - F10  C27 - F11  C27 - F12  N3 - N4  N3 - H51  N4 - C28  N4 - Cu1  C28 - C29  C28 - H16  C28 - H52  C29 - C30  C29 - C34  C29 - H53  C30 - C31  C30 - H18  C30 - H54  C31 - C32  C31 - O3  C31 - H55  C32 - C33  C32 - O4  C32 - H56  C33 - C34  C33 - Br2  C33 - H57  C34 - H17  C34 - H58  C35 - C36  C35 - O3  C35 - H23  C35 - H24  C36 - H20  C36 - H21  C36 - H22  O1 - H4  O4 - H19  O5 - Cu1  O6 - Cu1  O7 - Cu1  O7 - H25  O7 - H28  O8 - Cu1  O8 - H26  O8 - H27 | 1  1  1  1  1  1  1  1  1  1  1  1  1  1  1  1  1  1  1  1  1  1  1  1  1  1  1  1  1  1  1  1  1  1  1  1  1  1  1  1  1  1  1  1  1  1  1  1  1  1  1  1  1  1  1  1  1  1  1  1  1  1  1  1  1  1  1  1  1  1  1  1  1  1  1  1  1  1  1  1  1  1  1  1  1  1  1  1  1  1  1  1  1  1  1  1  1  1  1  1  1  1  1  1  1  1  1  1  1  1  1  1  1  1  1  1  1  1  1  1  1  1  1  1  1  1 | Single  Single  Single  Single  Single  Single  Single  Single  Single  Single  Single  Single  Single  Single  Single  Single  Single  Single  Single  Single  Single  Single  Single  Single  Single  Single  Single  Single  Single  Single  Single  Single  Single  Single  Single  Single  Single  Single  Single  Single  Single  Single  Single  Single  Single  Single  Single  Single  Single  Single  Single  Single  Single  Single  Single  Single  Single  Single  Single  Single  Single  Single  Single  Single  Single  Single  Single  Single  Single  Single  Single  Single  Single  Single  Single  Single  Single  Single  Single  Single  Single  Single  Single  Single  Single  Single  Single  Single  Single  Single  Single  Single  Single  Single  Single  Single  Single  Single  Single  Single  Single  Single  Single  Single  Single  Single  Single  Single  Single  Single  Single  Single  Single  Single  Single  Single  Single  Single  Single  Single  Single  Single  Single  Single  Single  Single | 1.51276  1.52615  1.08996  1.09002  1.51022  1.93452  1.09001  1.52301  1.53019  1.09002  1.51711  1.40816  1.09003  1.52037  1.09001  1.09002  1.39785  1.09003  1.44631  1.09001  1.08998  1.38802  1.85456  1.43991  1.07001  1.51699  1.39481  1.09  1.52207  1.52512  1.09  1.52801  1.09003  1.08998  1.5309  1.4649  1.09001  1.53034  1.08999  1.09003  1.53002  1.4643  1.09001  1.09004  1.08998  1.30194  1.30229  1.30218  1.52646  1.40429  1.11463  1.11292  1.11459  1.11343  1.11359  1.52676  1.52641  1.08995  1.09001  1.5283  1.46053  1.08999  1.52573  1.51703  1.08997  1.52914  1.09001  1.08999  1.52694  1.09004  1.09002  1.45968  1.08998  1.44183  1.39846  1.09  1.30147  1.30226  1.30221  1.30171  1.3019  1.30228  1.30278  1.30198  1.30237  1.38648  1.06997  1.44448  1.85454  1.52263  1.08999  1.09  1.52151  1.52371  1.09  1.51879  1.09007  1.09005  1.51619  1.39528  1.08997  1.5086  1.40772  1.09001  1.51507  1.93214  1.09  1.09002  1.09001  1.52651  1.40357  1.11498  1.11253  1.11475  1.11359  1.1137  0.960421  0.961007  1.81649  1.81637  1.85124  1.03991  1.00004  1.84462  1.03309  1.03495 |
| [Zn(L^2^)_2_(H_2_O)_2_] (10) |  |  |  |
| C1 - C2  C1 - C3  C1 - H1  C2 - C4  C2 - Br1  C3 - C5  C3 - C15  C4 - C6  C4 - O1  C5 - C6  C5 - H2  C6 - O2  C7 - C8  C7 - C9  C7 - C17  C8 - C10  C8 - H5  C9 - C11  C9 - H4  C10 - C12  C10 - C16  C11 - C12  C11 - C18  C12 - H3  O1 - H6  O2 - C13  C13 - C14  C13 - H10  C13 - H11  C14 - H7  C14 - H8  C14 - H9  C15 - N1  C15 - H12  C16 - N2  C16 - O3  N1 - N2  N1 - Zn1  C17 - F1  C17 - F2  C17 - F3  C18 - F4  C18 - F5  C18 - F6  O3 - Zn1  C19 - C20  C19 - C21  C19 - H15  C20 - C22  C20 - C25  C21 - C23  C21 - C27  C22 - C24  C22 - H14  C23 - C24  C23 - H13  C24 - C26  C25 - F7  C25 - F8  C25 - F9  C26 - F10  C26 - F11  C26 - F12  C27 - N3  C27 - H18  N3 - N4  N3 - O4  N4 - C28  N4 - Zn1  C28 - C29  C28 - H19  C29 - C30  C29 - C34  C30 - C31  C30 - H17  C31 - C32  C31 - O7  C32 - C33  C32 - O8  C33 - C34  C33 - Br2  C34 - H16  O4 - Zn1  O5 - Zn1  O5 - H20  O5 - H24  O6 - Zn1  O6 - H21  O6 - H23  O7 - C35  O8 - H22  C35 - C36  C35 - H28  C35 - H29  C36 - H25  C36 - H26  C36 - H27 | 2  1  1  1  1  2  1  2  1  1  1  1  2  1  1  1  1  2  1  2  1  1  1  1  1  1  1  1  1  1  1  1  2  1  2  1  1  1  1  1  1  1  1  1  1  2  1  1  1  1  2  1  2  1  1  1  1  1  1  1  1  1  1  2  1  1  1  2  1  1  1  2  1  1  1  2  1  1  1  2  1  1  1  1  1  1  1  1  1  1  1  1  1  1  1  1  1 | Double  Single  Single  Single  Single  Double  Single  Double  Single  Single  Single  Single  Double  Single  Single  Single  Single  Double  Single  Double  Single  Single  Single  Single  Single  Single  Single  Single  Single  Single  Single  Single  Double  Single  Double  Single  Single  Single  Single  Single  Single  Single  Single  Single  Single  Double  Single  Single  Single  Single  Double  Single  Double  Single  Single  Single  Single  Single  Single  Single  Single  Single  Single  Double  Single  Single  Single  Double  Single  Single  Single  Double  Single  Single  Single  Double  Single  Single  Single  Double  Single  Single  Single  Single  Single  Single  Single  Single  Single  Single  Single  Single  Single  Single  Single  Single  Single | 1.37481  1.4145  1.08397  1.39023  1.92491  1.42034  1.50968  1.43288  1.35145  1.43772  1.08102  1.37543  1.40984  1.40664  1.50612  1.40176  1.08212  1.40047  1.08381  1.40926  1.48293  1.39968  1.51175  1.08182  0.966003  1.39684  1.51744  1.112  1.11257  1.10763  1.11103  1.10642  1.30867  1.09358  1.27616  1.30643  1.39052  2.02757  1.38359  1.38257  1.38335  1.383  1.38264  1.38578  1.72123  1.39875  1.3976  1.07696  1.40146  1.48981  1.41068  1.48484  1.39836  1.08299  1.40558  1.08279  1.50907  1.3856  1.38139  1.38449  1.38331  1.38415  1.38323  1.29125  1.05619  1.39506  1.33455  1.30066  1.87426  1.51734  1.07178  1.41555  1.40506  1.38981  1.08577  1.41194  1.36973  1.41958  1.34416  1.40723  1.91982  1.06097  1.78576  1.8464  0.988612  0.989995  1.86214  0.991356  0.991894  1.3894  0.965142  1.51459  1.11133  1.11081  1.1094  1.11136  1.10856 |

**Table S13.** ADMET properties of the compounds **(2, 7-10, streptomycin).**

| Parameters | HL^2^  (2) | [Co(L^2^)_2_(H_2_O)_2_]  (7) | [Ni(L^2^)_2_(H_2_O)_2_]    (8) | [Cu(L^2^)_2_(H_2_O)_2_]  (9) | [Zn(L^2^)_2_(H_2_O)_2_]  (10) | Streptomycin |
| --- | --- | --- | --- | --- | --- | --- |
| ADMET-EXT-CYP2D6 | -0.0287512 | -0.550543 | -0.883202 | -2.10825 | -0.763312 | -5.2553 |
| ADMET-EXT-CYP2D6#prediction | False | False | False | False | False | False |
| ADMET-EXT-CYP2D6-applicability | Within Expected Range | ALogP out of range. Value: 11.654 | "ALogP out of range. Value: 10.256. | Within Expected Range | ALogP out of range. Value: 11.849. | ALogP out of range. Value: -6.291. |
| ADMET-EXT-CYP2D6-applicability#MD | 15.4855 | 19.3605 | 18.5921 | 17.0448 | 19.4159 | 22.8753 |
| ADMET-EXT-CYP2D6-applicability#MDpvalue | 5.73152e-07 | 1.90871e-11 | 1.46563e-10 | 9.11839e-09 | 1.64873e-11 | 2.20308e-15 |
| ADMET-EXT- hepatotoxic | -4.9044 | -4.0708 | -6.31206 | -3.88957 | -4.60086 | -17.8396 |
| ADMET-EXT- hepatotoxic #prediction | false | true | false | true | False | False |
| ADMET-EXT- hepatotoxic applicability | Within Expected Range | Within Expected Range | Within Expected Range | Within Expected Range | Within Expected Range | Within Expected Range |
| ADMET-EXT- hepatotoxic applicability #MD | 12.0612 | 16.2033 | 14.2674 | 14.6652 | 16.3122 | 10.115 |
| ADMET-EXT- hepatotoxic applicability #MDpvalue | 0.000125709 | 1.41175e-14 | 2.21007e-09 | 2.20274e-10 | 6.85483e-15 | 0.0697675 |
| ADMET absorption level |  |  |  |  |  |  |
| ADMET EXT PPB | 4.72576 | 1.90424 | 2.25217 | -2.02136 | 3.08467 | -55.1654 |
| ADMET-EXT-PPB #Prediction | true | true | true | true | true | False |
| ADMET-EXT-PPB- applicability | Within Expected Range | ALogP out of range. Value: 11.654 | ALogP out of range. Value: 10.256. | Within Expected Range | ALogP out of range. Value: 11.849. | Within expected ranges. |
| ADMET-EXT-PPB- applicability #MD | 13.8007 | 17.3415 | 17.8816 | 17.0851 | 16.6171 | 13.7233 |
| ADMET-EXT-PPB- applicability #MDpvalue | 0.000252154 | 1.38958e-13 | 2.17442e-15 | 9.29324e-13 | 2.62673e-11 | 0.000351132 |

**Table S14.** TOPKAT toxicity properties of the compounds **(2, 7-10, streptomycin).**

| Parameters | HL^2^  (2) | [Co(L^2^)_2_(H_2_O)_2_]  (7) | [Ni(L^2^)_2_(H_2_O)_2_]    (8) | [Cu(L^2^)_2_(H_2_O)_2_]  (9) | [Zn(L^2^)_2_(H_2_O)_2_]  (10) | Streptomycin |
| --- | --- | --- | --- | --- | --- | --- |
| Toxicity |  |  |  |  |  |  |
| TOPKAT mouse female NTP prediction | Carcinogen | Non-Carcinogen | Non-Carcinogen | Non-Carcinogen | Non-Carcinogen | Non-Carcinogen |
| TOPKAT mouse female NTP applicability | Within expected range | "ALogP out of range. Value: 11.654.  Molecular_Weight out of range. Value: 1092.4.  OPS PC8 out of range. Value: -4.9047.  OPS PC16 out of range. Value: -4.3574. | Molecular_Weight out of range. Value: 1097.2.  OPS PC8 out of range. Value: -6.4182. | Molecular_Weight out of range. Value: 1126.2.  OPS PC8 out of range. Value: -4.6407. | ALogP out of range. Value: 11.849.  Molecular_Weight out of range. Value: 1098.8.  OPS PC8 out of range. Value: -4.9769. | Num_H_Donors out of range. Value: 12. |
| TOPKAT mouse female NTP probability | 0.625591 | 0.587028 | 0.550619 | 0.543803 | 0.549052 | 0.434512 |
| TOPKAT mouse female NTP enrichment | 1.58969 | 1.49169 | 1.39918 | 1.38186 | 1.39519 | 1.10414 |
| TOPKAT mouse female NTP score | 1.74178 | 0.50874 | -0.874038 | -1.0745 | -0.92116 | -3.44061 |
| TOPKAT mouse male NTP prediction | Non-Carcinogen | Non-Carcinogen | Carcinogen | Carcinogen | Non-Carcinogen | Non-Carcinogen |
| TOPKAT mouse male NTP applicability | All properties and OPS components are within expected ranges. | "ALogP out of range. Value: 11.654.  Molecular_Weight out of range. Value: 1092.4. | Molecular_Weight out of range. Value: 1097.2.. | Molecular_Weight out of range. Value: 1126.2. | "ALogP out of range. Value: 11.849..  Molecular_Weight out of range. Value: 1098.8. | Num_H_Donors out of range. Value: 12. |
| TOPKAT mouse male NTP probability | 0.42625 | 0.537877 | 0.610885 | 0.610734 | 0.547999 | 0.263266 |
| TOPKAT mouse male NTP enrichment | 1.08352 | 1.36728 | 1.55286 | 1.55248 | 1.39301 | 0.669219 |
| TOPKAT mouse male NTP score | -3.27778 | -1.72938 | 0.449351 | 0.436277 | -1.54305 | -5.00385 |
| TOPKAT Rat female NTP prediction | Carcinogen | Non-Carcinogen | Non-Carcinogen | Non-Carcinogen | Non-Carcinogen | Non-Carcinogen |
| TOPKAT Rat female NTP applicability | within expected ranges. | ALogP out of range. Value: 11.654.  Molecular_Weight out of range. Value: 1092.4.  OPS PC6 out of range. Value: 5.5934.  OPS PC9 out of range. Value: -5.3979. | Molecular_Weight out of range. Value: 1097.2.  OPS PC6 out of range. Value: 6.1301.  OPS PC11 out of range. Value: 3.4435. | Molecular_Weight out of range. Value: 1126.2..  OPS PC6 out of range. Value: 4.7718.  OPS PC11 out of range. Value: 4.3999.  OPS PC18 out of range. Value: -3.3013. | "ALogP out of range. Value: 11.849.  Molecular_Weight out of range. Value: 1098.8.  OPS PC6 out of range. Value: 5.2805.  OPS PC9 out of range. Value: -4.8001. | Num_H_Donors out of range. Value: 12. |
| TOPKAT Rat female NTP probability | 0.532957 | 0.362645 | 0.451097 | 0.397491 | 0.479101 | 0.342611 |
| TOPKAT Rat female NTP enrichment | 1.17089 | 0.796721 | 0.991047 | 0.873276 | 1.05257 | 0.752707 |
| TOPKAT Rat female NTP score | 0.468191 | -4.95818 | -2.40325 | -3.98817 | -1.503 | -5.50403 |
| TOPKAT Rat male NTP prediction | Non- carcinogen | Non-Carcinogen | Non-Carcinogen | Non-Carcinogen | Non-Carcinogen | Non-Carcinogen |
| TOPKAT Rat male NTP applicability | Within range | ALogP out of range. Value: 11.654. Molecular_Weight out of range. Value: 1092.4.  OPS PC13 out of range. Value: -3.6061.  OPS PC18 out of range. Value: 4.6242. | "Molecular_Weight out of range. Value: 1097.2.  OPS PC13 out of range. Value: -3.3161. - | Molecular_Weight out of range. Value: 1126.2. | ALogP out of range. Value: 11.849.  Molecular_Weight out of range. Value: 1098.8.  OPS PC13 out of range. Value: -3.2461. | Num_H_Donors out of range. Value: 12. |
| TOPKAT Rat male NTP probability | 0.506498 | 0.504356 | 0.484446 | 0.548262 | 0.438405 | 0.46769 |
| TOPKAT Rat male NTP enrichment | 0.995328 | 0.991118 | 0.951992 | 1.0774 | 0.861517 | 0.919065 |
| TOPKAT Rat male NTP score | -2.55738 | -2.6008 | -2.99628 | -1.6698 | -3.86666 | -3.31927 |
| TOPKAT mouse female FDA | Single-Carcinogen | Non-Carcinogen | Non-Carcinogen | Non-Carcinogen | Non-Carcinogen | Non-Carcinogen |
| TOPKAT mouse male FDA | Non-carcinogen | Non-Carcinogen | Non-Carcinogen | Multi-Carcinogen | Non-Carcinogen | Non-Carcinogen |
| TOPKAT rat female FDA | Non - carcinogen | Non -Carcinogen | Non -Carcinogen | Single -Carcinogen | Non-Carcinogen | Single-Carcinogen |
| TOPKAT rat male FDA | Non-carcinogen | Non-Carcinogen | Non-Carcinogen | Non-Carcinogen | Non-Carcinogen | Non-Carcinogen |
| TOPKAT mouse female FDA none vs carcinogen prediction | carcinogen | Non-Carcinogen | Non-Carcinogen | Non-Carcinogen | Non-Carcinogen | Non-Carcinogen |
| TOPKAT mouse female FDA none vs carcinogen applicability | Within expected range | "ALogP out of range. Value: 11.654. Training min, max, mean, SD: -4.311, 10.955, 2.1947, 2.219.  Molecular_Weight out of range. Value: 1092.4. | Molecular_Weight out of range. Value: 1097.2. | Molecular_Weight out of range. Value: 1126.2.  OPS PC25 out of range. Value: -3.7917. | ALogP out of range. Value: 11.849.  Molecular_Weight out of range. Value: 1098.8.  OPS PC29 out of range. Value: -2.9446. | "ALogP out of range. Value: -6.291.  _Donors out of range. Value: 12.  Num_H_Acceptors out of range. Value: 19..  OPS PC1 out of range. Value: 5.7156.  OPS PC10 out of range. Value: 5.9837. |
| TOPKAT mouse female FDA none vs carcinogen probability | 0.246914 | 0.213366 | 0.207834 | 0.208073 | 0.205756 | 0.218582 |
| TOPKAT mouse female FDA none vs carcinogen enrichment | 0.77037 | 0.665703 | 0.648441 | 0.649188 | 0.64196 | 0.681976 |
| TOPKAT mouse female FDA none vs carcinogen score | -0.880244 | -3.78804 | -7.83867 | -4.7341 | -5.43426 | -3.12961 |
| TOPKAT mouse male FDA none vs carcinogen prediction | Non-carcinogen | Non-Carcinogen | Non-Carcinogen | Carcinogen | Non-Carcinogen | Non-Carcinogen |
| TOPKAT mouse male FDA none vs carcinogen applicability | Within range | "ALogP out of range. Value: 11.654.  Molecular_Weight out of range. Value: 1092.4.  OPS PC14 out of range. Value: -4.024. | Molecular_Weight out of range. Value: 1097.2. | Molecular_Weight out of range. Value: 1126.2. | "ALogP out of range. Value: 11.849.  Molecular_Weight out of range. Value: 1098.8. | ALogP out of range. Value: -6.291.  Num_H_Donors out of range. Value: 12..  Num_H_Acceptors out of range. Value: 19.  OPS PC1 out of range. Value: -5.669. |
| TOPKAT mouse male FDA none vs carcinogen probability | 0.224235 | 0.254404 | 0.196201 | 0.290245 | 0.237674 | 0.200232 |
| TOPKAT mouse male FDA none vs carcinogen enrichment | 0.761915 | 0.864426 | 0.666662 | 0.986209 | 0.807582 | 0.680357 |
| TOPKAT mouse male FDA none vs carcinogen score | -3.57174 | -2.08783 | -5.2212 | -0.587191 | -2.8802 | -4.96334 |
| TOPKAT Rat female FDA none vs carcinogen prediction | Non -Carcinogen | Non -Carcinogen | Non-Carcinogen | Carcinogen | Non -Carcinogen | Carcinogen |
| TOPKAT Rat female FDA none vs carcinogen applicability | Within range | "ALogP out of range. Value: 11.654.  Molecular_Weight out of range. Value: 1092.4.  OPS PC26 out of range. Value: 3.2822. | "Molecular_Weight out of range. Value: 1097.2. | Molecular_Weight out of range. Value: 1126.2. | ALogP out of range. Value: 11.849.  Molecular_Weight out of range. Value: 1098.8.  OPS PC26 out of range. Value: 3.4619. | "ALogP out of range. Value: -6.291.  Num_H_Donors out of range. Value: 12.  Num_H_Acceptors out of range. Value: 19.  OPS PC3 out of range. Value: 7.0962. |
| TOPKAT Rat female FDA none vs carcinogen probability | 0.26303 | 0.262542 | 0.224624 | 0.278695 | 0.232551 | 0.306939 |
| TOPKAT Rat Female FDA none vs carcinogen enrichment | 0.816891 | 0.815374 | 0.697611 | 0.86554 | 0.722231 | 0.953258 |
| TOPKAT Rat female FDA none vs carcinogen score | -1.94324 | -1.97313 | -4.6988 | -1.03425 | -4.04802 | 0.40701 |
| TOPKAT rat male FDA none vs carcinogen prediction | Non-Carcinogen | Non -Carcinogen | Non-Carcinogen | Non-Carcinogen | Non-Carcinogen | Non-Carcinogen |
| TOPKAT rat male FDA none vs carcinogen applicability | Within expected ranges. | "ALogP out of range. Value: 11.654.  Molecular_Weight out of range. Value: 1092.4. | Molecular_Weight out of range. Value: 1097.2..  OPS PC24 out of range. Value: -3.4102. | Molecular_Weight out of range. Value: 1126.2. | "ALogP out of range. Value: 11.849.  Molecular_Weight out of range. Value: 1098.8. | "ALogP out of range. Value: -6.291.  Num_H_Donors out of range. Value: 12.  Num_H_Acceptors out of range. Value: 19. |
| TOPKAT rat male FDA none vs carcinogen probability | 0.339922 | 0.255729 | 0.3126 | 0.296842 | 0.278425 | 0.266362 |
| TOPKAT rat male FDA none vs carcinogen enrichment | 1.01711 | 0.765189 | 0.935358 | 0.888207 | 0.8331 | 0.797004 |
| TOPKAT rat male FDA none vs carcinogen score | -0.582027 | -4.11389 | -1.61463 | -2.25314 | -3.04768 | -3.60105 |
| TOPKAT WOE prediction | Non-Carcinogen | Non- Carcinogen | Non-Carcinogen | Non-Carcinogen | Non-Carcinogen | Non-Carcinogen |
| TOPKAT WOE applicability | Within expected ranges. | "ALogP out of range. Value: 11.654.  Molecular_Weight out of range. Value: 1092.4.  OPS PC12 out of range. Value: -3.9881.  OPS PC13 out of range. Value: -3.0779. | Molecular_Weight out of range. Value: 1097.2.  OPS PC12 out of range. Value: -4.4566. | Molecular_Weight out of range. Value: 1126.2. | ALogP out of range. Value: 11.849.  Molecular_Weight out of range. Value: 1098.8.  OPS PC12 out of range. Value: -3.7314.  OPS PC13 out of range. Value: -2.9769.  OPS PC18 out of range. Value: -3.5126. | Num_H_Donors out of range. Value: 12.  Num_H_Acceptors out of range. Value: 19.  OPS PC2 out of range. Value: 5.2131.  OPS PC3 out of range. Value: 6.3712.  out of range. Value: 6.2272. |
| TOPKAT WOE probability | 0.503964 | 0.435798 | 0.463613 | 0.5173 | 0.459083 | 0.365262 |
| TOPKAT WOE enrichment | 0.978773 | 0.846384 | 0.900405 | 1.00467 | 0.891607 | 0.709392 |
| TOPKAT WOE score | -0.913527 | -2.71164 | -1.95396 | -0.581453 | -2.07478 | -4.85906 |
| TOPKAT carcinogenic potency TD50 mouse | 59.4872 | 0.027412 | 0.0508825 | 0.0204545 | 0.0435124 | 0.187551 |
| TOPKAT carcinogenic potency TD50 mouse unit | mg/kg body weight/day | mg/kg body weight/day | mg/kg body weight/day | mg/kg body weight/day | mg/kg body weight/day | mg/kg_body_weight/day |
| TOPKAT carcinogenic potency TD50 mouse applicability | Within expected ranges. | "Molecular_Weight out of range. Value: 1092.4.  OPS PC16 out of range. Value: 4.8872. | "Molecular_Weight out of range. Value: 1097.2. | "Molecular_Weight out of range. Value: 1126.2. | "Molecular_Weight out of range. Value: 1098.8. Training min, max, mean, SD: 30.026, 871.78, 225.83, 133.9.  OPS PC16 out of range. Value: 4.7857. | "ALogP out of range. Value: -6.291. Num_H_Donors out of range. Value: 12.  OPS PC2 out of range. Value: 9.8741.  OPS PC4 out of range. Value: 7.5455.  OPS PC20 out of range. Value: -4.5588. |
| TOPKAT carcinogenic potency TD50 rat | 51.99 | 0.0554432 | 0.0383039 | 0.000153513 | 0.125986 | 0.821895 |
| TOPKAT carcinogenic potency TD50 rat unit | mg/kg body weight/day | mg/kg body weight/day | mg/kg body weight/day | mg/kg body weight/day | mg/kg body weight/day | mg/kg_body_weight/day |
| TOPKAT carcinogenic potency TD50 rat applicability | Within expected range | Within expected range | Within expected ranges | Within expected ranges. | Within expected range | ALogP out of range. Value: -6.291. Num_H_Donors out of range. Value: 12.. |
| TOPKAT ames prediction | Non-Mutagen | Non-Mutagen | Non-Mutagen | Non-Mutagen | Non-Mutagen | Mutagen |
| TOPKAT Ames applicability | Within expected ranges. | Within expected ranges. | Within expected ranges. | Within expected ranges. | Within expected ranges. | Within expected range |
| TOPKAT Ames probability | 0.378282 | 0.221553 | 0.178102 | 0.195706 | 0.218209 | 0.79919 |
| TOPKAT Ames enrichment | 0.677473 | 0.396784 | 0.318967 | 0.350494 | 0.390795 | 1.43129 |
| TOPKAT Ames score | -10.1431 | -13.6084 | -14.7345 | -14.2621 | -13.6907 | 4.26304 |
| TOPKAT DTP prediction | toxic | Toxic | Toxic | Non- toxic | Toxic | Toxic |
| TOPKAT DTP applicability | Within the expected range | "ALogP out of range. Value: 11.654..  Molecular_Weight out of range. Value: 1092.4.  OPS PC20 out of range. Value: -3.3697..  OPS PC24 out of range. Value: 3.4177. | ALogP out of range. Value: 10.256.  Molecular_Weight out of range. Value: 1097.2.  OPS PC20 out of range. Value: -3.2014.  OPS PC24 out of range. Value: 3.1617. | Molecular_Weight out of range. Value: 1126.2.  OPS PC21 out of range. Value: -3.782. | "ALogP out of range. Value: 11.849.  Molecular_Weight out of range. Value: 1098.8.  OPS PC20 out of range. Value: -3.4233.  OPS PC24 out of range. Value: 3.6585. | Num_H_Donors out of range. Value: 12..  Num_H_Acceptors out of range. Value: 19.  OPS PC12 out of range. Value: -4.2052. |
| TOPKAT DTP probability | 0.641833 | 0.630826 | 0.645348 | 0.534561 | 0.611181 | 0.746452 |
| TOPKAT DTP enrichment | 1.22039 | 1.19946 | 1.22707 | 1.01642 | 1.1621 | 1.41931 |
| TOPKAT DTP score | 2.42503 | 2.1456 | 2.51429 | -0.346677 | 1.64627 | 5.15333 |
| TOPKAT rat oral LD50 | 0.440065 | 3.74402 | 3.75583 | 4.81114 | 2.09897 | 0.441623 |
| TOPKAT rat oral LD50 unit | g/kg body weight | g/kg body weight | g/kg body weight | g/kg body weight | g/kg body weight | g/kg_body_weight |
| TOPKAT rat oral LD50 applicability | Within expected range | Molecular_Weight out of range. Value: 1092.4. | Molecular_Weight out of range. Value: 1097.2. | Molecular_Weight out of range. Value: 1126.2. | Molecular_Weight out of range. Value: 1098.8. | "Num_H_Acceptors out of range. Value: 19.  Molecular_PolarSurfaceArea out of range. Value: 336.42.  OPS PC33 out of range. Value: 6.9874.  OPS PC35 out of range. Value: -8.0054.  OPS PC60 out of range. Value: -4.1517. |
| TOPKAT Rat maximum tolerated dose feed | 0.14918 | 0.012737 | 0.0410412 | 0.00262358 | 0.00900741 | 1.00019 |
| TOPKAT Rat maximum tolerated dose feed unit | g/kg body weight | g/kg body weight | g/kg body weight | g/kg body weight | g/kg body weight | g/kg_body_weight |
| TOPKAT Rat maximum tolerated dose feed applicability | Within expected range | "ALogP out of range. Value: 11.654.  Molecular_Weight out of range. Value: 1092.4.  OPS PC3 out of range. Value: 6.9493.  OPS PC5 out of range. Value: 6.0842.  OPS PC13 out of range. Value: 4.0542. | "ALogP out of range. Value: 10.256.  Molecular_Weight out of range. Value: 1097.2.  OPS PC3 out of range. Value: 6.5442.  OPS PC5 out of range. Value: 6.2458.  OPS PC13 out of range. Value: 4.2769. | Molecular_Weight out of range. Value: 1126.2. | "ALogP out of range. Value: 11.849.  Molecular_Weight out of range. Value: 1098.8.  OPS PC3 out of range. Value: 7.2049.  OPS PC5 out of range. Value: 5.5777.  OPS PC13 out of range. Value: 3.4756. | "ALogP out of range. Value: -6.291.  Num_H_Donors out of range. Value: 12.  Num_H_Acceptors out of range. Value: 19.  Molecular_PolarSurfaceArea out of range. Value: 336.42.  Molecular_PolarSASA out of range. Value: 559.72.  OPS PC2 out of range. Value: 9.748.  OPS PC12 out of range. Value: -2.8451.  OPS PC13 out of range. Value: 5.1033.  OPS PC14 out of range. Value: -2.2674. |
| TOPKAT Rat maximum tolerated dose Gavage | 0.00325218 | 1.72469e-08 | 7.12534e-09 | 2.36886e-07 | 2.95373e-07 | 8.49078e-06 |
| TOPKAT Rat maximum tolerated dose gavage unit | g/kg body weight | g/kg body weight | g/kg body weight | g/kg body weight | g/kg body weight | g/kg_body_weight |
| TOPKAT Rat maximum tolerated dose gavage applicability | "Molecular_Weight out of range. Value: 499.2. | ALogP out of range. Value: 11.654.  Molecular_Weight out of range. Value: 1092.4.  Num_H_Donors out of range. Value: 4.  Num_H_Acceptors out of range. Value: 11.  Num_AromaticRings out of range. Value: 4. Molecular_PolarSurfaceArea out of range. Value: 154.15.  OPS PC1 out of range. Value: 10.941..  OPS PC4 out of range. Value: 10.86. OPS PC5 out of range. Value: -4.1025. | ALogP out of range. Value: 10.256..  Molecular_Weight out of range. Value: 1097.2..  Num_H_Donors out of range. Value: 6.  Num_H_Acceptors out of range. Value: 12.  Num_AromaticRings out of range. Value: 4.  Molecular_PolarSurfaceArea out of range. Value: 153.72. OPS PC1 out of range. Value: 11.743.  OPS PC4 out of range. Value: 10.907. OPS PC5 out of range. Value: -4.3992. | Molecular_Weight out of range. Value: 1126.2.  Num_H_Donors out of range. Value: 6.  Num_H_Acceptors out of range. Value: 12.  Molecular_PolarSurfaceArea out of range. Value: 153.72.  OPS PC4 out of range. Value: 9.7407.  OPS PC5 out of range. Value: -4.8714. | ALogP out of range. Value: 11.849..  Molecular_Weight out of range. Value: 1098.8.  Num_H_Donors out of range. Value: 4.  Num_H_Acceptors out of range. Value: 9.  Num_AromaticRings out of range. Value: 4.  Molecular_PolarSurfaceArea out of range. Value: 144.57.  OPS PC1 out of range. Value: 10.395.  OPS PC4 out of range. Value: 10.569.  OPS PC6 out of range. Value: -2.8249. | ALogP out of range. Value: -6.291. |
| TOPKAT rat inhalational LC50 | 6,292.92 | 218.235 | 222.711 | 38.0161 | 244.629 | 0.32772 |
| TOPKAT rat inhalational LC50 unit | mg/m3/h | mg/m3/h | mg/m3/h | mg/m3/h | mg/m3/h | mg/m3/h |
| TOPKAT rat inhalational LC50 applicability | "OPS PC24 out of range. Value: -3.4937. | "ALogP out of range. Value: 11.654.  Molecular_Weight out of range. Value: 1092.4.  Num_AromaticRings out of range. Value: 4.  OPS PC1 out of range. Value: 9.7489.  OPS PC22 out of range. Value: 2.9813. | ALogP out of range. Value: 10.256.  Molecular_Weight out of range. Value: 1097.2.  Num_AromaticRings out of range. Value: 4.  OPS PC1 out of range. Value: 9.8697. | Molecular_Weight out of range. Value: 1126.2.  OPS PC7 out of range. Value: 8.2944. | ALogP out of range. Value: 11.849.  Molecular_Weight out of range. Value: 1098.8. Num_AromaticRings out of range. Value: 4.  OPS PC1 out of range. Value: 9.2452. | ALogP out of range. Value: -6.291. |
| TOPKAT chronic LOAEL | 0.022798 | 0.000661658 | 0.000506259 | 0.000115831 | 0.00115119 | 0.0234261 |
| TOPKAT chronic LOAEL unit | g/kg body weight | g/kg body weight | g/kg body weight | g/kg body weight | g/kg body weight | g/kg_body_weight |
| TOPKAT chronic LOAEL applicability | Within expected ranges. | "ALogP out of range. Value: 11.654. Training min, max, mean, SD: -4.271, 10.946, 2.3349, 2.023.  Molecular_Weight out of range. Value: 1092.4. Num_Atoms out of range. Value: 63. | Molecular_Weight out of range. Value: 1097.2.  Num_Atoms out of range. Value: 63. | Molecular_Weight out of range. Value: 1126.2.  Num_Atoms out of range. Value: 63. OPS PC33 out of range. Value: 4.1176. | ALogP out of range. Value: 11.849..  Molecular_Weight out of range. Value: 1098.8.  Num_Atoms out of range. Value: 63. | ALogP out of range. Value: -6.291. |
| TOPKAT Skin irritancy | None | None | None | Mild | None | Mild |
| TOPKAT Skin irritancy none vs irritant prediction | Non-irritant | Non-irritant | Non-irritant | Irritant | Non-irritant | Irritant |
| TOPKAT Skin irritancy none vs irritant applicability | Within expected range | "Molecular_Weight out of range. Value: 1092.4.  OPS PC11 out of range. Value: -5.8136. | Molecular_Weight out of range. Value: 1097.2.  OPS PC11 out of range. Value: -6.1159. | Molecular_Weight out of range. Value: 1126.2.  OPS PC23 out of range. Value: -4.4219. | Molecular_Weight out of range. Value: 1098.8.  OPS PC11 out of range. Value: -6.1556. | "Num_H_Donors out of range. Value: |
| TOPKAT Skin irritancy none vs irritant probability | 0.947247 | 0.954326 | 0.95305 | 0.996466 | 0.942471 | 0.977601 |
| TOPKAT Skin irritancy none vs irritant enrichment | 1.0286 | 1.03629 | 1.0349 | 1.08205 | 1.02341 | 1.06156 |
| TOPKAT Skin irritancy none vs irritant score | -1.95758 | -1.74828 | -1.78953 | 0.224561 | -2.07671 | -0.220179 |
| TOPKAT Skin sensitization | Strong | Strong | Strong | Weak | Strong | None |
| TOPKAT Skin Sensitization none vs sensitizer prediction | Irritant | Irritant | Irritant | Irritant | Irritant | Non-Irritant |
| TOPKAT Skin Sensitization none vs sensitizer applicability | within expected ranges. | Molecular_Weight out of range. Value: 1092.4.  OPS PC6 out of range. Value: -4.7155.  OPS PC7 out of range. Value: 8.1461. | Molecular_Weight out of range. Value: 1097.2.  OPS PC6 out of range. Value: -4.8083.  OPS PC7 out of range. Value: 8.2927. | Molecular_Weight out of range. Value: 1126.2.  OPS PC6 out of range. Value: -6.1109.  OPS PC7 out of range. Value: 8.5956. | Molecular_Weight out of range. Value: 1098.8.  OPS PC7 out of range. Value: 7.996. | ALogP out of range. Value: -6.291. |
| TOPKAT Skin sensitization none vs sensitizer probability | 0.746526 | 0.740371 | 0.751635 | 0.746594 | 0.748074 | 0.612839 |
| TOPKAT Skin sensitization none vs sensitizer enrichment | 1.08787 | 1.0789 | 1.09532 | 1.08797 | 1.09013 | 0.893059 |
| TOPKAT Skin Sensitization none vs sensitizer score | -0.924207 | -1.04636 | -0.820638 | -0.92283 | -0.893039 | -3.14349 |
| TOPKAT ocular irritancy | Moderate | Moderate | Moderate | Moderate | Moderate | Severe |
| TOPKAT ocular irritancy none vs irritant prediction | Irritant | Irritant | Irritant | Irritant | Irritant | Irritant |
| TOPKAT ocular irritancy none vs irritant applicability | Within expected range | Within expected ranges. | Within range | Within range | Within expected range | ALogP out of range. Value: -6.291. |
| TOPKAT ocular irritancy none vs irritant probability | 0.999948 | 0.999661 | 0.999958 | 0.99954 | 0.999578 | 0.999912 |
| TOPKAT ocular irritancy none vs irritant enrichment | 1.1756 | 1.17527 | 1.17561 | 1.17563 | 1.17517 | 1.17556 |
| TOPKAT ocular irritancy none vs irritant score | 2.03058 | 0.656919 | 2.17576 | 0.434829 | 0.49723 | 1.64522 |
| TOPKAT ocular irritancy mild vs moderate severe prediction | Moderate-severe | Moderate-Severe | Moderate-Severe | Moderate-Severe | Moderate-Severe | Multiple |
| TOPKAT ocular irritancy mild vs moderate severe applicability | Within expected ranges. | Molecular_Weight out of range. Value: 1092.4. | "Molecular_Weight out of range. Value: 1097.2. | Molecular_Weight out of range. Value: 1126.2..  OPS PC10 out of range. Value: -5.4918. OPS PC17 out of range. Value: 5.299. | Molecular_Weight out of range. Value: 1098.8. | ALogP out of range. Value: -6.291. |
| TOPKAT ocular irritancy mild vs moderate severe probability | 0.818509 | 0.826793 | 0.857972 | 0.838054 | 0.81743 | 0.694265 |
| TOPKAT ocular irritancy mild vs moderate severe enrichment | 1.18804 | 1.20006 | 1.24531 | 1.2164 | 1.18647 | 1.11999 |
| TOPKAT ocular irritancy mild vs moderate severe score | -0.18124 | 0.298541 | 2.10547 | 1.19654 | -0.237271 | 0.0889881 |
| TOPKAT aerobic biodegradability prediction | Non -Degradable | Non-Degradable | Non-Degradable | Non-Degradable | Non-Degradable | Degradable |
| TOPKAT aerobic biodegradability applicability | OPS PC18 out of range. Value: 4.6682. | Molecular_Weight out of range. Value: 1092.4.  OPS PC18 out of range. Value: 6.0433. | "Molecular_Weight out of range. Value: 1097.2.  OPS PC16 out of range. Value: 4.3479.  OPS PC18 out of range. Value: 5.8517. | Within the expected range | "Molecular_Weight out of range. Value: 1098.8.  OPS PC18 out of range. Value: 5.9905. | ALogP out of range. Value: -6.291 |
| TOPKAT aerobic biodegradability probability | 0.0903364 | 0.0634151 | 0.0534693 | 0.313955 | 0.0566191 | 0.66157 |
| TOPKAT aerobic biodegradability enrichment | 0.20705 | 0.145347 | 0.122551 | 0.719583 | 0.12977 | 1.51631 |
| TOPKAT aerobic biodegradability score | -12.2276 | -14.3732 | -15.3822 | -3.91058 | -15.0541 | 3.66768 |
| TOPKAT fathead minnow LC50 | 3.78726e-05 | 1.10479e-09 | 3.05202e-07 | 4.45248e-06 | 4.28976e-10 | 1,000.02 |
| TOPKAT fathead minnow LC50 unit | g/l | g/l | g/l | g/l | g/l | g/l |
| TOPKAT fathead minnow LC50 applicability | "OPS PC5 out of range. Value: -4.8113.  OPS PC15 out of range. Value: -3.0474. | "ALogP out of range. Value: 11.654..  Molecular_Weight out of range. Value: 1092.4. Num_H_Acceptors out of range. Value: 11.  Num_AromaticRings out of range. Value: 4. OPS PC3 out of range. Value: -7.4853. OPS PC5 out of range. Value: -6.0669..  OPS PC9 out of range. Value: -9.0408.  OPS PC11 out of range. Value: -4.8517. | ALogP out of range. Value: 10.256..  Molecular_Weight out of range. Value: 1097.2.  Num_H_Donors out of range. Value: 6.  Num_H_Acceptors out of range. Value: 12.  Num_AromaticRings out of range. Value: 4.  out of range. Value: -7.1169.  OPS PC5 out of range. Value: -6.3.  OPS PC9 out of range. Value: -10.119.  OPS PC11 out of range. Value: -4.5148. OPS PC16 out of range. Value: -4.195. | "Molecular_Weight out of range. Value: 1126.2.  Num_H_Donors out of range. Value: 6.  Num_H_Acceptors out of range. Value: 12. OPS PC9 out of range. Value: -7.6986.  OPS PC11 out of range. Value: -5.1968.  OPS PC12 out of range. Value: 5.1922. OPS PC17 out of range. Value: 3.4469. | "ALogP out of range. Value: 11.849.  Molecular_Weight out of range. Value: 1098.8.  Num_AromaticRings out of range. Value: 4.  OPS PC3 out of range. Value: -6.9454.  OPS PC5 out of range. Value: -6.0905. OPS PC9 out of range. Value: -8.377.  OPS PC11 out of range. Value: -4.7995.  OPS PC12 out of range. Value: 3.6958. | ALogP out of range. Value: -6.291. |
| TOPKAT daphnia EC50 | 0.150629 | 1.72785e-05 | 7.4125e-06 | 5.8945e-05V | 1.23907e-05 | 12.3818 |
| TOPKAT daphnia EC50 unit | mg/l | mg/l | mg/l | mg/l | mg/l | mg/l |
| TOPKAT daphnia EC50 applicability | within expected ranges. | ALogP out of range. Value: 11.654.  Molecular_Weight out of range. Value: 1092.4.  Num_Atoms out of range. Value: 63.  OPS PC18 out of range. Value: -8.421. OPS PC23 out of range. Value: -6.2148. | "Molecular_Weight out of range. Value: 1097.2.  Num_Atoms out of range. Value: 63.  OPS PC23 out of range. Value: -7.1493. | "Molecular_Weight out of range. Value: 1126.2.  Num_Atoms out of range. Value: 63.  OPS PC2 out of range. Value: 14.01.  OPS PC23 out of range. Value: -7.1825. | ALogP out of range. Value: 11.849. Molecular_Weight out of range. Value: 1098.8.  Num_Atoms out of range. Value: 63.  OPS PC18 out of range. Value: -9.2494. | ALogP out of range. Value: -6.291. |
